# Demystifying mercury geochemistry in contaminated soil–groundwater systems with complementary mercury stable isotope, concentration, and speciation analyses†

**DOI:** 10.1039/d1em00368b

**Published:** 2022-01-04

**Authors:** D. S. McLagan, L. Schwab, J. G. Wiederhold, L. Chen, J. Pietrucha, S. M. Kraemer, H. Biester

**Affiliations:** Institute for Geoecology, Technical University of Braunschweig 38106 Braunschweig Germany david.mclagan@mail.utoronto.ca h.biester@tu-braunschweig.de; Department of Physical & Environmental Sciences, University of Toronto Scarborough Toronto M1C1A4 Canada; Department of Environmental Geosciences, Centre for Microbiology and Environmental Systems Science, University of Vienna 1090 Vienna Austria

## Abstract

Interpretation of mercury (Hg) geochemistry in environmental systems remains a challenge. This is largely associated with the inability to identify specific Hg transformation processes and species using established analytical methods in Hg geochemistry (total Hg and Hg speciation). In this study, we demonstrate the improved Hg geochemical interpretation, particularly related to process tracing, that can be achieved when Hg stable isotope analyses are complemented by a suite of more established methods and applied to both solid- (soil) and liquid-phases (groundwater) across two Hg^2+^-chloride (HgCl_2_) contaminated sites with distinct geological and physicochemical properties. This novel approach allowed us to identify processes such as Hg^2+^ (*i.e.*, HgCl_2_) sorption to the solid-phase, Hg^2+^ speciation changes associated with changes in groundwater level and redox conditions (particularly in the upper aquifer and capillary fringe), Hg^2+^ reduction to Hg^0^, and dark abiotic redox equilibration between Hg^0^ and Hg(ii). Hg stable isotope analyses play a critical role in our ability to distinguish, or trace, these *in situ* processes. While we caution against the non-critical use of Hg isotope data for source tracing in environmental systems, due to potentially variable source signatures and overprinting by transformation processes, our study demonstrates the benefits of combining multiple analytical approaches, including Hg isotope ratios as a process tracer, to obtain an improved picture of the enigmatic geochemical behavior and fate of Hg at contaminated legacy sites.

## Abstract 2

A holistic multi-analyses (led by Hg stable isotope analysis), multi-media, multi-site approach to improving contaminated site Hg geochemistry, particularly process tracing.

## Graphical abstract

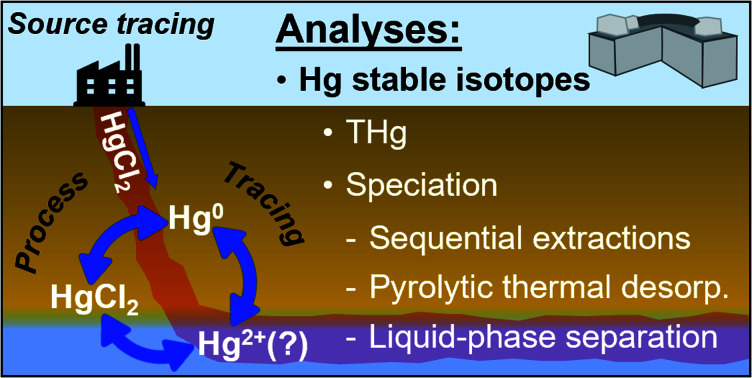

**Environmental significance**

Understanding mercury (Hg) biogeochemistry at contaminated sites is essential due to the risk they present to local human and environmental health. Furthermore, Hg is a contaminant of global concern due to its environmental mobility as soluble Hg^2+^ species or volatile elemental Hg. Thus, it is critical that we understand the stability of Hg and processes affecting this stability in soil–groundwater systems at these sites. Our ability to make such assessments is limited by analytical methods; our current knowledge of specific Hg processes and species occurring in the environment is often inadequate. We demonstrate how complementing existing methods with Hg stable isotope analyses improves our understanding of Hg biogeochemical cycling at contaminated sites and in the environment generally.

## 1 Introduction

Mercury (Hg) is a deviously elusive element that has and continues to effectively obscure the true nature of its exact environmental speciation, oxidation state, and the processes controlling its biogeochemical cycling from the intrigue of scientists. The use of Hg dates to the centuries BC and was employed by Chinese, Greeks, and Romans among other historical civilisations.^1,2^ We now understand Hg to be a persistent, bioaccumulative, and toxic (PBT) contaminant of global concern and its use has been heavily restricted or banned under the Minamata Convention on Hg.^3^ Critical to the degree of mobility, availability, and toxicity of Hg is its speciation; organic methylmercury (MeHg) is an acute neurotoxin and represents its most toxic form.^4,5^ Aside from organic species, elemental Hg (Hg^0^) and inorganic Hg^2+^ species are commonly encountered in the environment. Inorganic Hg^1+^ species can also occur but are generally believed to play a minor role in natural systems, mainly as shorter-lived intermediates in redox cycling between Hg^0^ and Hg^2+^ species.^5–7^

While the knowledge of oxidation state is relevant, this alone tells us little about the specific reaction mechanisms and exact compounds, associations, and complexations occurring, which are crucial to understand the nuances of Hg biogeochemical cycling in the environment. Identification of Hg^0^ across all environmental matrices is of particular importance due to its elevated volatility and long atmospheric residence time; long-range atmospheric transport of Hg^0^ is the primary means for the global distribution of Hg.^7–9^ Transport can also occur in aquatic media, and in this instance knowledge of specific speciation is important as the range of solubilities is vast. Cinnabar (HgS) is almost insoluble under most environmental conditions (solubility in water: ≈2 × 10^−24^ g L^−1^), Hg^0^ has a water solubility of ≈5 × 10^−5^ g L^−1^, while some mercuric salts are highly water soluble (*i.e.*, Hg^2+^ chloride (HgCl_2_): 66 g L^−1^).^7,10^

Another key controller of Hg mobility in soil and aquatic systems is the availability and nature of organic matter. Hg has a high affinity to both soil organic matter (SOM) or dissolved organic matter (DOM) particularly with reduced sulphur groups.^11–17^ Where SOM and DOM concentrations are low, mineral interactions become proportionally more relevant and Hg speciation is closely linked to iron redox processes in such systems.^18–20^ Clay minerals and metal oxides also provide potential sorption capacity for Hg, but the strength of these interactions can again be impacted by other factors such as specific mineralogy and organic surface coatings.^21,22^

Despite the importance of Hg speciation and reaction biogeochemistry, current analytical capabilities struggle to identify specific processes and Hg^2+^ compounds occurring in solid and aqueous matrices, which severely limits our understanding of Hg biogeochemical cycling in natural systems. Hg biogeochemical analyses have thus often focussed on the identification of Hg fractions that are “operationally defined” and unique to individual methods.^23,24^ The most commonly applied methods of achieving this being sequential extraction protocols (SEP), pyrolytic thermal desorption (PTD), and synchrotron-based X-ray absorption spectroscopy (XAS) techniques. All these methods have inherent strengths and weaknesses that result in limitations in the interpretation of results when used in isolation. The application of SEP is particularly problematic for samples with elevated fractions of Hg^0^,^25,26^ there can be matrix related artefacts affecting the distribution of Hg between the extracts,^25,27,28^ and the fractions of Hg removed in each extraction step are limited to “operationally-defined” pools rather than specific species.^24,29,30^ While PTD analyses are effective in identifying the presence of Hg^0^ (released at low temperatures) and Hg^2+^ sulphides and sulphates (released at high temperatures), most other Hg^2+^ species, often termed matrix-bound Hg^2+^, have overlapping release curves (released at moderate temperatures) making it difficult to discriminate specific species.^24,25,31,32^ XAS methods (*e.g.*, XANES and EXAFS) can provide arguably the highest degree of detail on specific species in a solid-phase sample (oxidation state, coordination geometry, and chemical bonding partners); however, synchrotron measurements are expensive, not widely available, and the detection limits can be prohibitively high (≈100 mg kg^−1^ Hg, at least for bulk sample techniques) restricting analyses to only highly contaminated samples.^24,27,33^

Hg stable isotope analyses are still relatively new and represent the multi-tool in Hg scientists' toolbox; the method has relevance to geochemical, atmospheric, hydrological, biological, and even cosmological sciences.^34–39^ Hg has seven stable isotopes that can be fractionated in the environment by mass-dependent fractionation (MDF) and mass-independent fractionation (MIF).^34,35^ This can be kinetic isotope fractionation, incomplete transformation of a pool of Hg resulting in the non-reacted pool being enriched in heavier isotopes, or equilibrium fractionation, equal forward and reverse reactions that drive heavier isotopes into “stronger bonding environments”.^37^ MIF is the deviation in measured stable isotope ratios from the theoretical MDF-based values. MIF of odd-mass Hg isotopes can be caused by both the nuclear volume effect (NVE) and the magnetic isotope effect (MIE).^34,35^ MIF of even-mass isotopes has also been observed, mostly in atmospheric samples, but a mechanistic understanding of this phenomenon is still lacking.^40,41^ Large odd-MIF effects found in environmental samples are generally associated with MIE during photochemical processes, but dark reactions have also been demonstrated to impart a MIF of smaller magnitude caused by the NVE under appropriate conditions.^42–44^ Although Hg stable isotope analysis does not allow the direct identification of specific Hg species, this assortment of different isotopes and fractionation processes provides detailed information about the biogeochemical history of Hg in an environmental sample and has the potential for both source and process tracing.^34,35,45,46^ We postulate that this novel ability of Hg stable isotopes to trace processes offers great potential to advance Hg geochemistry beyond the current state-of-the-art.

Hg contaminated sites provide unique opportunities to investigate and improve our knowledge of Hg biogeochemistry without concerns of low matrix concentrations of total Hg (THg) increasing analytical uncertainties that can occur at background sites.^47^ This is particularly pertinent for Hg speciation and stable isotope analyses as dividing the pool of THg by specific species, fractions, or isotopes intrinsically raises the required detection/quantification limits for the Hg analyses in the samples. Hence, they provide Hg scientists with the often termed “field laboratory” – sites to examine Hg biogeochemical processes in greater detail under “real-world” environmental conditions.^48,49^ From a less utilitarian viewpoint, Hg contaminated sites present acute and chronic risks to human and environmental health. It is essential that Hg speciation and stability and the processes impacting Hg speciation and stability are fully assessed to accurately gauge these risks at Hg contaminated sites.

Utilising multiple analytical methods on the same samples allows the examination of strengths and weaknesses of individual methods that have the potential to increase the quality and detail of information particularly related to Hg transformation processes, but also potentially to Hg speciation. Furthermore, soil–groundwater systems are coupled and highly interconnected, considering the solid and liquid-phases in isolation is atomistic and may result in suboptimal geochemical data interpretations. A holistic assessment should also account for variability in site geography, hydrology, and geology that affect Hg geochemistry. Hence, the purpose of this work is to demonstrate an improved explanation of geochemical assays of Hg at contaminated sites by utilising a suite of analytical techniques (THg concentration, Hg stable isotopes, Hg speciation, and complementary metadata) and data analysis methods in both solid- (soil) and liquid-phase (groundwater) samples.

It is our aim to improve our understanding of Hg geochemistry by examining our ability to assess (i) source information, (ii) transformation processes, (iii) speciation, and (iv) transport. We will apply this approach to examine two former wood kyanisation facilities (treatment of timber with ≈0.66% HgCl_2_ solution for preservation^50,51^) in Germany. These sites are ideal for examining Hg biogeochemistry in soil–groundwater systems due to the high solubility of HgCl_2_, which facilitated leaching into and transport in groundwater bodies. While a multi-method approach itself is not an entirely novel concept in Hg geochemical assessments, such approaches have rarely incorporated Hg stable isotope analyses, and not to the extent and scope of this study. We see Hg stable isotope analyses complemented by existing Hg concentration and speciation analyses as central in obtaining additional insight into the transport, transformation, and fate of mercury provided by previous Hg biogeochemical assays at these and other study sites. In addition, we aim to identify Hg transformation processes that have been shown to induce Hg stable isotope fractionations in laboratory-based experiments in environmental settings.

## 2 Methods

### 2.1 Site description

The two former kyanisation plants are located in the south–west of Germany and are contrasting in both their geology and hydrology. The location of wells and the former industrial buildings at site A and site B are presented in Section S1.† Site A is situated on the edge of a small town in a mountainous, forested area at ≈850 m above sea level. A small river flows through the middle of the site. The former industrial buildings from the kyanisation facility and their associated Hg contamination are spread across areas on both sides of the river. The industrial kyanisation treatment occurred at the site from ≈1900 to 1962, but timber treated with the HgCl_2_ solution was reported to have been stored on site until the early 1990s.^52^ A description of the site geology is provided in Section S1.† Three soil cores were taken at site A: soil core A1 (SCA1), soil core A2 (SCA2), and soil core A3 (SCA3). Both SCA1 and SCA2 were taken on the northern side of the small river that runs through the former industrial site; SCA1 is underneath the former sawmill and SCA2 is between that same building and the river. SCA3 (a shallow core down to 160 cm below the surface) was taken on the southern side of the river where the former treated wood drying area and kyanisation building were situated (Fig. S1.1†). Additionally, 11 topsoil samples were taken on the southern side of the river; these are labelled TSA1 – TSA11 (TSA11 also had a subsoil sample taken at ≈10–20 cm below the surface: labelled SSA11) and referred to generally as TSA samples (Fig. S1.1†). The depth values reported for soil samples are the middle of the range from which the sample was taken (*i.e.*, SCA3 −150 cm represents the homogenised material taken from between −140 and −160 cm). There were 15 groundwater wells sampled at site A, which are situated on both sides of the river running through the site (Fig. S1.1†). These include adjacent groundwater wells that sample from two different aquifers (unconfined and confined) that exist at this site (*i.e.*, WA2a and WA2b, respectively).

Site B is situated on the outskirts of a town (≈235 m above sea level) on the flood plains of the Rhine River. The study area is flat (mean gradient: 0.8%) and there is a single groundwater layer that has a uni-directional flow to the NNE. The kyanisation facility covered an area of approximately 9 ha and operated from 1904–1965.^53–55^ During this time, an estimated 10–20 t of Hg was released to the soil and groundwater system.^53,54^ A full description of the site geology is provided in Section S1.† The Hg contamination plume in the aquifer has been previously reported to extend approximately 1.3 km from the former industrial facility.^54^ Persistent below average rainfall during the sampling period in this area led to declining and low (lowest levels in previous 17 years) groundwater levels during the sampling campaign. Groundwater depths for the different wells at site B are presented in Sections S3 and S4† describes the mean historical and sampling period climate graphs at nearby weather stations.

Three soil cores were also taken at site B: soil core B1 (SCB1), soil core B2 (SCB2), and soil core B3 (SCB3). SCB1 is beneath the former kyanisation building, SCB2 is at the edge of the former industrial facility land, where contaminated surface materials were reportedly deposited to construct an embankment, and SCB3 is ≈600 m along the contaminated groundwater plume from SCB1, where there were no known contamination inputs at the top of the soil profile (Fig. S1.2†). Solid-phase samples were then transported in closed polypropylene containers from the field to the laboratory and stored at 4 °C until analysis or extraction. All solid-phase THg concentrations are reported on a dry weight (dw) basis. Fig. S1.2† shows the locations of the 17 groundwater wells sampled at Site B. These extend along the groundwater Hg plume from the site of the former kyanisation building (WB3) to a cluster of four wells (WB23, WB24, WB17, and WB29) at the end of the plume (≈1000 m from WB3), where THg concentrations in the groundwater reach background levels (0.1–0.5 μg L^−1^)^54^ (Fig. S1.2†).

Groundwater samples at both sites were extracted with a groundwater pump (Comet-Combi; Comet-Pumpen) using 10 mm inner diameter PTFE tubing. Well volume was exchanged three times before sampling occurred. Sampled groundwater for Hg liquid-phase speciation was collected in cleaned (soap-free distilled water dishwasher at 60 °C, triple rinsed with distilled water, triple rinsed with sample water before collection) 300 mL polypropylene bottles. Sample bottles were filled to capacity with minimal possible headspace to minimise gas-phase exchange. Sample bottles were then stored at 4 °C until return to laboratory for processing (<96 h for all samples). Samples for THg and Hg stable isotopes were collected in 50 mL polypropylene vials, stabilised with 1 vol% bromine–chloride (BrCl, prepared according to Bloom *et al.*^26^) after filtration with 0.45 μm cellulose acetate (CA) membrane filters in the field and transported to the laboratory where they were stored at 4 °C until analysis. A full description of groundwater well locations, sampling times, and methods is provided in Section S1.†

### 2.2 Analytical procedures

#### 2.2.1 Total Hg (THg) analyses

Solid-phase (soil) THg analyses first required extraction by modified aqua regia digestion. ≈0.1 g of soil was added to 12 mL of adapted aqua regia (8 mL HCl_(conc.)_, 3 mL HNO_3(conc.)_, and 1 mL BrCl) in 50 mL PP centrifuge tubes covered with perforated parafilm and then laterally agitated at 170 rpm for 18–24 h. Samples were then diluted with 36 mL of deionised water (18.2 MΩ cm), centrifuged at 3000 rcf, and supernatant filtered through 0.45 μm CA filters. THg concentration of these extracts was determined by a DMA80-L cold vapour atomic absorption/fluorescence spectrometer (CV-AAS/AFS; Milestone Srl) using online Hg^2+^ reduction with SnCl_2_. Liquid-phase samples for THg analyses were stabilised with 1% BrCl in the field. BrCl was neutralised with 0.2% NH_2_OH–HCl immediately prior to analysis. THg concentrations were determined as for the solid-phase THg samples by CV-AAS/AFS.

#### 2.2.2 Hg speciation analyses

**Fig. 1:**
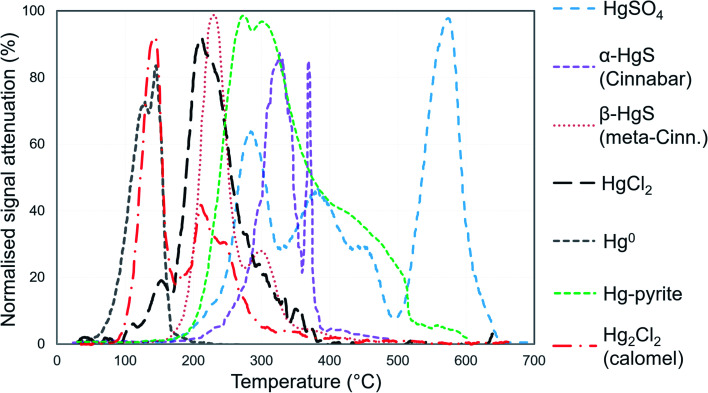
Mean (between 2 and 10 replications), normalised PTD release curves of standards for Hg species. All standards were prepared in silica (SiO_2_) matrix. Species are below 100% normalised maximum attenuation due to variability in signal of replicates.

Solid-phase speciation was assessed with pyrolytic thermal desorption (PTD) and sequential extraction protocols (SEP) analyses. A detailed PTD method summary is given in Biester and Scholz.^25^ Briefly, undried, unprocessed soil samples were heated at 1 °C per second in a N_2_ gas flow until 650 °C. The combustion stream was passed through a AAS detector (254 nm). The absorption curves were compared to the absorption curves for a series of Hg reference materials (Hg^0^, HgCl_2_, Hg_2_Cl_2_ (calomel), cinnabar: α-HgS, meta-cinnabar: β-HgS, and Hg^2+^-sulphate: HgSO_4_) in SiO_2_ matrix to infer the species or “fractions” of Hg present in the samples (Fig. 1).

We identified individual peaks using peak fitting software based on Gaussian (normal) peak shapes (OriginPro 2018 v9.5). We acknowledge the elevated difficulty and uncertainty in identification and quantification of individual Hg^2+^ species (release peaks > 175 °C) due to species with overlapping peaks and possible matrix effects that may alter peak release temperature and peak shape (non-Gaussian).^25,31,32^ We deem peak fitting, identification, and quantification of Hg^2+^ species to be qualitative analyses (see Section S5† for details). However, the high volatility of Hg^0^ results in it being released early in PTD analyses (<175 °C) with little-to-no overlap with Hg^2+^ species (Fig. 1); therefore, Hg^0^ can be quantitatively or at least semi-quantitatively assessed. Details of the nature of these assessments are given in Section S2.† The reported PTD signal attenuation data are normalised to the maximum attenuation in each measured replicate and then presented as the mean value of all measured replicates for each sample (between 2 and 10 replications).

SEP speciation analyses were also investigated in selected solid-phase samples. This method followed what is described in Brocza *et al.*^55^ Briefly, F1 is the water extractable fraction (deionised water: 18.2 MΩ cm) and targets soluble species such as HgCl_2_ and DOM bound Hg.^26,55^ F2 targets “labile, bioavailable Hg” weakly sorbed species such as those associated with carbonates using a weak acid (0.5 M HNO_3_).^55,56^ F3 uses a stronger acid (6 M HNO_3_) to extract more tightly bound species.^55,57^ F4 uses the strongest acid strength (aqua regia) to remove the remaining most stable, sulphide-bound species (α-HgS and β-HgS).^26,30^ Please note that compared with the widely used SEP by Bloom *et al.*,^26^ we omitted the 1 M KOH step targeting organo-chelated Hg species due to their low abundance in most of our field samples, increased the acid strength of the F2 step to account for the high buffer capacity of the carbonaceous sample material, and decreased the molarity of the HNO_3_ extract from 12 M to 6 M to avoid potential dissolution of very fine-grained HgS phases following Hall *et al.*^57^ as already discussed in Brocza *et al.*^55^ Tests were run that included this omitted step on selected samples. The relative Hg fraction of the omitted step was low, which supports the hypothesis that sorption to SOM plays a minor role in these soils (see Section S2† for details on these tests).^54^

Some of the “species” identified by both PTD and SEP analyses are not individual Hg species but rather two or more specific species or “fractions” that behave similarly under varying temperature and extractant strength, respectively. “Fractionation” analysis has also been used to describe these methods.^30,58^ We acknowledge this but have chosen to use the term “speciation” analyses for both PTD and SEP for consistency with the majority of the literature and to avoid confusion with Hg stable isotope fractionation also investigated in this study.

Mercury species in liquid-phase samples (groundwater) were determined by the method outlined by Bollen *et al.*^54^ and Richard *et al.*^20^ using a Hg-254 Analyzer (Seefelder Messtechnik). This method produces a total of four distinct fractions in addition to THg: (i) Hg^0^ (purged from untreated sample), (ii) dissolved inorganic Hg^2+^ (Hg^2+^A; purged after reduction with SnCl_2_ treatment; *e.g.* HgCl_2_); (iii) DOM-bound Hg^2+^ (Hg^2+^B; purged after BrCl and SnCl_2_ treatment), and (iv) particulate Hg (Hg-part; difference between total filtered and total unfiltered). Liquid-phase speciation analyses are deemed qualitative (see Section S2†).

#### 2.2.3 Hg stable isotope analyses

Hg stable isotopes were measured on solid-phase total digests and extracts as well as groundwater samples. Low concentration samples (<1 mg kg^−1^) were pre-concentrated using the DMA-80 Hg analyser, similar to the method described in Enrico *et al.*^59^ Briefly, multiple boats of the same sample were heated to 750 °C with an adjusted temperature ramp compared to standard measurements. Hg was then passed through the catalyser tube and multiple boats were purged into a single 5 mL inverse aqua regia trap that replaced 1 mL of the HCl with BrCl as proposed by Janssen *et al.*^60^ THg concentration and acid strengths were diluted to assure concentration and matrix matching during the isotope analysis.

Measurements were made with a Nu Plasma II (Nu Instruments) multicollector inductively coupled plasma mass spectrometer (MC-ICP-MS) connected to a HGX-200 cold vapour generator (Teledyne Cetac). Instrumental mass bias was corrected by standard sample bracketing with NIST-3133 and Tl doping using NIST-997 introduced by a Aridus 2 desolvating nebulizer (Teledyne Cetac). Mass-dependent fractionation (MDF) uses *δ* notation and is reported as permil (‰) values relative to NIST-3133 according to eqn (1):1
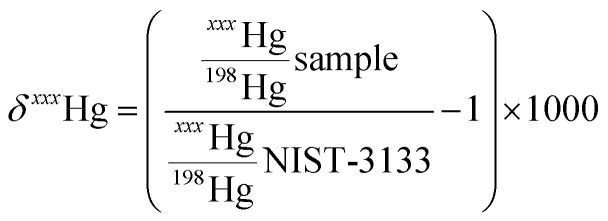


Mass-independent fractionation (MIF) uses Δ notation and describes fractionation away from the expected MDF according to eqn (2)–(5):

2 Δ^199^Hg = *δ*^199^Hg − (*δ*^202^Hg × 0.2520)

3 Δ^200^Hg = *δ*^200^Hg − (*δ*^202^Hg × 0.5024)

4 Δ^201^Hg = *δ*^201^Hg − (*δ*^202^Hg × 0.7520)

5 Δ^204^Hg = *δ*^204^Hg − (*δ*^202^Hg × 1.4930)

Our “in-house” secondary standard, *ETH Fluka*, was used to assess the accuracy and precision of measurements. Results (see Section S2†) were consistent with previous studies in this and other laboratories and details are provided in Section S2.†^55,61–66^ Analytical precision (2 SD) is reported with the measured values based on the standards run during each session (see results and discussion and Sections S2, S3, S6, and S7†). No significant even-mass MIF anomalies (Δ^200^Hg and Δ^204^Hg) outside of the analytical precision were generally detected in the samples of this study.

#### 2.2.4 Complementary analyses

Solid-phase total carbon (TC) and organic carbon (OC) (Section S8†), soil pH (Section S6†), major metal cations (Section S9†) and liquid-phase pH, redox, conductivity, dissolved oxygen (DO) content, and temperature (Section S3†) were measured and details of these methods and results can be found in the listed sections of the supplement.

#### 2.2.5 Quality assurance and quality control

Extensive quality assurance and control procedures were conducted across all analyses. A detailed summary of these procedures is provided in Section S2.†

## 3 Results and discussion

### 3.1 Source tracing

When examining legacy sites contaminated by Hg, it is essential to identify and characterise the location of point or area source(s) as both the magnitude and speciation of the initial contamination inputs are strongly linked to the stability of Hg and its ability to undergo different transformation processes at the sites.^67^ The most obvious means of identifying source Hg at contaminated sites is *via* “grey” and peer-reviewed literature on the location of historical Hg usage. This has the potential to detail input locations, historical use and contamination inventories, and composition of Hg bearing compounds used during the active industrial period. This is empirically supported by measuring THg concentrations in soil and groundwater across the sites to confirm these “hot spots” of elevated Hg concentrations and identify any additional areas of concern. Additionally, attempts at identifying the Hg isotopic signature of the initial contamination to assess transport and mixing/dilution away from source using source apportionment or end member mixing modelling have been made previously, an application of Hg stable isotope analyses we will also examine here.^63,68^

#### 3.1.1 Source tracing with total Hg concentration

**Fig. 2:**
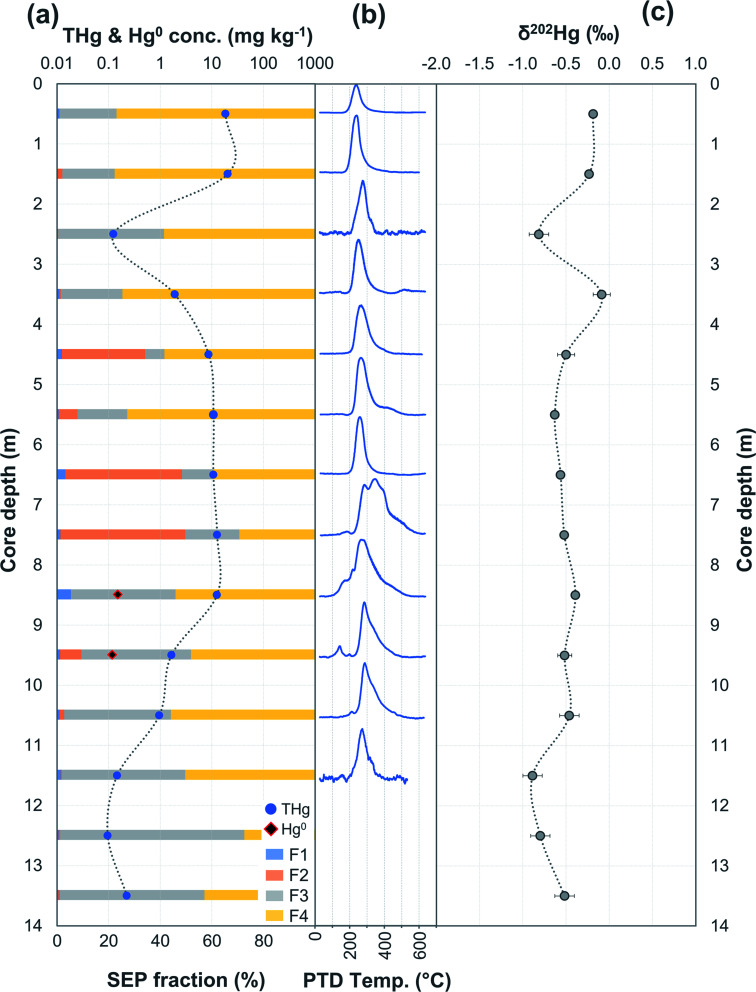
Multi-analyses summation of SCA1 solid-phase material. (a) THg and Hg^0^ concentration (logarithmic scale) and SEP speciation data. (b) PTD speciation data – all signal attenuations are normalised to the maximum attenuation for each sample. (c) *δ*^202^Hg (MDF) stable isotope data of the bulk sample. Aquifer depth could not be estimated as there were no adjacent wells to this soil core at the same elevation.

**Fig. 3:**
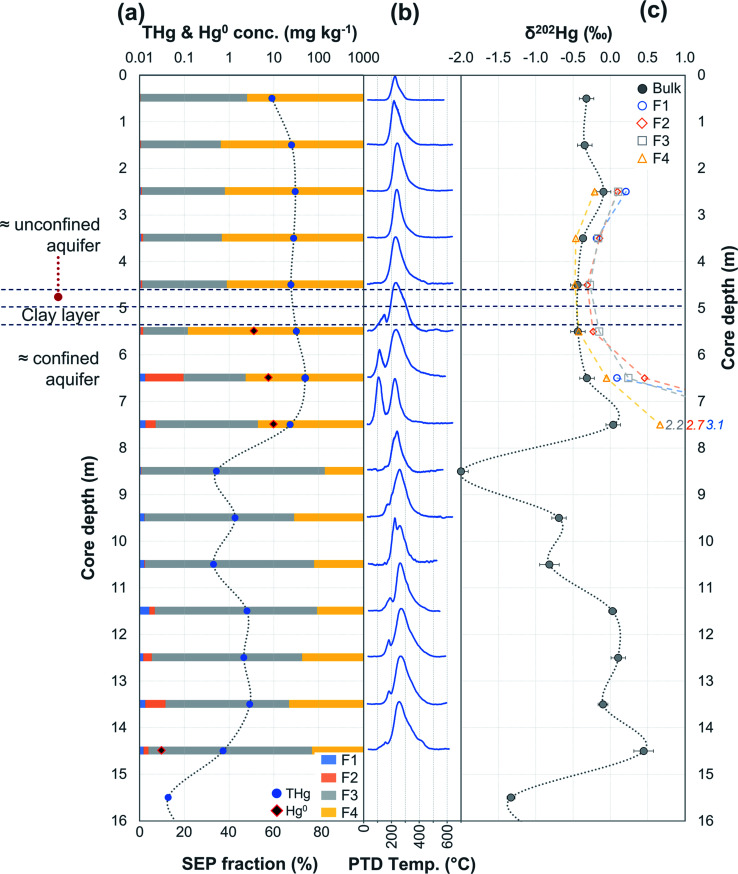
Multi-analyses summation of SCA2 solid-phase material. (a) THg and Hg^0^ concentration (logarithmic scale) and SEP speciation data. (b) PTD speciation data – all signal attenuations are normalised to the maximum attenuation for each sample. (c) *δ*^202^Hg (MDF) stable isotope data of bulk sample and SEP extracts. This core extends to −19.5 m; however, THg concentration are relatively constant and low (0.09 ± 0.03 mg kg^−1^) and *δ*^202^Hg values are relatively constant at −1.0 ± 0.2‰ for samples between −16.5 and −19.5 m (see Table S6.2† for data below 16 m).

At both sites, the highest dry weight (dw) THg concentrations in soils/solid-phase materials were found below the locations of the former kyanisation buildings where HgCl_2_ was applied or contaminated materials processed. THg concentrations in topsoils from the area of the former kyanisation building and wood drying areas on the southern side of the river at site A were variable and ranged from 0.45–319 mg kg^−1^ (mean: 62.3 mg kg^−1^; median: 10.6 mg kg^−1^; *n* = 11; Table S6.4†). There was no observable spatial pattern in these topsoil THg concentrations suggesting heterogeneously mixed surface materials associated with site constructions/demolitions and potentially irregular surfaces losses (reduction/volatilisation). There was little evidence of observable soil layering or horizons of SCA3 suggesting the depth of the soil disturbance/deposited construction materials extends beyond the depth of this 1.6 m core. Soil cores from the northern side of the river were below (SCA1) or adjacent (SCA2) to the former sawmill. There is potential for Hg contamination in this area to have slightly different speciation and input mechanisms into the soil–aquifer system as contamination is associated with the processing (*i.e.*, cutting, trimming, sanding, *etc.*) of HgCl_2_ treated timber rather than the treatment process itself. This and the terrain differences (including surface reshaping) are the likely explanation for lower THg concentrations in these cores (Fig. 2 and 3).

**Fig. 4:**
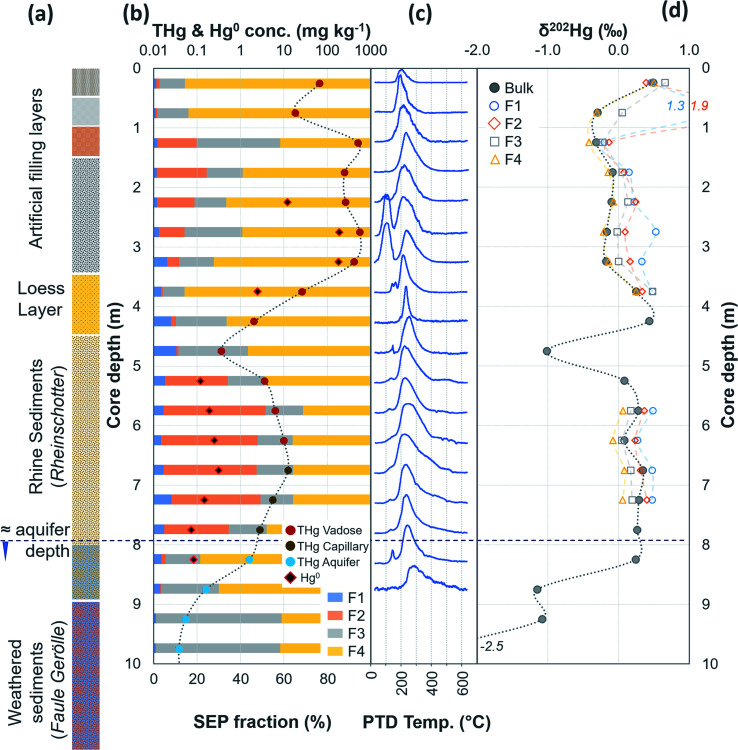
Multi-analyses summation of SCB1 solid-phase material. (a) Description of approximate soil core layer profile is based on our own visual characterisation and literature descriptions.^53–55^ (b) THg and Hg^0^ concentration (logarithmic scale) and SEP speciation data. (c) PTD speciation data – all signal attenuations are normalised to the maximum attenuation for each sample. (d) *δ*^202^Hg (MDF) stable isotope data of bulk sample and SEP extracts. This core extends to −17.25 m; however, THg concentrations are relatively constant and low (0.04 ± 0.03 mg kg^−1^) and *δ*^202^Hg values are also relatively constant and highly negative (−2.4 ± 0.3‰) for samples between −10.25 and −17.25 m (see Table S6.5† for data below 10 m).

Hg concentrations were higher in subsurface samples than surface samples in all soil cores at both sites and when comparing the subsurface sample SSA11 (THg: 41.5 mg kg^−1^) to topsoil sample TSA11 (THg: 2.8 mg kg^−1^) (Section S6†). The maximum THg concentration of SCA1, SCA2, and SCA3 were 20.2 (at −1.5 m; Fig. 2), 50.0 (at −6.5 m; Fig. 3), and 133 (at −1.5 m; Table S6.3†) mg kg^−1^. The maximum solid-phase concentration at site B was 562 mg kg^−1^ at −2.75 m in SCB1, which is below the former kyanisation building (Fig. 4). There is evidence of minor surface contamination in SCB2 and discussions with consulting agencies working at this site suggest contaminated materials were used to construct a small embankment in the area of this core. Nonetheless, THg concentrations were significantly higher in the aquifer than the vadose zone in this core (*p* < 0.001). These data suggest Hg leaching down soil profiles (see Section 3.2.4) and/or removal or burial of surface material from industrial period has occurred at both sites.

At both site A and site B, maximum liquid-phase (groundwater) concentrations are spatially offset from the maximum solid-phase concentrations and the location of former industrial buildings. At site A, the highest liquid-phase THg concentrations were consistently observed in WA7, WA10a, and WA10b on the northern side of the river (Table S3.1†) and are suggested to be down-aquifer from the former sawmill.^20^ Richard *et al.*^20^ also reported peak THg concentrations in well WA7 (labelled W2 in their study). All wells on the southern side of the river at site A had consistently lower THg concentrations than WA7, WA10a, and WA10b.

Similarly at site B, the highest groundwater THg concentrations were not observed at wells closest to the former kyanisation building (WB3 or WB7), but further along the relatively uniform groundwater flow path in wells WB10, WB8, WB19 and WB20, the latter of which (WB20) is located ≈700 m from the former kyanisation building. Data from both sites supports the previously posited notion of soluble Hg leaching down the soil profiles and being transported within the aquifer.^54^ This will be discussed in more detail in Section 3.2.

#### 3.1.2 Source tracing with stable Hg isotopes

Some studies have attempted to use the mean measured MDF and MIF values from published Hg ore minerals (cinnabar). Grigg *et al.*^63^ (see their ESI) reported mean *δ*^202^Hg of −0.65 ± 0.56‰ and Δ^199^Hg of 0.01 ± 0.10‰ (1 SD). Sun *et al.*^69^ reported similar *δ*^202^Hg values for cinnabar ores (−0.66 ± 0.73‰; 1 SD) and a mean *δ*^202^Hg of −0.38 ± 0.34‰ (1 SD) for commercial liquid Hg^0^. However, if we are to accept the mean MDF, then the associated variance must also be appropriately described. Furthermore, the HgCl_2_ solution used at these kyanisation facilities had to be produced from a commercial Hg^0^ stock derived from cinnabar ore (that we do not have a specific, single *δ*^202^Hg value for). We must consider the potential for the applied HgCl_2_ solution to be enriched in heavier isotopes compared to the Hg^0^ stock due to possible Hg^0^ loss (evaporation)^35,70^ in HgCl_2_ production or use (*i.e.*, partial photoreduction of HgCl_2_ and evaporation of Hg^0^). Thus, it is not possible to define the isotopic MDF signature of the Hg stock used at these facilities except to say its *δ*^202^Hg value is likely negative and in the range of ≈−1.2 to −0.1‰.

The regional background *δ*^202^Hg value at both sites appears to be depleted in heavier isotopes to a greater extent. A fine fraction (<2 mm) sediment sample collected ≈1 km upstream of the contaminated area at site A (THg concentration: 0.006 mg kg^−1^ dw) had a low *δ*^202^Hg of −2.27 ± 0.11‰. Although these are river sediments and not soils, their inputs are erosion of catchment soils upstream from the contaminated site; and hence we expect the *δ*^202^Hg values of these uncontaminated soils to be similar to the highly negative sediment material. Similarly, weathered sedimentary deposits of the lower part of the aquifer in SCB1 that we suggest are unaffected by surface contamination (THg: 0.075 mg kg^−1^ dw; *δ*^202^Hg: −2.3 ± 0.4‰, 1 SD; Table S6.5†), and the vadose zone of SCB3 (samples above −5 m), an area of site B with neither industrial Hg surface contamination nor inputs from the contaminated aquifer are highly enriched in lighter isotopes (THg: 0.08 ± 0.04 mg kg^−1^; *δ*^202^Hg: −2.77 ± 0.84‰, 1 SD). Even considering the negative end of the uncertainty range of the mean *δ*^202^Hg value of Hg ore minerals, the regional background MDF signature based on these samples unaffected by industrial contamination is more negative. Therefore, it is possible to conduct a general source appropriation or mixing modelling using a multidimensional analysis that includes THg concentration and *δ*^202^Hg. There is a significant negative relationship between *δ*^202^Hg and 1/THg that confirms more contaminated samples generally exhibit less negative *δ*^202^Hg values (Fig. 7). Nonetheless, any kinetic (or equilibrium) transformation process (*i.e.*, sorption of liquid-phase Hg^2+^ to the solid-phase; see Section 3.2.1) will fractionate Hg isotopes to favour lighter isotopes in the reacted or “products” pool. Due to isotope fractionations caused by *in situ* transformation processes and the large range of possible *δ*^202^Hg values of Hg stock material we can only recommend using such source appropriation in general terms and not on a more quantitative, sample-per-sample basis.

The MIF signature of global Hg ore minerals is more constrained; and hence, we can estimate the potential MIF industrial source signature more accurately. Mean Δ^199^Hg MIF signatures from individual soil cores, topsoils, and groundwater wells range from −0.11 to 0.00‰ (Sections S3 and S6†). In addition to the global mean, Grigg *et al.*^63^ also report the mean Δ^199^Hg MIF from European commercial ores as being more negative −0.06 ± 0.11‰ and suggest this value is more appropriate for European Hg usage. Considering our observed Δ^199^Hg values fall on either side of the European ore value, it may also be the most appropriate MIF industrial source signature at our sites. We generally observed only relatively small Δ^199^Hg and Δ^201^Hg variations in our samples. Mean Δ^199^Hg values with 1 SD range at site A were −0.01 ± 0.06‰ in the solid-phase materials and −0.07 ± 0.02‰ in groundwater. At site B these values were −0.03 ± 0.05‰ (solid-phase) and −0.10 ± 0.02‰ (groundwater), respectively. These data include samples with very negative *δ*^202^Hg values characteristic for “background” Hg. Thus, we could not define different MIF mixing endmembers in our dataset (see Fig. S6.1† for plot of Δ^199^Hg against *δ*^202^Hg in all solid phase samples).

Overall, characterisation of the Hg isotopic source signature at legacy contaminated sites remains a challenge. It is difficult to obtain and characterize the isotopic signature of the Hg stock (with potentially variable sources),^63,69^ and on-site transformation processes (including during preparation and use of HgCl_2_ solution from Hg^0^ stock) are likely to have shifted isotopic source signatures of measured samples away from that of the stock material. It is likely Hg stable isotopes are a more effective tool for tracing processes rather than sources at our field site and their application in the characterisation of Hg geochemical processes will be examined in the next section.

### 3.2 Process tracing

#### 3.2.1 Sorption of HgCl_2_ to the solid-phase

Hg sorption in soils is chiefly thought to be associated with SOM and in particular thiol- and other reduced sulphur groups.^11,12,72^ Richard *et al.*^20^ identified areas of site A with increased OC content likely associated with historical peat lenses. Nonetheless, the OC content measured in the solid-phase was ≤1.0% for samples from SCA1, SCA2, and SCA3 (Table S8.1†). OC was measured in SCB2 and was <0.5% in all layers except the poorly permeable loess layer (0.8–1.3% OC; Table S8.1†), which agrees well with measurements by Bollen *et al.*^54^ at the same site and their assessment that SOM is only contributing a small fraction to Hg solid-phase sorption. Even though we cannot completely exclude an involvement of SOM in Hg binding at our field sites, especially in some heterogeneously distributed localised areas in the subsurface, the influence of SOM on Hg is less than at most contaminated sites that have more elevated SOM. Non-SOM soil components are likely to play a dominant role in controlling Hg binding and solid-solution equilibria in our case.

##### 3.2.1.1 Hg^2+^ sorption to mineral surfaces

The first process driving the initial uptake of soluble HgCl_2_ to the low SOM solid-phase materials, particularly below areas with surface contamination, is likely to be the sorption of HgCl_2_ (or other Hg^2+^ species after HgCl_2_ transformation or dissociation) to surfaces of solid-phase Fe (*i.e.*, magnetite, goethite, pyrite, ferrihydrite)^73–75^ and Mn (*i.e.*, lithiophorite, birnessite)^76,77^ minerals. This process is suggested to occur chiefly through adsorption of the Hg^2+^ species to variable charge hydroxylated mineral surfaces.^77^ It has been suggested this sorption would require species transformation or partial dissociation of HgCl_2_.^75,78,79^ However, Brocza *et al.*^55^ demonstrated that Hg sorbed to the highly contaminated solid-phase material from the upper vadose zone in a core adjacent to SCB1 can be continuously released by sequential (or repeated) water extractions; even after seven consecutive extractions steps the liquid-phase THg concentration of the extract remained elevated (>0.1 mg L^−1^ or 20% of initial water extract liquid-phase THg concentration and above Hg^0^ solubility). Although it has been postulated that HgCl_2_ contributes little to solid-phase sorption (based on theoretical models and controlled mineralogical laboratory experiments, some that did not utilise HgCl_2_ input solution),^75,78,80^ sorption of this species to mineral surfaces and/or potential diffusion (and slow release) of HgCl_2_ into (and out of) mineral matrices would present a simple explanation of the sequential water extraction results from Brocza *et al.*^55^ HgCl_2_ sorption or matrix diffusion may be more plausible in complex environmental systems, particularly at these sites dominated by the original industrial inputs of HgCl_2_, than in theoretical or controlled single mineral systems.

HgCl_2_ could also dissociate to a HgCl^+^ ion under liquid-phase equilibrium with the ionic species preferentially sorbing to the solid-phase.^79^ Yet, the fraction of HgCl^+^ formed at neutral pH and 0.5 M Cl^−^ concentration is only 0.006% (HgClOH, Hg(OH)_2_, and HgCl_2_ are expected to be the dominant species in solution based on these experiments).^79^ Hence, direct HgCl_2_ sorption to the solid-phase (and/or diffusion into the mineral matrix) may be the more dominant mechanism.

The most contaminated section of any soil core across both sites are the lower “artificial-filling” layers of redeposited soil material/building rubble in SCB1 (samples −1.25 to −3.25 m; Fig. 4). The pH of these layers is high, particularly between −1.75 m and −3.25 m (pH: 11.1 to 11.4; sample −1.25 m has pH of 8.7; Table S6.5†) and potentially associated with building materials such as concrete. Fe and Mn oxide formation can increase at higher pH.^76,81,82^ Thus, pH, the presence of Fe and Mn minerals and their greater stability in oxidised forms at the high pH of these lower artificial filling layers, and the reduced permeability of the underlying loess layer are likely important factors contributing to the elevated accumulation of Hg observed between −1.25 and −3.25 m in SCB1. There may also be some unknown sorption capacities associated with the building materials themselves that are anthropogenically derived materials (*e.g.* concrete) rather than natural minerals.

During initial contamination, the uptake process would have been kinetically controlled as an abundance of HgCl_2_ in solution sorbed (directly or indirectly) to available surfaces. As solid-phase sorption sites are filled, thermodynamics will become the dominant controller of Hg exchange between the solid- and liquid-phases. Both processes are expected to cause an increase in *δ*^202^Hg in the liquid-phase as lighter isotopes preferentially sorb to the solid-phase.^37,79,83^ Samples in the high concentration lower artificial filling layers in SCB1 (−1.25 to −3.25 m; *δ*^202^Hg: −0.17 ± 0.09‰ 1 SD) exhibit lower *δ*^202^Hg values compared to both the surface sample (−0.25 m; *δ*^202^Hg: 0.49‰) and the low permeability loess layer (−3.75 and −4.25 m; *δ*^202^Hg: 0.25 and 0.44‰, respectively) and the Rhine sediment layer down to the aquifer (−5.25 to −8.25 m; *δ*^202^Hg: 0.23 ± 0.10‰ 1 SD§§Data do not include −4.75 m sample at the top of sediment layer (*δ*^202^Hg: −1.01‰) with much lower THg (0.37 mg kg^−1^). This low THg, negative *δ*^202^Hg outlier sample is likely associated with heterogeneously distributed Hg.) (Fig. 4). The surface sample (−0.25 m) could be enriched in heavier isotopes due to (photo)reduction and evaporative losses.^84^ We relate the difference in *δ*^202^Hg between the high concentration artificial filling layers (more negative) and its underlying layers (more positive) to kinetic and/or equilibrium fractionation resulting in a preferential retention of lighter isotopes in the solid-phase of the lower artificial filling layers and enrichment of the liquid-phase Hg leaching into the underlying layers in heavier isotopes.

While THg concentrations are significantly lower in both the loess and Rhine sediment layers of SCB1 (*p* < 0.001), they are >2 mg kg^−1^ in all samples except the uppermost sample of the Rhine sediments (−4.75 m; see footnote§). Hence, the loess layer is of lower permeability, but not impermeable, allowing vertical Hg transport further down the soil core. Due to removal of lighter isotopes during sorption in the overlying artificial filling layers, we would expect the pool of liquid-phase HgCl_2_ slowly leaching through the loess layer to be enriched in heavier isotopes. This would result in a positive shift in *δ*^202^Hg of Hg sorbed to the solid-phase materials of the loess and Rhine sediment layers when compared to the artificial filling layers associated with sorption from this now heavier liquid-phase Hg pool, agreeing with the observed data.

Equilibrium isotope fractionation between Hg^0^ and Hg^2+^ results in more positive *δ*^202^Hg values in the Hg^2+^ pool,^83,85,86^ which could play a role in the observed Hg stable isotope data in SCB1. The *δ*^202^Hg value of the bulk sample will become more positive only if there has been Hg^0^ loss from the solid-phase (evaporation unfavourable in subsurface samples). Hg^0^ peaks were not observed in all samples of the SCB1 core and the largest fractions of Hg^0^ in SCB1 occurred at the bottom of the artificial filling layers (Fig. 4 and Section 3.2.3). For Hg^0^ production and loss to be responsible for the more positive *δ*^202^Hg in the loess and Rhine sediment layers we must assume: (i) substantial proportions of Hg^0^ must have been lost from the solid-phase of these layers (some with no evidence of solid-phase Hg^0^), and (ii) Hg^0^ losses in the artificial filling layers (in samples with both large and small Hg^0^ fractions) must have been small enough as to not impact the *δ*^202^Hg value in these layers. We deem this unlikely. Hence, we suggest the more negative *δ*^202^Hg values of the highly contaminated artificial filling layers are predominantly driven by fractionation associated with preferential sorption of lighter isotopes to the solid-phase.

##### 3.2.1.2 Is Hg^2+^ associated with mineral surfaces exchangeable?

In SEP analyses, highly soluble HgCl_2_ is typically associated with F1 (deionised water extraction), which is low and makes up 4.8 ± 2.7% in SCB1 samples (Fig. 4 and Table S7.3†). Moreover, this trend persists across all soil cores at both sites in samples considered contaminated: mean F1 fraction in samples with THg concentration > 1 mg kg^−1^ was 2.7 ± 3.4% (Section S7†). These SEP data on their own would suggest only a relatively small fraction of extractable labile Hg remains sorbed to the solid-phase samples. However, the sequential water extractions by Brocza *et al.*^55^ suggest a considerable amount of Hg exchangeable by water remains in the solid-phase after a single F1 extraction; and hence, not all Hg exchangeable by water is removed by the F1 extraction of SEP analyses. Both Bloom *et al.*^26^ and Fernández-Martínez and Rucandio^56^ also reported low recovery of HgCl_2_ and other soluble Hg species (based on the spiking of these species to soil samples) in F1 of their SEP analyses, although the later study used 0.2 M HNO_3_, which would be expected to improve the recovery of Hg exchangeable by water.

When we consider the more contaminated samples from cores across both sites (>1 mg kg^−1^), the mean fraction of THg extracted in F2 is 26 ± 22% and individual samples range up to 66% in this fraction (Section S7†). The 0.5 M HNO_3_ extraction is likely to remove a large fraction of the remaining solid-phase bound Hg exchangeable by water, but we cannot confirm complete removal is achieved in F2 without laboratory examination (*i.e.*, repeated/sequential F2 extractions), which should be assessed in future studies. Considering the industrial source is HgCl_2_ in solution and the incomplete extraction of all extractable labile Hg in F1, this limitation makes it very difficult to distinguish F1 from F2. The difference between the amount of Hg exchangeable by water released in F1 and F2 is likely associated with the strength of sorption or complexation with mineral surfaces and potential re-sorption during or after the F1 extraction step. For the studied systems, F1 is better described as *Hg easily exchangeable by water* and F2 as *Hg less extractable by water plus weakly complexed species (i.e., Hg associated with carbonates), and potentially HgO and HgSO*_*4*_.^26,56,57^

#### 3.2.2 Changes in Hg^2+^ speciation

While Section 3.2.1 suggests sorption of Hg exchangeable by water (*i.e.*, HgCl_2_) to mineral surfaces (or diffusion into the mineral matrix) in the solid-phase, particularly at site B, speciation analyses provide unequivocal evidence that a portion of the solid-phase Hg has been transformed into less mobile or residual forms of Hg^2+^. The mean fractions of F3 and F4 from SEP analyses across all solid-phase samples with >1 mg kg^−1^ THg concentration are 26 ± 14% and 49 ± 19%, respectively. F3 is reported to extract species such as Hg^2+^ bound to humic acids, Hg_2_Cl_2_, methyl-Hg (MeHg), and Hg^2+^ species more tightly bound to Fe- and Mn-oxides are also suggested to be released in this step.^26,57^ Aqua regia (F4) is required to dissolve the most tightly-bound Hg species such as α-HgS and β-HgS.

**Fig. 5:**
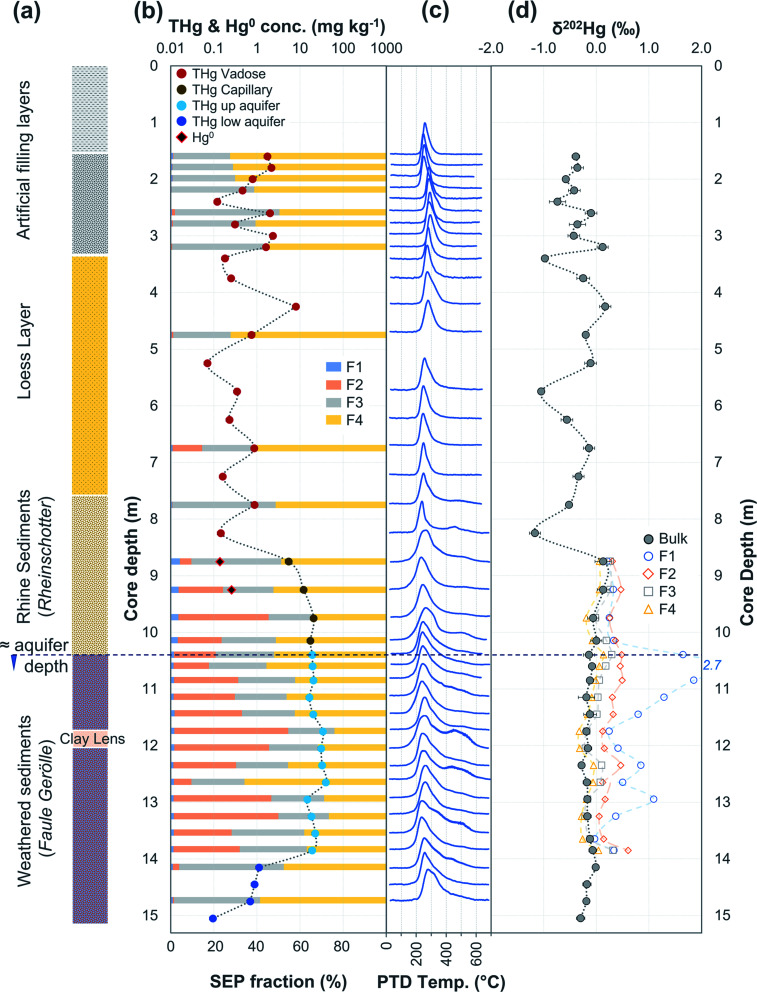
Multi-analyses summation of SCB2 solid-phase material. (a) Description of approximate soil core layer profile is based on our own visual characterisation and literature descriptions.^53–55^ (b) THg and Hg^0^ concentration (logarithmic scale) and SEP speciation data. (c) PTD speciation data – all signal attenuations are normalised to the maximum attenuation for each sample. (d) *δ*^202^Hg (MDF) stable isotope data. Excavation for pump and treat prevented sampling at top of core.

**Fig. 6:**
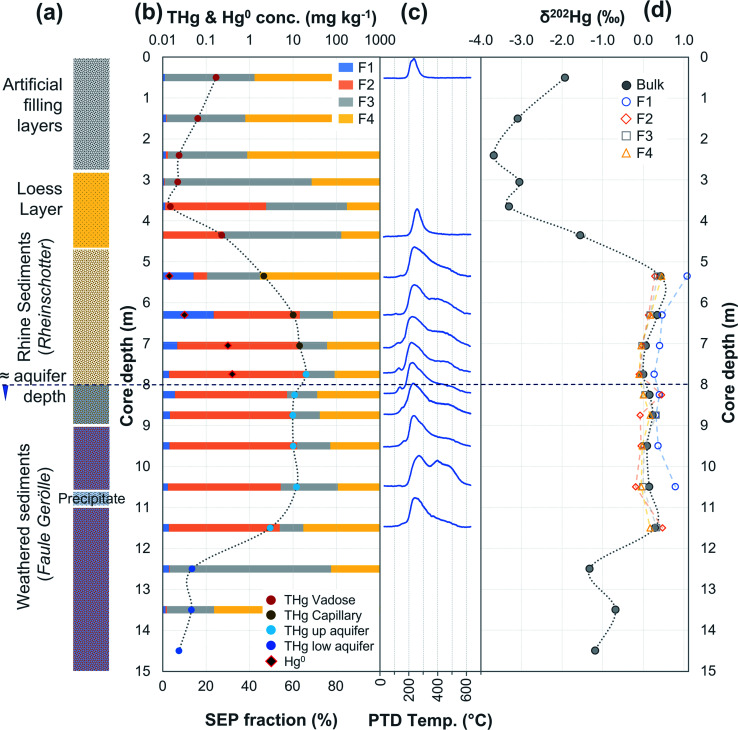
Multi-analyses summation of SCB3 solid-phase material. (a) Description of approximate soil core layer profile is based on our own visual characterisation and literature descriptions.^53–55^ (b) THg and Hg^0^ concentration (logarithmic scale) and SEP speciation data. (c) PTD speciation data – all signal attenuations are normalised to the maximum attenuation for each sample. (d) *δ*^202^Hg (MDF) stable isotope data.

In PTD analyses, there is a range of moderate temperatures (175–300 °C based on our data and instrumental setup) in which the release curves of a number of Hg^2+^ species reach their maximums; Biester and Scholz^25^ refer to this as “matrix-bound Hg”. All samples analysed by PTD have at least one clearly defined peak within this range. However, the PTD peak fitting analyses indicate wider and oddly shaped matrix-bound Hg peaks (multiple peaks within this range; Section S5†) or a matrix-bound Hg peak that is shifted from the lower end of the range (175–220 °C; HgCl_2_ release range) to the upper end of this range (250–300 °C) in some samples (*e.g.*, confined aquifer samples from SCA2 and SCA3) (Fig. 2–6 and Section S5†). Additionally, there are samples at both sites with a “shoulder” peak > 300 °C within all soil cores (*e.g.*, aquifer samples from SCA2, SCB2 and SCB3) (Fig. 2–6 and Section S5†). Matrix-bound Hg peaks closer to 300 °C and shoulder peaks > 300 °C indicate a change in chemical speciation from the original HgCl_2_ contamination to species produced *in situ* such as associations with pyrite, HgSO_4_, and *meta*-cinnabar (β-HgS) species (Fig. 1; see also Biester and Scholz^25^). There can be matrix effects (changes in mineralogy within a single core, between cores, or between sites) in PTD analyses that can lead to some variability in the release curves of certain species.^31,32^ However, we observe a weak, but significant positive relationship (*p* = 0.008; *R*^2^ = 0.129) between the F3 fraction (more residual species) and the temperature of the maximum signal attenuation between the temperature range for matrix-bound Hg peaks (175–300 °C) based on all data from the contaminated soil core samples (>1 mg kg^−1^; Fig. S7.1†). This gives us confidence that shifts in the matrix-bound Hg peaks between 175 to 300 °C are not merely associated with analytical imprecision and peaks closer to 300 °C are likely to be representative of an increase in the fraction of non-HgCl_2_ Hg species formed in the soil column present in the sample. Nonetheless, this does not completely rule out matrix effects in the PTD analyses, which may still contribute some uncertainty to the exact release temperatures of different species in the PTD analyses.

**Fig. 7:**
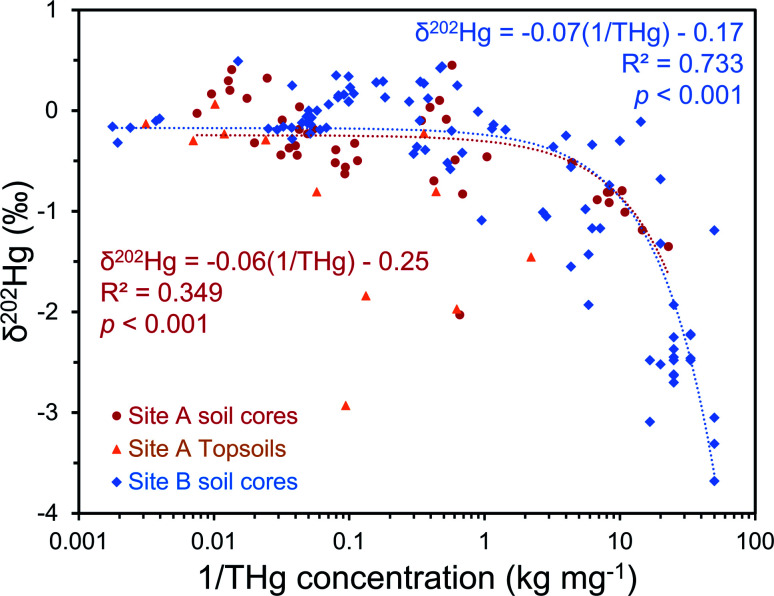
Multi-dimensional source appropriation analyses using *δ*^202^Hg values and the reciprocal of THg concentrations from solid-phase samples for both site A and site B. Site A topsoils were not included in source appropriation modelling at the site due to surface related processes that cannot occur to soil core samples; however, the model was (weakly) significant even if they were include with site A soil core samples (*p* = 0.010). Please note, these are linear mixing models on a log-based *x*-axis; linear-fitted models are expected in plots of delta values *vs.* the inverse concentration.^71^

##### 3.2.2.1 Speciation changes in capillary fringe and upper aquifer associated with changes in groundwater depth

There were some distinct features between SEP and PTD results specifically pertaining to increases in F2 fractions (SEP) correlating with the emergence of peaks or shoulder peaks at temperatures > 300 °C (PTD). This was apparent in the upper aquifer and/or capillary fringe at both sites (−7.5 m in SCA1; −5.25 to −7.75 m in SCB1; −9.75 to −13.85 m in SCB2; −6.5 to −11.5 m in SCB3; Fig. 2–6). These changes may be associated with Hg^2+^ species exchangeable by water or weak acid that have a higher intramolecular bond decomposition temperature. Haberer *et al.*^87^ discuss the impact of fluctuating water table on oxygenation and redox conditions, particularly in the aquifer capillary fringe. We know that the water table at site A is shallow and highly variable and at site B the water table depth during the study period was historically low due to an extended dry period before and during the sampling campaign. Hg speciation is more dynamic under variable soil–groundwater conditions (*i.e.*, water table depth, redox, oxygenation).^19,20^ Metastable sulphides (*i.e.*, pyrite) to which Hg is associated can react to form soluble or ionic oxidised species, such as HgSO_4_, under aerobic conditions.^88,89^

##### 3.2.2.2 Hg stable isotope analyses of SEP extracts: quantitative or qualitative?

SEP extracts were also analysed for Hg stable isotopes in many of the more contaminated sections of the soil cores. Generally, these results displayed a trend of fractions progressing towards lighter isotopes with increasing extraction strength; heaviest isotopes were found in the water-extractable fraction (F1) and lightest in the aqua regia fraction (F4) (Fig. 2–6 and Section S7†), which is consistent with the data from Brocza *et al.*^55^ However, other studies have reported different trends,^90,91^ or no significant isotopic variations between different soil extracts.^63^ These studies all used different SEP methods (different extractants), which will result in different species released in the extractions steps of these individual studies. This limits comparability between studies. Kinetic reactions will favour lighter isotopes in the product of an incomplete reaction.^34,35,37^ The process of partial transformation of sorbed Hg to more stable forms of Hg^2+^ found in the residual SEP fractions (F3/F4) is likely a stepwise process that, as we have suggested in Section 3.2.1, involves Hg^2+^ (*i.e.*, HgCl_2_) sorption to mineral surfaces initially followed by transformation to more residual species (*i.e.*, Hg-pyrite associations, HgS species, and potentially HgSO_4_ and other unknown species). Such a multi-step reaction process will drive further separation in *δ*^202^Hg values between the labile and residual fractions, which supports the observed data from the SEP stable isotope analyses. Thus, stable Hg isotope analyses of SEP extracted fractions provide a useful complementary tool that can confirm Hg transformations within environmental systems.

The relevant question to be asked is: can these SEP stable isotope results be used quantitatively or are they of a more qualitative nature? One source of uncertainty for stable isotope analyses on SEP extracts may be the extraction of certain Hg^2+^ species in differing SEP fractions due to variable physicochemical properties and mineralogy of the solid-phase matrix that have been reported.^25,27,28^ When examining more residual Hg species using SEP analyses (F3 and F4), the pH of soils must be considered. The very high pH between −1.75 and −3.25 m in SCB1 (11.1 to 11.4; Table S6.5†) has already been discussed in relation to the potential increase in Hg^2+^ sorption to Fe and Mn oxides in this section of the soil profile. SEP results show 17 ± 6% of THg is extracted in the labile (F1/F2) fractions, 19 ± 6% in F3, and 64 ± 6% in F4. The weak acid extraction used in F2 may be buffered by the high soil pH in some of our samples, partially neutralising the extractant acid, and resulting in more Hg being extracted in the subsequent extraction steps (F3 and F4). However, we used a stronger F2 extractant (0.5 M HNO_3_) to specifically account for the high carbonate content in samples at these sites.^55^ Experiments examining pH changes of extractant solution confirmed F2 was largely unaffected by high pH solid-phase materials (see Section S2† for details).

These extraction steps are dividing substantial proportions of the THg in each sample and it is possible the extraction processes themselves are imparting some analytical artefact on the observed proportions of stable Hg isotopes. Although Brocza *et al.*^55^ suggest differences in Hg stable isotopes in extracts are process driven, they also discuss how site-specific factors (*i.e.*, Hg speciation, soil matrix, or original contamination speciation) may have an effect on the results. We suggest this may go further: changes in the physicochemical properties and/or mineralogy of the solid-phase, which can affect the composition of species extracted in specific SEP fractions,^25,27,28^ have the potential to impact the observed stable isotope signatures of each SEP fraction. Considering the heterogeneity of Hg within contaminated soils,^7,92^ the recoveries of the sum of SEP extracts against THg concentrations of the same sample (samples > 1 μg kg^−1^) are acceptable (97 ± 23%, *n* = 70; Section S7†). Nonetheless, based on all data with measured stable Hg isotopes in SEP extracts there is a small systematic bias towards heavier isotopes when comparing the *δ*^202^Hg values from the bulk sample to the sum of SEP extracts (normalised to extract Hg concentration; mean difference is *δ*^202^Hg +0.11 ± 0.26‰ in ΣSEP extracts).

**Fig. 8:**
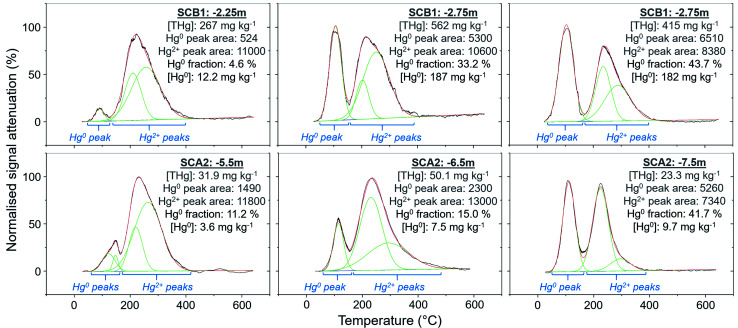
Peak fitting analyses (OriginPro) for soil samples with elevated Hg^0^ fractions or concentrations. Green lines show fitted Gaussian peaks, red lines indicate summation of peak heights, and the black line is the measured, normalised PTD signal attenuation. The Hg^0^ and Hg^2+^ peak areas are the (sum of) integrated area under the individual fitted peak(s) for these species as marked by the blue text and brackets in the figure. Hg_2_Cl_2_ could contribute a small fraction of the Hg^0^ peaks, although any uncertainty associated with this is likely small due to the relatively low stability of Hg_2_Cl_2_ under most environmental conditions.

The largest discrepancy in *δ*^202^Hg values between bulk and ΣSEP extracts occurs within the confined aquifer in SCA2 (*δ*^202^Hg in ΣSEP extracts: +0.44‰ at −6.5 m and +1.48‰ at −7.5 m). These two samples contain very large fractions of Hg^0^ as evidenced by PTD release peaks at ≈100 °C (Fig. 3 and 8). Due to its volatility, Hg^0^ is highly unstable and prone to evaporative losses during sampling and storage.^25,93^ Evaporative losses of Hg^0^ enriches the substrate in heavier isotopes.^70,94^ These particular SEP extractions were performed ≈18 months after sampling while the aqua regia digestions for THg and PTD analyses of SCA2 were performed within one month of sampling. SCB1 and SCB3 were again measured within one month of sampling for THg digests and PTD analyses, but SEP analyses were performed 4-5 months after sampling. It is highly likely that increased evaporative losses of Hg^0^ during the extended sample storage of SCA2 are responsible for the majority of the heavy isotope enrichment observed in these ΣSEP extracts. Removing these two samples in SCA2 (−6.5 and −7.5 m) improves the comparison between *δ*^202^Hg values from the bulk sample to the sum of SEP extracts (mean difference becomes *δ*^202^Hg +0.07 ± 0.15‰ in ΣSEP extracts) but does not completely remove this positive *δ*^202^Hg bias in the SEP extracts.

It has been demonstrated that Hg^0^ in solid-phase samples is not removed in a single extraction step but rather “smeared” across potentially all fractions.^25,26^ This issue becomes increasingly more problematic with increasing Hg^0^. It is notable that the *δ*^202^Hg values from all four SEP extracts in both samples (SCA2: −6.5 and −7.5 m) with large suspected loss of Hg^0^ during extended storage before analysis are shifted positive compared to the *δ*^202^Hg signature of the bulk sample (Fig. 3). This suggests Hg^0^ loss from all extraction steps and supports the notion that Hg^0^ is “smeared” across all extraction steps. Hence, there is potential for this “smearing” to artificially alter the ratios of Hg stable isotopes (*δ*^202^Hg value) in the extracts of each of the SEP extraction steps. Not being able to identify release of Hg^0^ in a single fraction also means *δ*^202^Hg changes associated with Hg^0^ production or equilibrium exchange between Hg^0^ and Hg^2+^ would not be observable in stable isotope analyses of SEP extracts. Considering these matrix (*i.e.*, changing solid-phase properties and mineralogy) and species-specific effects (*i.e.*, Hg^0^ smearing) on Hg stable isotope analyses of SEP extracts we view this as a complementary qualitative tool for Hg geochemical analyses that has potential to aid in the identification of *in situ* transformation processes rather than a means of quantitative assessment.

#### 3.2.3 Reduction of Hg^2+^ to Hg^0^

Considering that the initial Hg species used at the kyanisation facilities was HgCl_2_ and that there is no reported loss/spill of Hg^0^ at either site, the presence of Hg^0^ is almost certainly associated with the secondary reduction from Hg^2+^ in soils and/or groundwater. While it is not possible to identify Hg^0^ using SEP analyses due to the limitations associated with its extraction across multiple fractions, the identification of Hg^0^ is one of the strengths of PTD speciation analyses. Based on standards (Fig. 1), Hg^0^ release peaks occur between ≈100 and 175 °C. Samples with Hg^0^ were observed in all soil cores, in the vadose zone (SCB1 and SCA1) and the aquifer and its capillary fringe (SCA1, SCA2, SCB1, SCB2, SCB3) (Fig. 2–6). Based on the qualitative liquid-phase Hg speciation analyses in the elevated THg concentration wells, Hg^0^ was also detected in the groundwater at site A (8 ± 6% of THg) and site B (6 ± 4%). These data support results in other studies that have also detected Hg^0^ in soil cores and groundwater samples at both site A^20,95^ and site B.^54,55,95^

##### 3.2.3.1 PTD analysis: an effective tool for solid-phase Hg^0^ identification

The Hg^0^ peaks in the PTD solid-phase analyses are distinct from Hg^2+^ species including HgCl_2_, which, according to our in-house standard material, starts releasing Hg at higher temperature and reaches its maximum release at ≈190 to 225 °C. As discussed in the methods, there is some overlap in release peaks between Hg^0^ and Hg_2_Cl_2_ (Fig. 1). However, this species is not common due to its narrow range of conditions within which it is stable in the environment and is generally reported at trace levels if present.^77,96,97^ pH is a factor in this stability (Hg_2_Cl_2_ more stable at lower pH in a Hg contaminated system dominated by Cl associations)^98^ and the high pH of the lower artificial filling layer in SCB1 (pH > 11) that has elevated Hg release peaks < 175 °C make it very unlikely to occur at least in this location. While the low environmental stability of Hg_2_Cl_2_ does not favour its presence at these sites, we cannot categorically confirm or deny this with our presented data. Hg^0^ is much more stable under the conditions observed at these sites^20^ and has been commonly measured in untreated, purged groundwater samples at these sites (our study and others^20,54^). Thus, we assume Hg^0^ is responsible for the peaks < 175 °C in our samples but deem these analyses semi-quantitative due to possible minor inputs from Hg_2_Cl_2_ in this temperature range.

Peak identification and integration analyses were applied to estimate the fraction of Hg^0^ (out of THg), and from this, the Hg^0^ concentration in samples where it is present. The observed Hg^0^ fraction (in THg) ranged from <1 up to 41.7% in SCA2 −7.5 m and 43.7% in SCB1 −3.25 m (Fig. 8 and Section S5†). Elevated Hg^0^ and THg concentrations, especially in SCB1 −2.75 and −3.25 m, but also SCA2 −6.5 and −7.5 m, result in very high Hg^0^ concentrations up to 187 mg kg^−1^ (Fig. 8 and Section S5†). These results confirm PTD analyses as an effective tool for identification of Hg^0^ in solid-phase samples.

##### 3.2.3.2 Does Hg^0^ production enrich the solid-phase in Hg?

There is also the potential for Hg^0^ formation to contribute to Hg enrichment in areas where it occurs. Hg^0^ is poorly soluble compared to HgCl_2_ and when formed will not be readily leached. Thus, there could be Hg enrichment at a site of elevated Hg^0^ production if there is a continuous influx of Hg^2+^ from upper layers. SCB1 samples −2.75 and −3.25 m had large Hg^0^ fractions as determined by the PTD analyses (Fig. 4 and 8). However, all samples between −1.25 m and −3.25 m in SCB1 were heavily contaminated with Hg, but only the aforementioned samples (and −2.25 m that had a much lower Hg^0^ fraction) had detectable Hg^0^ fractions in the PTD analyses. Similarly, samples −5.5 to −7.5 m in SCA2 had elevated Hg^0^ concentrations, but these samples were not enriched compared to the samples above them in the soil profile. It is likely that Hg^2+^ initially sorbing to mineral surfaces (or diffusing into the mineral matrices) plays a more critical role in the Hg enrichment in this layer.

##### 3.2.3.3 Mechanisms of Hg^0^ production

One mechanism attributed to Hg^0^ production is the photochemically driven reduction of Hg^2+^ to Hg^0^.^99–101^ However, this process requires the input of solar radiation and cannot be responsible for the subterranean Hg^0^ production observed at these sites. Other possible processes include microbially mediated^18,96,100^ and abiotic^19,20,102^ reduction of Hg^2+^. The latter pathway is likely of greatest interest for our study sites given microbial activity and diversity can be compromised by elevated Hg (and also lead and arsenic; see Section S9†) concentrations.^103–105^

Abiotic reduction of Hg^2+^ can be facilitated by electron transfer from organic matter or most other less precious metals. The oxidation of Fe^2+^ or mixed valence (Fe^2+^/Fe^3+^) iron oxide minerals such as magnetite,^102,106^ siderite,^107^ vivianite,^108^ green rust,^109^ and clay minerals^110^ are likely to drive much of this reduction due to their relatively high abundance. This process can be expedited under anoxic or suboxic conditions within which such ferrous iron containing minerals become important electron donors.^19,20,111^ The fluctuating water table depth, oxygenation, and redox condition of these sites, particularly site A, are likely to have played a significant role in Hg^0^ production in the capillary fringe and upper aquifer.

Wiatrowski *et al.*^102^ and Amirbahman *et al.*^112^ demonstrated the importance of pH in this process; Hg^2+^ reduction is enhanced at higher pH. There were five samples with >10% Hg^0^ fraction: SCA2: −5.5, −6.5, and −7.5 m and SCB1: −2.75, and −3.25 m; additionally, sample SCB1 −2.25 m (4.6% Hg^0^) had a high Hg^0^ concentration of 12.2 mg kg^−1^ (Fig. 8). Based on the observed groundwater depth data from WA6a, we estimate these samples in SCA2 (−5.5, −6.5, and −7.5 m) to be at the top of the confined aquifer and pH values of the solid-phase materials were 7.40 ± 0.02. In samples directly below these (between −8.5 and −14.5 m), the pH dropped to 5.91 ± 0.02 (Table S6.2†). Similarly, the samples from SCB1 were from the lower artificial filling layer that had a very high pH of 11.3 ± 0.1, while the pH of the loess layer and Rhine sediments below this layer dropped to mean values of ≈7.9 and 6.8 ± 0.2, respectively (Table S6.5†). Thus, the elevated pH in these samples may be contributing to the increased production of Hg^0^ in these parts of the soil–groundwater systems. Given the low solubility and high volatility of Hg^0^ (Henry's law constant: 2.3 × 10^−8^ Pa^−1^ at 298 K (ref. 113), it can be expected that there will be Hg^0^ outgassing from contaminated aquifers containing Hg^0^ into soil cavities.

#### 3.2.4 Leaching, transport, and retardation processes in the aquifer

As discussed in Section 3.1.1, the highest liquid-phase THg concentrations at both sites were found further along the plume pathways rather than at the most adjacent wells to the surface contamination entry points (Table S3.1†). This is decidedly more evident at site B due to the more uniform topography and groundwater flow direction; as such, we will focus mainly on site B in this section.

##### 3.2.4.1 Hg leaching down the soil profile and identification of aquifer entry point

The two deeper samples (−2.75 and −3.25 m) in the lower artificial filling layer of SCB1 were ≈2× higher in THg than the upper two samples in this layer (−1.75 and −2.25 m). Additionally, ICP-OES analyses revealed elevated concentrations of other trace metals including zinc, lead, cadmium, and chromium at the bottom (samples −2.75 and −3.25 m) of this lower artificial filling layer (Section S9†). These data are indicative of intermittent “ponding” occurring above the low permeability loess layer during rainfall/snowmelt events that likely creates a temporary perched aquifer. These ponding events have the potential to produce more reducing conditions and represent a potential explanation for the Hg^0^ enrichment in these samples immediately above the poorly permeable loess layer. While a small fraction of the surface contamination has penetrated the loess layer into the Rhine sediments (Section 3.2.1), the THg concentrations in solid-phase aquifer material at this location (SCB1 below −8.25 m) are very low in Hg (0.05 ± 0.04 mg kg^−1^) and enriched in lighter isotopes (*δ*^202^Hg: −2.3 ± 0.4‰). This reflects the geogenic background signal rather than the less negative values of the industrial source found in the highly contaminated samples. As posited by Bollen *et al.*,^54^ the primary entry point of Hg to the aquifer cannot be at the site of SCB1.

Hg^2+^A (dissolved inorganic Hg^2+^) fraction dominated the liquid-phase speciation analyses (68 ± 14%) across all sampled wells at site B (and site A: 71 ± 21%). Based on the range of pH and redox conditions (Section S3†) and original Hg usage, we consider HgCl_2_ to be the dominant Hg^2+^A species as has been reported previously.^54^ These data also support the inference that SOM plays only a minor role in solid-phase sorption. If SOM played a more integral role in sorption, we would expect Hg^2+^B (DOM-bound Hg^2+^) to dominate liquid-phase speciation.

During the “ponding” events, HgCl_2_ in solution would likely have been transported away from the former kyanisation building down the slight gradient of the site above the loess layer.^54^ Groundwater THg concentrations in the well closest well to SCB1, WB3 (≈120 m along groundwater flow path from SCB1), are elevated (Table S3.1†). Hence, Hg must be able to (or at least in the recent past) leach into the aquifer between SCB1 and WB3. This could be the result of breaks in the loess layer, potentially caused by site construction, and/or thinning of the loess layer to the point where substantial Hg could leach through it.

##### 3.2.4.2 Groundwater Hg transport: why is the Hg groundwater plume not progressing?

The Hg groundwater plume extends ≈1.3 km along the groundwater flow path from the former kyanisation building. However, transport of the Hg groundwater plume at site B appears to have been hindered and potentially stopped completely. We did not observe any discernible increase in concentrations in wells at the end of the plume (WB17, WB23, WB24, or WB29) compared to previous work.^54^ Losses of Hg are likely associated with Hg^0^ degassing out of the groundwater, dilution with uncontaminated groundwater at the edges of the plume,^54^ and sorption to the solid-phase aquifer material along the plume. Hg groundwater plume retardation may also be linked to lower THg solubility associated with Hg^2+^ reduction to less soluble Hg^0^.

**Fig. 9:**
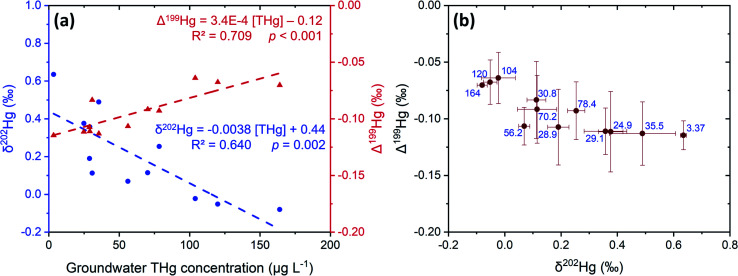
Comparison of mean Hg stable isotope values and THg concentrations in liquid-phase samples from site B groundwater across sampling events. Panel (a): relationships of *δ*^202^Hg (blue circles) and Δ^199^Hg (red triangles) against THg concentration. Panel (b): MIF (Δ^199^Hg) plotted against MDF (*δ*^202^Hg) with the blue text representing the mean THg concentrations (μg L^−1^). Error bars are 1 SD values for both MIF and MDF across the different sampling events. 2 SD analytical precision data are presented in Sections S2 and S3.†

The liquid-phase *δ*^202^Hg values are generally more positive – enriched in heavier isotopes – compared to the solid-phase samples at site B. Hg losses from the groundwater have resulted in a significant negative relationship between *δ*^202^Hg and THg concentration (Fig. 9). Essentially, soluble Hg (*i.e.*, HgCl_2_) transported away from the most contaminated part of the aquifer become progressively more enriched in heavier isotopes, supporting the notion posited in Section 3.2.1 that sorption from the liquid-phase to the solid-phase enriches the solid-phase in lighter isotopes. This fractionation to heavier isotopes should be enhanced when we consider that Hg^2+^ species are also enriched in heavier isotopes by equilibrium isotope fractionation between Hg^0^ and Hg^2+^.^85,86^ Thus, evaporative losses of Hg^0^ from the solid- or liquid-phase to soil airspaces or even groundwater well shafts would result in further enrichment of remaining solid and liquid-phase pools in heavy isotopes.

**Fig. 10:**
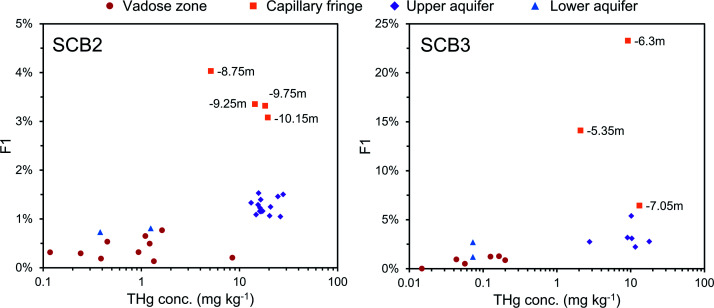
Proportion (%) of F1 in THg concentrations of the solid-phase samples from SCB2 (left panel) and SCB3 (right panel). THg concentration here are the sum of the measured Hg concentrations in the individual extracts.

Bollen *et al.*^54^ observed maximum THg concentrations in the same wells, but concentrations were generally ≈2–3× higher than our data in those wells. In July 2018, a pump-and-treat facility was installed to remove Hg from the groundwater at the site. Groundwater concentrations decreased by 1.2 to 6.4× from sampling before the installation to sampling after. Additionally, the summers in both 2018 and 2019 were exceptional hot and dry (groundwater depth at lowest level in previous 17 years). The dry conditions and lower water table mean there was little-to-no exchange between the groundwater and parts up the soil profile that have previously been within the aquifer during the sampling campaign at site B. We describe this area (approximately 1.5–2 m above the water table) generally as the “capillary fringe”. This “capillary fringe” is a section of the soil profile in all three site B cores with elevated labile SEP fractions of solid-phase materials (Fig. 4–6). In both SCB2 and SCB3 both THg concentration and the proportion of THg made up by F1 of the aquifer capillary fringe are elevated and group away from samples from the vadose zone and the aquifer proper (Fig. 10). The lower proportion of F1 within the aquifer is likely a result of this *Hg easily exchangeable by water* (*i.e.*, weakly sorbed HgCl_2_) being transported along the groundwater flow path (or transformed into a less soluble species). During historical periods of higher groundwater levels, the Hg groundwater plume would transport soluble Hg into the capillary fringe. Once the groundwater level decreases, a fraction of this soluble Hg would remain in the capillary fringe (as Hg easily exchangeable by water) and not be flushed away as continually occurs within the aquifer proper. We aim to examine these data in greater detail in a future experimental and modelling study addressing Hg transport in the aquifer at site B.

#### 3.2.5 Photochemical reduction and dark, abiotic redox equilibration: mass-independent fractionation (MIF) processes

As mentioned in Section 3.1, the variability of mean Δ^199^Hg values in both the liquid- and solid-phase samples is not large (Section S3 and S6,† respectively). Large MIF is typically associated with photochemical processes.^34,35,43^ Considering that Hg contamination at both sites is predominantly in subsurface soils, it is not surprising that variability in MIF is small. Potential for photochemistry is clearly greater in the surface soils; however, the mean Δ^199^Hg in the topsoil samples at site A were not significantly different from any of the soil cores (*p* = 0.266). Thus, photochemical processes are unlikely to play a major role in Hg cycling and the observed MIF signatures at the sites.

The most noticeable Δ^199^Hg MIF across the systems is the significant positive shift when comparing liquid-phase samples to solid-phase samples at either site A (Δ^199^Hg shift: +0.05‰; *p* < 0.001) or site B (Δ^199^Hg shift: +0.06‰; *p* < 0.001). Zheng *et al.*^44^ describe the potential for a negative Δ^199^Hg MIF shift in the Hg^2+^ pool during Hg(0)–Hg(ii) equilibrium fractionation after dark, anoxic Hg^0^ oxidation in the presence of thiol-groups associated with SOM and moisture. Small MIF shifts in the opposite direction to the MDF shift are expected for any process influenced by the nuclear volume effect, such as equilibrium isotope fractionation between co-existing Hg(0) and Hg(ii). Recent studies revealed that the exchange kinetics of this process are surprisingly fast,^86,114^ suggesting that equilibrium Hg isotope fractionation between Hg redox states can be expected to be relevant in all natural systems in which Hg(0) and Hg(ii) co-exist and get in contact with each other.

Our study and others^20,54,55,95^ have already demonstrated that Hg^0^ is present across both sites (Section 3.2.3), which provides the necessary starting point for this dark abiotic MIF process to occur. The oxygen content of the site A aquifer was generally low and variable across the sampling campaigns and mean values of the wells ranged from <1–68% saturation (Table S3.4†). The oxygen content in the site B aquifer was higher (range: 51–100%), but regularly below saturation (Table S3.5†). There may have been areas within vadose zone and aquifer capillary fringe of site B in the past in which intermittently wetted, and more anaerobic conditions prevailed based on the historically variable groundwater depth (Section S3†). Thus, the dark abiotic equilibrium isotope fractionation between Hg(0) and Hg(ii) may be responsible for the more negative Δ^199^Hg values observed in the liquid-phase samples and the significant positive relationship (*p* < 0.001) between Δ^199^Hg values and THg concentrations in groundwater at site B (Fig. 9).

## 4 Conclusions

This study demonstrates the potential for more robust, holistic approaches to improve interpretation of Hg geochemistry at contaminated sites based on the use of multiple analytical methods and data analysis across multiple environmental media at multiple sites. The addition of Hg stable isotope analyses to this comprehensive study was a key factor in extending our knowledge beyond what was previously possible with speciation and concentration analyses alone. Hg stable isotope data demonstrated evidence for the sorption of soluble Hg^2+^ species in the liquid-phase to solid-phase mineral surfaces (or diffusion into mineral matrices) – likely to Fe and Mn oxides (enrichment of solid-phase materials in lighter isotopes), losses of Hg from the liquid-phase during groundwater transport (enrichment of groundwater in heavier isotopes further away from the source area) using MDF, and potentially dark, abiotic redox equilibration between Hg^0^ and Hg(ii) using the combined MDF and odd-MIF fingerprint characteristic for an equilibrium isotope effect influenced by the NVE.

While we were able to distinguish industrial contamination Hg (less negative *δ*^202^Hg) from background Hg (more negative *δ*^202^Hg) using Hg stable isotopes in general terms, a general assessment such as this can also be accomplished using THg concentrations. Hg stable isotope MDF end member mixing models are difficult to employ as the isotopic signature of original Hg source material is usually not known and that of Hg ore minerals not well constrained. *In situ* transformation processes can also impart MDF. Thus, we suggest using Hg stable isotopes to understand these *in situ* biogeochemical processes driving MDF (and MIF) to be the more impactful application of the method than Hg source tracing.

The speciation analyses (PTD and SEP) and Hg stable isotope analyses of SEP extracts were beneficial complementary tools in extending these interpretations. Combining PTD analyses with Hg stable isotopes provided evidence that layers of Hg enrichment could not be primarily a result of reduction of Hg^2+^ species exchangeable by water (*i.e.*, HgCl_2_) to Hg^0^. The more negative *δ*^202^Hg values observed in these enriched layers (in SCB1) were likely a result of preferential sorption of lighter isotopes of Hg^2+^ from the liquid-phase to the solid-phase rather than a fractionation caused by equilibrium isotope exchange between Hg^0^ and Hg^2+^. Moreover, performing Hg stable isotope analyses of data on SEP extracts also exhibited some unique information, for instance, a progressively more negative *δ*^202^Hg shift in SEP stable isotope analyses from F1 to F2 to F3 to F4 suggests multiple speciation changes as light isotopes are favoured in products.

It is well established that speciation analyses such as PTD and SEP have both strengths and weaknesses; our comprehensive assessment of all the methods used in this study confirmed many of these advantages and disadvantages. PTD is an effective tool for identifying the presence of Hg^0^ and estimating Hg^0^ concentrations (peak-fitting) in solid-phase samples. The major limitation of this method remains the identification of specific Hg^2+^ species. While SEP analyses can provide additional information on some Hg species, fractions, or pools of Hg, many limitations or artefacts are matrix related, which our data also confirm. This was compounded in our study by the original contamination being HgCl_2_ and the presence of Hg^0^ species, both of which are not extractable in a single SEP fraction (the former as evidenced by the continuous release of Hg exchangeable by water, *i.e.*, HgCl_2_, in repeated water extracts of the same sample). It is likely there will be site-to-site variability in the SEP fraction some species are released in. Thus, it is difficult to recommend the use of SEP for general Hg geochemical site assessments when considered on its own without a specific methodological objective in mind.

### 4.1 Where to go from here?

Despite the demonstrated advancements in Hg geochemical interpretations highlighted by the addition of Hg isotope analyses within such a comprehensive study, considerable uncertainties and distinct unknowns remain. System complexity (multiple groundwater bodies, complex terrain, and highly variable redox conditions) observed particularly at site A can limit our ability to identify processes. A major limiting factor remains our inability to identify many specific Hg^2+^ species in environmental solid or liquid-phase samples, a task that is not advanced by analyses of Hg isotopes. There may be additional undiscovered information that we can derive from PTD and SEP methods, but likely by applying them in a complementary manner to other methods and not as stand-alone analyses.

The application of Hg isotopes in Hg geochemistry is still relatively new and many processes that might occur in the natural environment have not been examined in laboratory conditions to determine fractionation factors. This puts a limit on how much we can presently interpret Hg isotope signatures of environmental samples. It is critical that laboratory examination of fractionation factors for MDF and MIF of known and potential biogeochemical processes continue to be pursed and then examined *via* holistic field studies such as this. Equally as important is the need to continue to assess these processes in field-based studies as we have done here to determine if they are indeed relevant (do occur) and can be identified under environmentally relevant conditions. We are still only beginning to explore the potential of using Hg stable isotope signatures to provide novel insights into Hg biogeochemistry.

## Author contributions

D. S. M. wrote the manuscript, constructed all figures, performed data analyses, contributed extensively to field sampling campaigns, and performed liquid-phase THg and Hg speciation analyses, PTD solid-phase analyses, and complementary solid-phase analysis (IC-TOC). L. S. contributed to manuscript reviews, contributed to figure designs, performed data analyses, contributed extensively to field sampling campaigns, performed liquid- and solid-phase THg and Hg stable isotope analyses, solid-phase SEP, SEP stable isotope analyses, and ICP-OES on solid-phase samples (including Fig. S9.1†). J. G. W. designed the project concept and contributed to manuscript reviews, field sampling, and liquid- and solid-phase THg and Hg stable isotope analyses, and solid-phase SEP and SEP stable isotope analyses. J. P. contributed to liquid-phase THg and Hg speciation analyses, PTD solid-phase analyses, and complementary solid-phase analysis (IC, TIC-TOC), data analyses, manuscript reviews and manuscript formatting. L. C. contributed to field analyses and the liquid-phase THg and Hg speciation analyses, PTD solid-phase analyses, and complementary solid-phase analysis (IC, TIC-TOC). S. M. K. contributed to project concept, manuscript reviews, and direction of the project and analyses. H. B. designed the project concept and contributed to manuscript reviews, field sampling, and the liquid-phase THg and Hg speciation analyses, PTD solid-phase analyses, and complementary solid-phase analysis (IC, TIC-TOC).

## Conflicts of interest

The authors declare there are no conflicts of interest associated with this work.

## Supplementary Material

EM-024-D1EM00368B-s001
