# Supplementary material for: Demystifying mercury geochemistry in contaminated soil–groundwater systems with complementary mercury stable isotope, concentration, and speciation analyses
Source: Environ Sci Process Impacts. 2022 Jan 4;24(9):1406–29. doi: 10.1039/d1em00368b (PMC9491299; doi:10.1039/d1em00368b)
Supplement: EM-024-D1EM00368B-s001 [file EM-024-D1EM00368B-s001.pdf]

Electronic Supplementary Material (ESI) for Environmental Science: Processes & Impacts.
This journal is © The Royal Society of Chemistry 2021

Supplementary Information for:

Demystifying mercury geochemistry in contaminated soil–groundwater systems with complementary mercury stable isotope, concentration, and speciation analyses.

D. S. McLagan, L. Schwab, J. G. Wiederhold, L. Chen, J. Pietrucha, S. M. Kraemer, and H. Biester

Contents

S1. Maps and description of the sampling sites. 2

S2. Quality Assurance and Quality Control (QA/QC) Section 4

S3. Groundwater depths, THg and species concentrations, and other groundwater parameters from sampled wells 8

S4. Meteorology: historical and study periods averages 15

S5. Pyrolytic thermal desorption (PTD) peak fitting analysis 16

S6. Solid-phase THg, pH, moisture content and Hg stable isotopes 25

S7. Sequential extraction procedure (SEP) data & SEP isotope data 31

S8. Total carbon, organic carbon, and inorganic carbon analyses of solid phase materials 38

S9. ICP-OES data from site B 39

References 43

S1. Maps and description of the sampling sites.

SITE A:

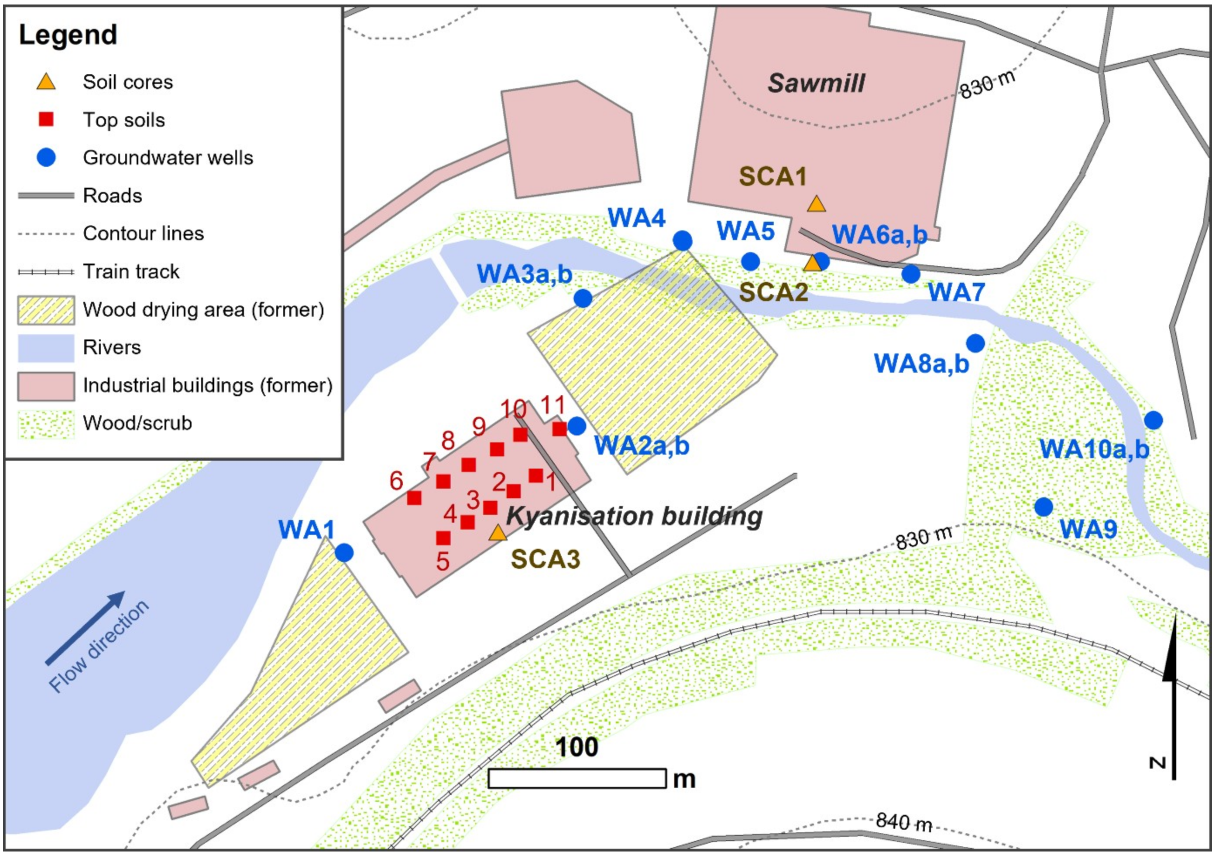

*Figure S1.1: Map of **site A** showing groundwater well, soil core, and topsoil sampling locations as well as the former industrial buildings and wood drying areas. Topsoil sampling locations 1 – 11 represent samples TSA1 – TSA11. TSA11 also had a subsoil sample (SSA11) taken at this location. Map produced in ArcGIS (ESRI).*

The site geology is made up of Palaeozoic granite underlying quaternary fluvial sediments that contain heterogeneously distributed organic materials derived from stratified peat lenses (Richard et al., 2016a; 2016b). This has resulted in a complex two-layer (confined and unconfined aquifers) groundwater system with highly variable flow direction across the site (Richard et al., 2016a). Groundwater depths for the wells at site A are presented in Section S3. SCA1 and SCA2 were drilled in October 2018 and SCA3 was drilled in July 2019. Topsoils were collected in October 2018. All soil core samples were the composite of 5-7 subsamples taken across the depth range of each sample and homogenised by mixing and shaking in polypropylene containers or double Whirl-Pak® bags. Measured samples were taken from this homogenised solid material. Topsoils followed the same procedure except that the 5-7 subsamples were taken from a 1 m^2 area around each sampling site.

SITE B:

In the 1970s, the area of the former industrial facility was converted into a residential zone and a 50 cm layer of uncontaminated material was added to the surface of the soils in this area after discovery of the extent of contamination in the 1990s (Schöndorf et al., 1999; Brocza et al., 2019). Under this layer is a 1 – 3 m thick layer of material artificially disturbed by industry and residential development made up of loess, loess/loam, and building rubble. A homogenous loess layer occurs until about 5 – 6 m and represents the top of the natural soils/layered sediments, which contain very low organic material (0.5 – 1%). Next is a layer of very low organic matter (<0.5 %) fluvial loose

gravel deposits we term "Rhine sediments". The unconfined aquifer below this material is highly permeable weathered sand-gravel sediments (flow velocity of 3 – 10 m d^{-1}; again, low in organic matter: <0.5 %). Finally, the aquitard is encountered between ≈13 – 16 m below the surface and is made up of low permeability weathered gravels (Schöndorf et al., 1999; Bollen et al., 2008). SCB1 and SCB2 were both drilled in December 2019, while SCB3 was drilled in May 2018. Soil core sampling at site B followed the methods outlined above for site A.

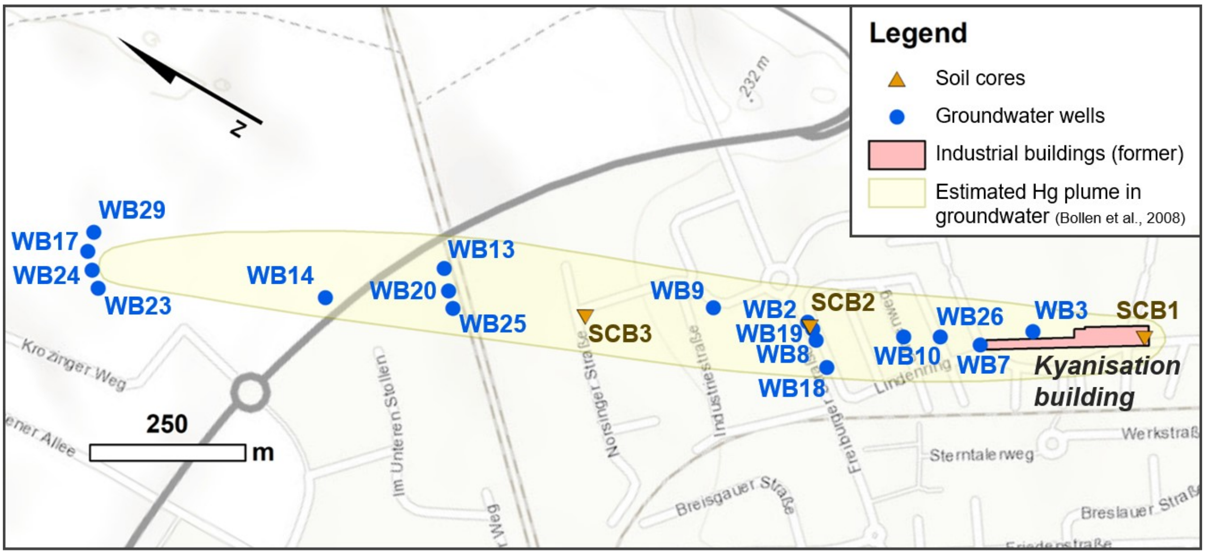

*Figure S1.2: Map of **site B** showing groundwater well and soil core sampling locations as well as the former industrial buildings. Map produced in ArcGIS (ESRI). The estimated groundwater plume is taken from Bollen et al. (2008).*

GROUNDWATER SAMPLING:

Groundwater was sampled five times at both sites: October 1 – 2, November 21 – 22, and December 11 – 12, 2018, and April 1 – 2 and July 29 – 30, 2019; and additionally, in September 2015 and May 2018 at Site B. Site B wells were always sampled on the first date of each sampling and Site A wells on the second. Not all wells were sampled during each sampling campaign due to (i) well lids frozen in place, (ii) no groundwater – dry wells, and (iii) repeated measurements below detection limits.

A "pump-and-treat" groundwater remediation conducted by an independent consulting firm has been on-going at site B since July 2018. Water was removed from wells WB2 and WB8, treated with activated carbon and Hg chelating scrubbers, and the groundwater scrubbed of Hg was reintroduced into well WB26. This scrubbed solution was also sampled and always below detection limits of the instrumentation. Samples from WB2, WB8, and WB26 were collected directly from taps within the pump-and-treat facility. WB2 was constructed at the same location as SCB2 after removal of the soil core material.

S2. Quality Assurance and Quality Control (QA/QC) Section

Total Hg Solid-Phase Analyses:

ERM-CC018 (contaminated sandy soil) was digested along with the solid-phase samples (*n* = 16) and the recovery was 92.9 ± 6.8 %. NIST-3133 was run throughout these analyses (n = 189), the average accuracy was 102 ± 4 % and RSD for individual session was between 0.8 – 6.2 %. Detection and quantification limits were 0.03 ± 0.04 and 0.10 ± 0.13 µg L^{-1}, respectively. Three ultrapure water field blanks were prepared on-site (one in each of the first three sampling campaigns) and transported back to the lab for analysis with the rest of the samples. All were below detection limits and therefore this was not continued during the later sampling periods as distilled water field blanks were also analysed for the liquid-phase Hg speciation analyses. 1 % BrCl solution was also analysed throughout the analytical sessions and was generally below detection limits (*n* > 100). Low concentration samples used for sequential extraction were additionally analysed in triplicate on a DMA-80 atomic absorption spectrometer (AAS, Milestone Srl). For quality control ERM CC-141 (loam soil) and NIST-3133 were measured along with the samples with recoveries of 92 ± 10 % (*n* = 5) and 97 ±4 % (*n* = 18), respectively.

Total Hg Liquid-Phase Analyses:

Quality control of these analyses was adjudged by repeated analyses of a 0.5 µg L^{-1} Hg calibration standard run as sample throughout these analyses (*n* = 35), the average accuracy was 102 ± 3 % and RSD for individual sessions was between 1.2 – 6.9 %.

Solid-Phase Hg Speciation Analyses – Pyrolytic Thermal Desorption (PTD):

Due to the need to generate sufficient signal peaks released during continuous temperature ramp of the sample, the detection limit of this method is ≈0.1 – 0.5 mg kg^{-1} THg; samples below this THg concentration were not considered. Irrespective of the area of individual peaks we can quantify the cumulative integrated area of Hg^{2+} peaks due to their distinct separation from Hg^0 peaks (Figure 1). While there is overlap between Hg^0 and Hg^{1+} species, calomel (Hg_2Cl_2), the latter is rare and generally believed to occur only at trace levels (if present at all) as a metastable intermediate phase prone to disproportionation (see Section 3.2.3 for details) (Schuster, 1991; Morel et al., 1998; Hazen et al., 2012). Therefore, Hg^0 peaks, Hg^0 fraction (of THg), and Hg^0 concentrations (calculated from this fraction and THg concentrations) can be quantitatively or at least semi-quantitatively assessed.

Solid-Phase Hg Speciation Analyses – Sequential Extraction Procedures (SEP):

The F3 1M KOH extraction step from Bloom et al. (2003) was omitted in our analyses due to the low OM content of the soils and suspected low sorption to the OM as suggested by Bollen et al. (2008). Test were conducted using the Bloom et al. (2003) extraction procedures on eight selected samples. Figure S2.1 shows the F3 1M KOH (organo-chelated Hg species) was small and <6.5% of THg in all samples except SCB2 -180cm. SCB2 -180cm is a relatively low THg concentration (2.16 mg kg^{-1}) with little surface Hg inputs except for some contamination associated with redistributed contaminated building materials. These data support the hypothesis that OM plays only a minor role in solid-phase Hg sorption at these sites.

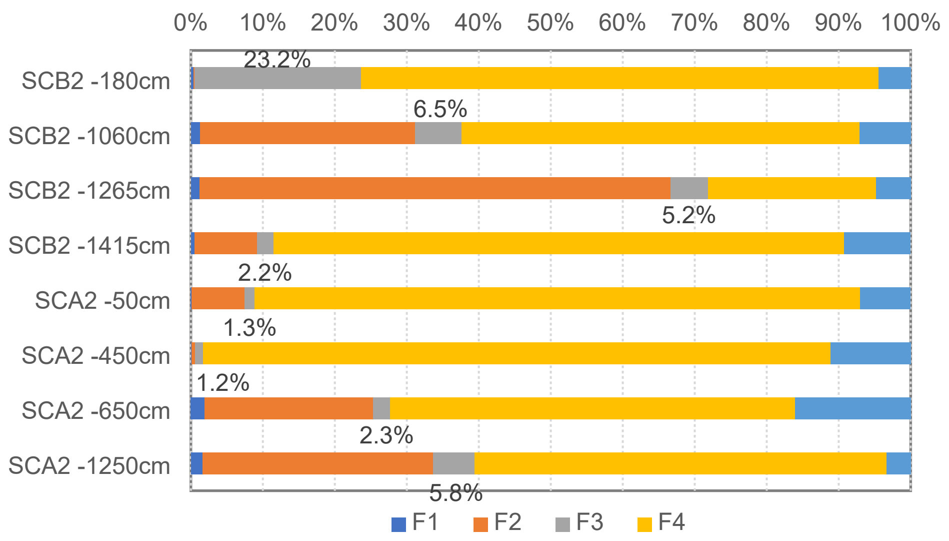

Figure S2.1: SEP results based on Bloom et al. (2003) method that highlight the minor role OM plays in solid-phase sorption at these sites. F3 (1M KOH) is typically associated with organo-chelated Hg species (Bloom et al., 2003); data from this fraction are labelled in the figure.

F2 extractant solution buffering by high pH solid-phase materials was also examined experimentally. The pH of the F2 extractant solution was 0.35 ± 0.03 (n = 4) and after addition of ≈1 g of sample from -2.75 m in SCB1 (pH = 11.1) and 20 h on an overhead shaker the pH rose only to 0.85 ± 0.03. This small change in pH is unlikely to have a significant effect on the species released in the F2 fraction. In contrast, previous extraction tests using the F2 reagent of the Bloom et al. (2003) method (0.01 M HCl / 0.1 M CH_3COOH) on carbonate-rich soil samples from site B resulted in a pH increase from 2.5 (reagent) to up to 5.7 after overnight extraction (Brocza et al., 2019), which is likely to decrease the extraction efficiency of the F2 step. Thus, the chosen F2 extractant may reduce some of the reported SEP limitations associated with changes in physicochemical properties and mineralogy.

It should be noted though that the solid-to-liquid-phase ratio of the Brocza et al. (2019) method applied in this study was 1:25 to increase Hg concentrations for stable isotope analyses of the individual extracts. This is lower than the recommended ratio (1:100) to ensure complete Hg extraction (Bloom et al., 2003). This issue is of greater concern for the labile fractions, F1 and F2 (Bloom et al., 2003), and may contribute to the incomplete extraction of Hg exchangeable with water and generally low combined proportion of Hg extracted in these labile fractions (16 ± 22 % based on all SEP analysed samples).

Liquid-Phase Hg Speciation Analyses:

Since three species/fractions, Hg^{2+}A, Hg^{2+}B, and Hg-part, are determined by difference and these calculated fractions include the uncertainty of multiple measurements, this can result in negative values for these fractions (Leopold et al., 2010; Richard et al., 2016b). Similar to Richard et al. (2016b), we allocate a criterion for valid samples as those >5 µg L^{-1} (quantification limits for THg by this method is ≈1 µg L^{-1}) and containing positive sample fractions, or a single negative fraction that is within 5 % of the expected higher fraction (the negative fraction is assumed as 0 % in this instance). Considering the high uncertainty of these speciation analyses, we deem them of a qualitative nature. Quality control for analysis by the Hg-254 Analyzer used a certified 115 µg L^{-1} Hg^{2+} in 5 % HNO_3 standard solution (Sigma Aldrich) and the recovery was 97 ± 6 % (n = 44). The distilled water field blanks were also analysed for Hg species and the concentration for all four measured species

was below the method detection limit for the Hg-254 Analyzer (detection limit: ≈0.3 µg L^{-1}) in all 13 distilled water field blanks across the sampling campaign.

Hg Stable Isotope Analyses:

A set of QC standards was run to assess the recoveries of Hg by the traps (BCR-482: 103 ± 12 %, *n* = 13; CC-141: 95 ± 4 %, *n* = 16; NIST-3133: 102 ± 4 %, *n* = 12). Assessment of accuracy and precision of individual measurement sessions is given in Table S2.1.

Table S2.1: Session averages and 2SD values for ETH Fluka secondary standard (continues onto next page). These data are comparable to other studies and a summary comparison can be found in Brocza et al. (2019): Table S6.

Session Date	Hg conc. [µg L^{-1}]	n	$\delta^{202}Hg$		$\Delta^{199}Hg$		$\Delta^{200}Hg$		$\Delta^{201}Hg$		$\Delta^{204}Hg$	
			Average [‰]	2SD [‰]	Average [‰]	2SD [‰]	Average [‰]	2SD [‰]	Average [‰]	2SD [‰]	Average [‰]	2SD [‰]
26.11.2018	10	**5**	**-1.47**	0.07	**0.09**	0.10	**0.04**	0.09	**0.03**	0.05	**-0.02**	0.03
28.11.2018	10	**3**	**-1.44**	0.07	**0.08**	0.03	**0.02**	0.09	**-0.03**	0.01	**0.06**	0.04
19.09.2018	10	**5**	**-1.44**	0.05	**0.09**	0.03	**0.01**	0.15	**0.03**	0.03	**0.00**	0.04
20.09.2018	10	**3**	**-1.43**	0.05	**0.08**	0.01	**0.01**	0.09	**0.02**	0.03	**0.00**	0.01
12.09.2018	2.5	**4**	**-1.47**	0.16	**0.09**	0.10	**0.23**	0.74	**0.05**	0.07	**0.00**	0.15
04.09.2018	10	**4**	**-1.35**	0.07	**0.09**	0.02	**-0.02**	0.06	**0.02**	0.05	**0.00**	0.04
05.09.2018	10	**7**	**-1.28**	0.08	**0.08**	0.03	**0.00**	0.08	**0.01**	0.07	**0.01**	0.04
20.08.2018	10	**4**	**-1.39**	0.05	**0.09**	0.02	**0.04**	0.08	**0.01**	0.02	**-0.02**	0.03
21.08.2018	5	**3**	**-1.38**	0.04	**0.07**	0.03	**0.03**	0.13	**0.03**	0.05	**0.00**	0.02
22.08.2018	5	**3**	**-1.35**	0.07	**0.08**	0.01	**-0.10**	0.35	**0.04**	0.05	**-0.02**	0.12
23.08.2018	10	**2**	**-1.43**	0.01	**0.09**	0.06	**0.11**	0.02	**0.03**	0.05	**-0.01**	0.08
10.01.2019	10	**3**	**-1.41**	0.07	**0.15**	0.24	**0.08**	0.15	**0.01**	0.03	**0.04**	0.05
24.01.2019	10	**4**	**-1.43**	0.09	**0.08**	0.06	**0.01**	0.10	**0.02**	0.04	**-0.02**	0.03
28.01.2019	10	**5**	**-1.38**	0.09	**0.07**	0.07	**0.04**	0.05	**0.02**	0.04	**-0.03**	0.05
31.01.2019	10	**5**	**-1.40**	0.13	**0.10**	0.03	**0.00**	0.14	**0.02**	0.03	**-0.02**	0.05
06.02.2019	10	**4**	**-1.35**	0.07	**0.05**	0.09	**0.01**	0.10	**0.01**	0.03	**-0.01**	0.06
07.02.2019	10	**5**	**-1.36**	0.07	**0.07**	0.04	**-0.01**	0.07	**0.02**	0.03	**-0.01**	0.04
28.02.2019	5	**4**	**-1.29**	0.11	**0.09**	0.06	**0.08**	0.15	**0.02**	0.07	**-0.06**	0.12
13.03.2019	10	**3**	**-1.43**	0.07	**0.07**	0.05	**0.01**	0.13	**0.02**	0.06	**-0.02**	0.05
14.03.2019	10	**6**	**-1.37**	0.09	**0.08**	0.04	**-0.03**	0.18	**0.01**	0.04	**-0.04**	0.05
18.03.2019	10	**4**	**-1.46**	0.04	**0.09**	0.04	**0.11**	0.13	**0.03**	0.02	**-0.02**	0.06
01.05.2019	10	**4**	**-1.38**	0.10	**0.09**	0.05	**0.03**	0.08	**0.04**	0.02	**-0.02**	0.02
02.05.2019	10	**4**	**-1.35**	0.15	**0.08**	0.03	**0.01**	0.06	**0.02**	0.04	**-0.01**	0.02
03.05.2019	3.5	**5**	**-1.38**	0.11	**0.08**	0.07	**0.11**	0.23	**0.02**	0.05	**0.04**	0.06
28.05.2019	10	**6**	**-1.38**	0.11	**0.07**	0.01	**0.03**	0.06	**0.03**	0.03	**0.01**	0.10
02.07.2019	10	**10**	**-1.38**	0.11	**0.07**	0.03	**0.03**	0.08	**0.03**	0.02	**0.01**	0.04
04.07.2019	10	**5**	**-1.41**	0.08	**0.09**	0.05	**0.04**	0.08	**0.03**	0.05	**-0.01**	0.10
04.07.2019	5	**5**	**-1.38**	0.05	**0.06**	0.04	**0.01**	0.28	**0.01**	0.04	**-0.02**	0.15
15.07.2019	2.5	**6**	**-1.42**	0.10	**0.09**	0.05	**0.05**	0.24	**0.05**	0.04	**-0.01**	0.14
07.08.2019	10	**5**	**-1.47**	0.08	**0.08**	0.01	**0.01**	0.04	**0.03**	0.01	**-0.02**	0.09
08.08.2019	5	**5**	**-1.42**	0.11	**0.09**	0.03	**0.03**	0.05	**0.02**	0.05	**-0.02**	0.05
08.08.2019	10	**5**	**-1.43**	0.07	**0.06**	0.04	**0.01**	0.03	**0.02**	0.04	**-0.02**	0.10
12.08.2019	10	**5**	**-1.45**	0.06	**0.07**	0.02	**0.03**	0.12	**0.03**	0.03	**-0.01**	0.08
17.10.2019	2.5	**6**	**-1.42**	0.15	**0.07**	0.07	**-0.02**	0.13	**0.05**	0.12	**-0.01**	0.12

Table S2.1 continued.

Session Date	Hg conc. [µg L^{-1}]	n	δ^{202}Hg Average [‰]	2SD [‰]	Δ^{199}Hg Average [‰]	2SD [‰]	Δ^{200}Hg Average [‰]	2SD [‰]	Δ^{201}Hg Average [‰]	2SD [‰]	Δ^{204}Hg Average [‰]	2SD [‰]
14.07.2020	10	**6**	**-1.44**	0.05	**0.08**	0.04	**0.02**	0.03	**0.01**	0.05	**-0.02**	0.05
15.07.2020	10	**7**	**-1.45**	0.06	**0.08**	0.03	**0.02**	0.02	**0.02**	0.03	**-0.01**	0.04
16.07.2020	5	**7**	**-1.45**	0.07	**0.08**	0.05	**0.02**	0.05	**0.03**	0.08	**-0.02**	0.08
23.07.2020	10	**6**	**-1.44**	0.12	**0.08**	0.02	**0.02**	0.02	**0.02**	0.03	**-0.03**	0.04
27.08.2020	10	**7**	**-1.46**	0.06	**0.08**	0.04	**0.01**	0.02	**0.01**	0.03	**-0.01**	0.06
28.08.2020	10	**7**	**-1.43**	0.05	**0.10**	0.03	**0.02**	0.07	**0.04**	0.07	**0.03**	0.10
05.08.2020	3	**5**	**-1.46**	0.10	**0.04**	0.07	**-0.02**	0.07	**0.02**	0.10	**0.02**	0.09
06.08.2020	3	**7**	**-1.44**	0.11	**0.09**	0.08	**0.03**	0.06	**0.03**	0.09	**0.00**	0.17
07.08.2020	3	**7**	**-1.44**	0.07	**0.11**	0.06	**0.04**	0.08	**0.05**	0.13	**-0.06**	0.14
08.08.2020	3	**5**	**-1.43**	0.07	**0.07**	0.06	**0.01**	0.08	**0.02**	0.08	**-0.05**	0.05
18.08.2020	3	**7**	**-1.42**	0.13	**0.07**	0.07	**0.01**	0.04	**-0.01**	0.04	**-0.01**	0.13
19.08.2020	3	**8**	**-1.41**	0.11	**0.08**	0.07	**0.02**	0.06	**0.03**	0.16	**0.00**	0.16
26.08.2020	3	**5**	**-1.38**	0.12	**0.07**	0.07	**0.03**	0.08	**0.03**	0.03	**-0.05**	0.05
27.08.2020	3	**6**	**-1.33**	0.12	**0.09**	0.07	**0.04**	0.05	**0.04**	0.05	**0.03**	0.10
30.09.2020	10	**5**	**-1.45**	0.07	**0.08**	0.04	**0.01**	0.01	**0.02**	0.02	**0.01**	0.06
02.10.2020	10	**6**	**-1.39**	0.08	**0.08**	0.04	**0.01**	0.03	**0.02**	0.06	**0.02**	0.06
06.10.2020	10	**7**	**-1.42**	0.05	**0.08**	0.02	**0.02**	0.02	**0.03**	0.05	**-0.01**	0.04
07.10.2020	10	**6**	**-1.44**	0.03	**0.08**	0.03	**0.02**	0.03	**0.03**	0.03	**-0.01**	0.02
13.10.2020	10	**6**	**-1.42**	0.07	**0.09**	0.02	**0.02**	0.02	**0.03**	0.06	**-0.03**	0.07
14.10.2020	10	**5**	**-1.43**	0.06	**0.08**	0.02	**0.03**	0.02	**0.02**	0.02	**0.00**	0.07
15.10.2020	5	**7**	**-1.43**	0.07	**0.08**	0.04	**0.03**	0.02	**0.03**	0.03	**-0.03**	0.11
16.10.2020	2.5	**9**	**-1.40**	0.15	**0.07**	0.09	**-0.01**	0.08	**0.02**	0.08	**0.00**	0.13
27.11.2020	10	**7**	**-1.48**	0.07	**0.08**	0.04	**0.02**	0.03	**0.03**	0.02	**-0.01**	0.08
29.11.2020	10	**5**	**-1.48**	0.06	**0.08**	0.04	**0.02**	0.04	**0.02**	0.03	**-0.02**	0.07
30.11.2020	5	**5**	**-1.48**	0.06	**0.08**	0.04	**0.02**	0.04	**0.02**	0.03	**-0.02**	0.07
01.12.2020	5	**5**	**-1.40**	0.06	**0.08**	0.06	**0.03**	0.07	**0.04**	0.06	**-0.03**	0.15
Overall	2.5 - 10	**329**	**-1.41**	0.12	**0.08**	0.06	**0.02**	0.14	**0.02**	0.06	**-0.01**	0.10

S3. Groundwater depths, THg and species concentrations, and other groundwater parameters from sampled wells

Liquid-phase pH, redox, conductivity, dissolved oxygen (DO) content, and temperature were measured on site with handheld probes during groundwater removal (Table S3.4 and Table S3.5). Groundwater depths were measured during each sampling using an electrical tape water level meter and are shown in Figure S3.1 and Figure S3.2.

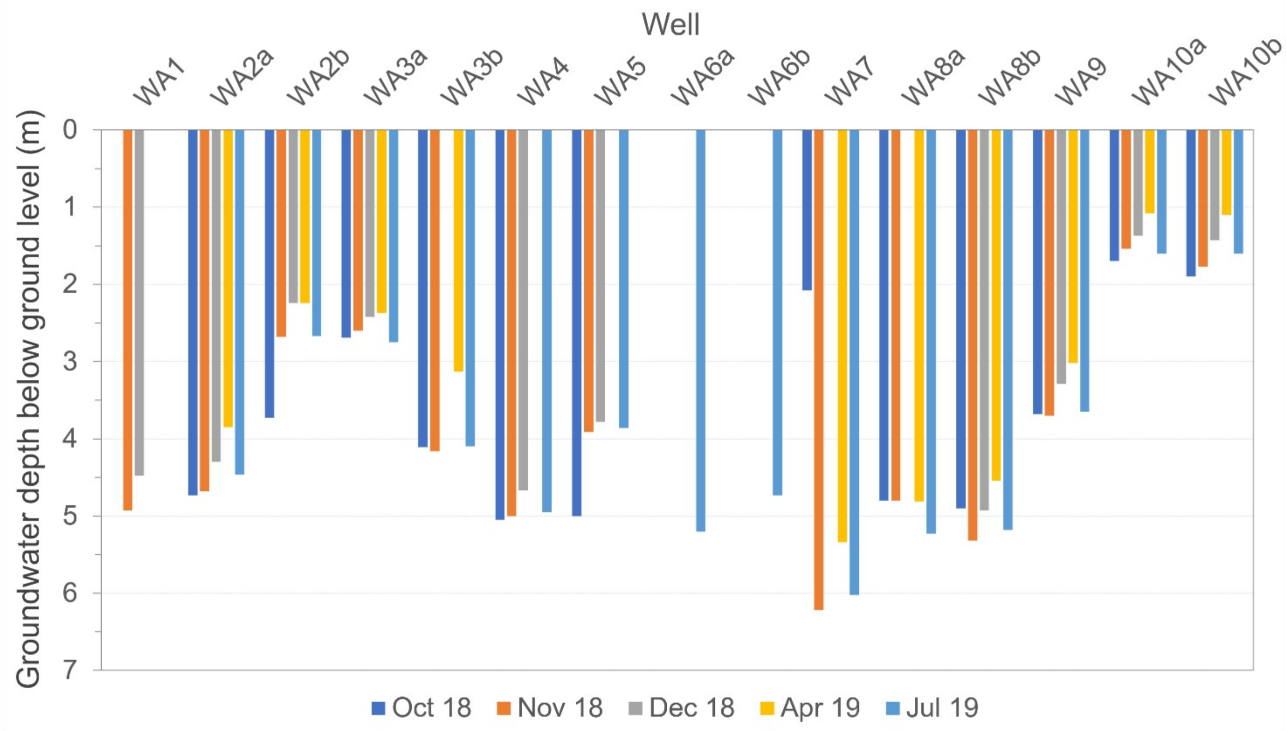

Figure S3.1: Groundwater depth below surface in sampled wells at site A across the sampling campaign.

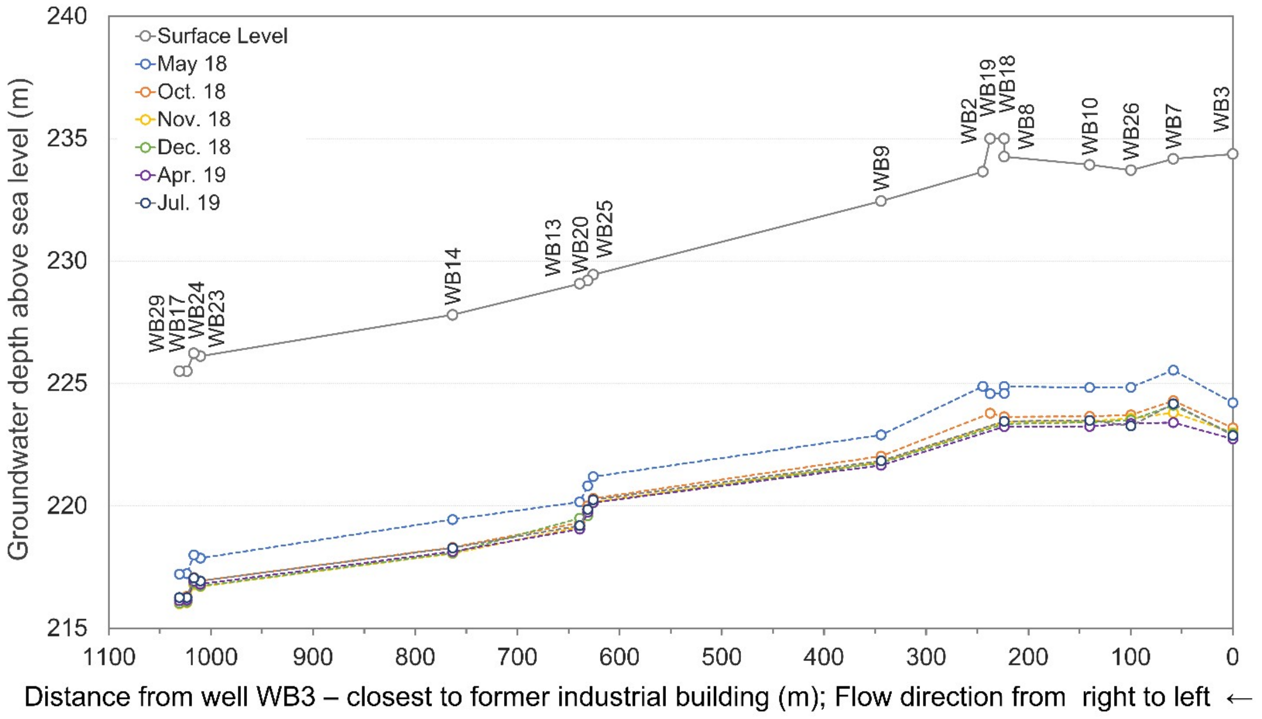

Figure S3.2: Groundwater depth above sea level (m) in sampled wells at site B by distance from well closest to source (WB3). Ground (or surface) level also indicated by grey line. These data reflect the very low groundwater depth after the May 2018 sampling campaign associated with the hot, dry conditions in 2018, 2019, and 2020 (see Figure S4.2).

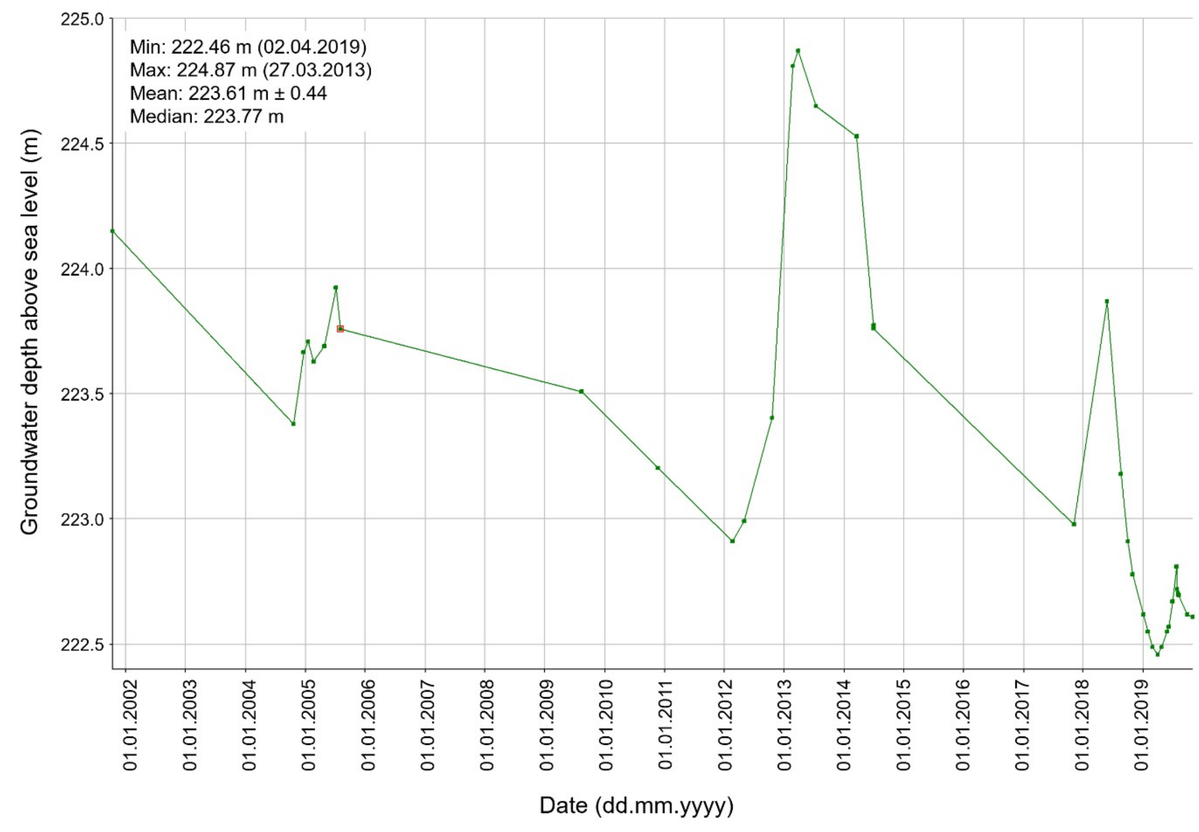

Figure S3.3: Historical groundwater depth at well WB18 from site B (Schöndorf, 2020). Data show the sampling period has the lowest groundwater depth for the previous 18 years.

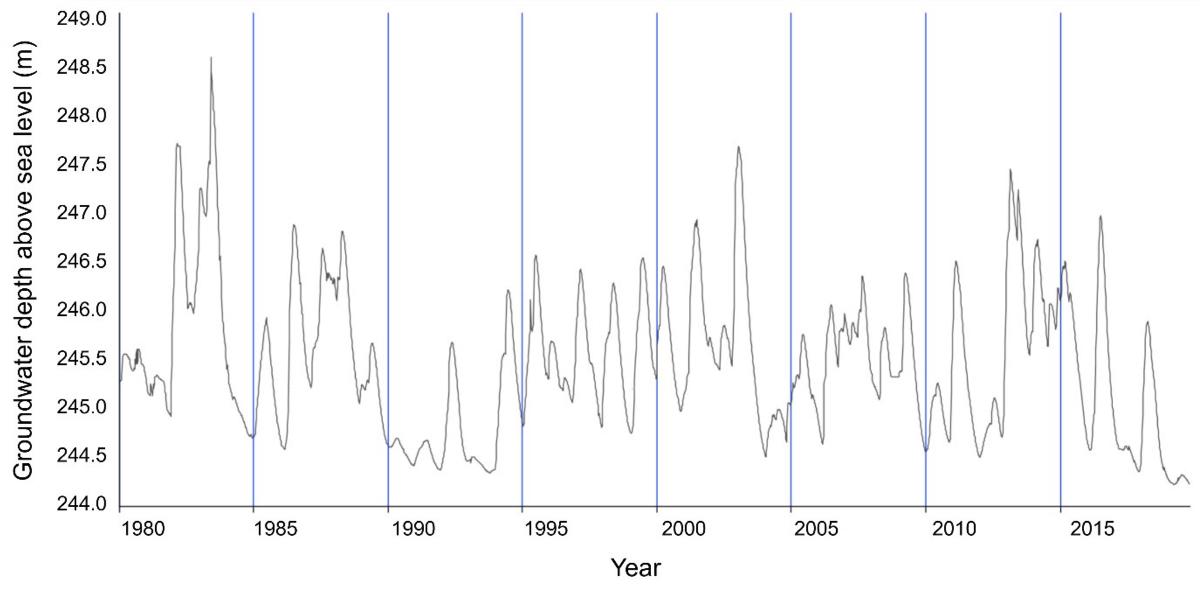

Figure S3.4: Historical groundwater depth at well 1600700 in Ehrenkirchen (Landesanstalt für Umwelt Baden-Württemberg, 2021). This is a long-term groundwater depth monitoring well and is ≈2.5 km from the former kyanisation building of site B. Data show the sampling period has the lowest groundwater depth for the previous 40 years.

Table S3.1: Mean and standard deviation (1SD, range of sample results) of total Hg (THg) concentrations, Hg stable isotopes, and Hg-speciation in groundwater from the different sampling events. Shading colours indicate THg concentration: blue (low: <1 µg L^{-1}), light blue (moderate: 1 < x < 10 µg L^{-1}), light red (high: 10 < x < 100 µg L^{-1}), red (very high: >100 µg L^{-1}). THg concentrations, Hg stable isotope (including 2SD analytical precision values), and Hg speciation data for each individual sampling event can be found in Table S3.2 and Table S3.3.

Site	Well	THg (µg L^{-1})	δ^{202}Hg (‰)	Δ^{199}Hg (‰)	Hg-part. (%)	Hg^{2+}A (%)	Hg^{2+}B (%)	Hg^0 (%)
Site A	WA1	0.01 ± 0.02						
	WA2b	0.49 ± 0.15						
	WA2a	1.89 ± 0.25						
	WA3a	0.22 ± 0.05						
	WA3b	0.06 ± 0.03						
	WA4	0.36 ± 0.25						
	WA5	2.94 ± 1.99						
	WA6a	14.3 ± 6.68						
	WA6b	3.77						
	WA7	288 ± 201	-0.23 ± 0.10	-0.06 ± 0.02	31.0 ± 14.1	53.3 ± 9.2	11.4 ± 9.8	4.3 ± 1.8
	WA9	0.01 ± 0.17						
	WA10a	298 ± 68.8	-0.17 ± 0.03	-0.08 ± 0.03	3.9 ± 5.2	86.8 ± 11.4	4.6 ± 6.8	4.7 ± 5.1
	WA10b	112 ± 68.2	-0.09 ± 0.04	-0.07 ± 0.03	2.2 ± 3.1	91.2 ± 3.9	4.1 ± 4.8	2.5 ± 0.6
	WA8a	1.21 ± 1.37						
	WA8b	18.0 ± 16.1	-0.08 ± 0.07	-0.06 ± 0.01	2.6 ± 4.2	53.1 ± 9.7	19.9 ± 14.2	17 ± 6.2
Site B	WB3	35.5 ± 38.2	0.49 ± 0.12	-0.11 ± 0.03	41.5 ± 32.2	35.3 ± 21.9	11.5 ± 11.3	11.7 ± 9.9
	WB7	28.9 ± 3.73	0.19 ± 0.04	-0.11 ± 0.03	20.4 ± 18.1	69.2 ± 14.2	6.1 ± 4.6	4.2 ± 2.5
	WB28	56.2 ± 10.9	0.07 ± 0.02	-0.11 ± 0.02	22.5 ± 14.6	68.1 ± 13.3	5.6 ± 6.7	3.8 ± 3.0
	WB10	120 ± 55.7	-0.05 ± 0.02	-0.07 ± 0.02	15.1 ± 13.6	67.3 ± 10.5	14.0 ± 20.3	3.5 ± 3.8
	WB8	104 ± 60.4	-0.02 ± 0.06	-0.06 ± 0.02	10.1 ± 6.2	82.4 ± 4.2	5.0 ± 5.4	2.6 ± 1.2
	WB18	1.28 ± 0.13						
	WB2	30.8 ± 5.37	0.11 ± 0.03	-0.08 ± 0.03	15.1 ± 8.1	69.2 ± 8.1	7.3 ± 10.4	8.5 ± 7.7
	WB19	164 ± 75.4	-0.08 ± 0.02	-0.07 ± 0.00				
	WB9	70.2 ± 24.0	0.11 ± 0.07	-0.09 ± 0.03	12.9 ± 9.9	82.2 ± 10.9	0.9 ± 1.8	4.0 ± 0.6
	WB20	78.4 ± 19.0	0.25 ± 0.03	-0.09 ± 0.03	5.4 ± 7.9	83.0 ± 7.8	8.7 ± 9.3	2.9 ± 2.7
	WB25	29.1 ± 7.56	0.36 ± 0.08	-0.11 ± 0.02	18.7 ± 8.4	65.0 ± 16.2	8.2 ± 4.4	8.1 ± 9.0
	WB13	24.9 ± 3.60	0.38 ± 0.04	-0.11 ± 0.04	12.2 ± 6.4	59.5 ± 26.7	15.3 ± 15.3	12.9 ± 15.0
	WB14	3.37 ± 2.43	0.63 ± 0.01	-0.11 ± 0.01				
	WB24	0.42 ± 0.08						
	WB17	0.26 ± 0.05						
	WB23	0.17 ± 0.04						
	WB29	0.18 ± 0.05						

Table S3.2: Liquid phase (groundwater) THg concentration, Hg stable isotopes and Hg speciation at ***<u>site A</u>****. The uncertainty values for δ^{202}Hg and Δ^{199}Hg are the 2SD values of repeated ETH Fluka measurements. Hg speciation fractions are defined as follows: Hg-part is Hg associated with particulate matter, Hg^{2+}A is dissolved inorganic Hg, Hg^{2+}B is dissolved organic Hg, and Hg^{0} is elemental Hg.*

Well	Oct. 2018						
	THg conc (µg L^{-1})	δ^{202}Hg (‰)	Δ^{199}Hg (‰)	Hg-part (%)	Hg^{2+}A (%)	Hg^{2+}B (%)	Hg^{0} (%)
WA5	5.7	-0.58 ± 0.11	0.09 ± 0.06				
WA7	78.0	-0.19 ± 0.07	-0.08 ± 0.10	13	67	13	7
WA3a	0.3						
WA3b	0.0						
WA4	0.7						
WA9	0.4						
WA10a	191.0	-0.19 ± 0.07	-0.08 ± 0.10	13	78	1	8
WA10b	181.6	-0.15 ± 0.07	-0.11 ± 0.03	4	93	0	2
WA8a	0.3						
WA8b	22.6	-0.13 ±	-0.06 ±	7	57	18	18
WA2b	0.4						
WA2a	2.0						
WA1							
WA6a							
WA6b							

Well	Nov. 2018						
	THg conc (µg L^{-1})	δ^{202}Hg (‰)	Δ^{199}Hg (‰)	Hg-part (%)	Hg^{2+}A (%)	Hg^{2+}B (%)	Hg^{0} (%)
WA5	2.4						
WA7	212.5	-0.31 ± 0.07	-0.05 ± 0.09	25	45	25	4
WA3a	0.2						
WA3b	0.0						
WA4	0.4						
WA9	0.0						
WA10a	297.9	-0.19 ± 0.13	-0.07 ± 0.03	3	94	3	1
WA10b	181.6	-0.08 ± 0.13	-0.04 ± 0.03	7	88	3	3
WA8a	0.6						
WA8b	29.9	0.00 ± 0.13	-0.07 ± 0.03	0	60	7	11
WA2b	0.8						
WA2a	2.0						
WA1	0.0						
WA6a							
WA6b							

Well	Dec. 2018						
	THg conc (µg L^{-1})	δ^{202}Hg (‰)	Δ^{199}Hg (‰)	Hg-part (%)	Hg^{2+}A (%)	Hg^{2+}B (%)	Hg^{0} (%)
WA5	2.7						
WA7							
WA3a	0.2						
WA3b							
WA4	0.1						
WA9	0.0						
WA10a	345.1	-0.13 ± 0.09	-0.13 ± 0.07	0	96	2	2
WA10b	47.1	-0.05 ± 0.13	-0.08 ± 0.03	0	93	5	2
WA8a							
WA8b	35.3	-0.11 ± 0.13	-0.12 ± 0.03	0	42	35	23
WA2b	0.5						
WA2a	1.5						
WA1	0.0						
WA6a							
WA6b							

Well	Apr. 2019						
	THg conc (µg L^{-1})	δ^{202}Hg (‰)	Δ^{199}Hg (‰)	Hg-part (%)	Hg^{2+}A (%)	Hg^{2+}B (%)	Hg^{0} (%)
WA5							
WA7	305.5	-0.26 ± 0.13	-0.04 ± 0.03	42	51	4	3
WA3a	0.2						
WA3b	0.1						
WA4							
WA9	0.0						
WA10a	285.0	-0.20 ± 0.15	-0.07 ± 0.03	4	95	1	1
WA10b	43.5	-0.08 ± 0.15	-0.05 ± 0.03	0	96	1	3
WA8a	3.2	-1.21 ± 0.09	0.12 ± 0.04				
WA8b	1.1						
WA2b	0.5						
WA2a	2.2						
WA1							
WA6a	19.04						
WA6b							

Well	Jul. 2019						
	THg conc (µg L^{-1})	δ^{202}Hg (‰)	Δ^{199}Hg (‰)	Hg-part (%)	Hg^{2+}A (%)	Hg^{2+}B (%)	Hg^{0} (%)
WA5	1.0						
WA7	555.3	-0.33 ± 0.07	-0.04 ± 0.04	42	51	4	3
WA3a	0.3						
WA3b	0.1						
WA4	0.3						
WA9	0.0						
WA10a	369.1	-0.18 ± 0.07	-0.06 ± 0.04	0	71	17	12
WA10b	108.1	-0.07 ± 0.07	-0.04 ± 0.04	0	86	12	2
WA8a	0.7						
WA8b	0.9						
WA2b	0.4						
WA2a	1.8						
WA1							
WA6a	9.59						
WA6b	3.77						

Table S3.3: Liquid phase (groundwater) THg concentration, Hg stable isotopes and Hg speciation at ***site B****. The 2SD values for the Hg stable isotopes analytical precision are the mean of 1SD analyses for each sampling period. No Hg speciation analyses were made on the May 2018 samples.*

	May 2018			Oct. 2018							Nov. 2018							Dec. 2018							Apr. 2019							Jul. 2019						
Well	THg conc (µg L^{-1})	δ^{202}Hg (‰)	Δ^{199}Hg (‰)	THg conc (µg L^{-1})	δ^{202}Hg (‰)	Δ^{199}Hg (‰)	Hg-part (%)	Hg^{2+}A (%)	Hg^{2+}B (%)	Hg^0 (%)	THg conc (µg L^{-1})	δ^{202}Hg (‰)	Δ^{199}Hg (‰)	Hg-part (%)	Hg^{2+}A (%)	Hg^{2+}B (%)	Hg^0 (%)	THg conc (µg L^{-1})	δ^{202}Hg (‰)	Δ^{199}Hg (‰)	Hg-part (%)	Hg^{2+}A (%)	Hg^{2+}B (%)	Hg^0 (%)	THg conc (µg L^{-1})	δ^{202}Hg (‰)	Δ^{199}Hg (‰)	Hg-part (%)	Hg^{2+}A (%)	Hg^{2+}B (%)	Hg^0 (%)	THg conc (µg L^{-1})	δ^{202}Hg (‰)	Δ^{199}Hg (‰)	Hg-part (%)	Hg^{2+}A (%)	Hg^{2+}B (%)	Hg^0 (%)
WB3	103.4	0.4 ±0.08	-0.12 ±0.05	26.6	0.47 ±0.08	-0.1 ±0.06	30	44	12	14	13.8	0.43 ±0.07	-0.13 ±0.24	28	59	6	7	10.9	0.44 ±0.08	-0.08 ±0.06					9.7	0.49 ±0.11	-0.11 ±0.05	89	8	1	2	8.7	0.44 ±0.08	-0.16 ±0.06	19	29	27	24
WB7	34.6	0.14 ±0.08	-0.07 ±0.05	26.6	0.22 ±0.08	-0.09 ±0.06	6	79	7	7	25.5	0.18 ±0.07	-0.16 ±0.24	25	74	0	1	32	0.25 ±0.08	-0.12 ±0.06	5	78	12	5	25.8	0.18 ±0.11	-0.10 ±0.05	49	45	4	3	29.1	0.18 ±0.08	-0.11 ±0.06	17	71	7	5
WB26	77.8	0.07 ±0.08	-0.11 ±0.05	53.5	0.07 ±0.08	-0.11 ±0.06	16	77	6	1	49.8	0.06 ±0.07	-0.08 ±0.24	47	48	0	5	54.6	0.07 ±0.08	-0.13 ±0.06	9	72	16	3	48	0.04 ±0.11	-0.10 ±0.05	17	81	0	2	53.3	0.11 ±0.08	-0.11 ±0.06	23	62	6	8
WB10	208.8	-0.04 ±0.08	-0.08 ±0.05	109.6	-0.01 ±0.08	-0.07 ±0.06	1	51	46	2	82.8	-0.08 ±0.07	-0.04 ±0.24	25	73	0	1	94.1	-0.05 ±0.08	-0.07 ±0.06	0	75	23	2	72.9	-0.06 ±0.11	-0.10 ±0.05	22	75	1	2	80.2	-0.05 ±0.08	-0.04 ±0.06	28	62	0	10
WB8	190.9	-0.09 ±0.08	-0.07 ±0.05	68.4	0.00 ±0.08	-0.09 ±0.06	10	83	5	3	61.8	0.00 ±0.07	-0.04 ±0.24	12	85	2	1	71	0.04 ±0.08	-0.09 ±0.06	3	80	14	3	62.5	-0.01 ±0.11	-0.07 ±0.05	7	87	4	2	78.4	0.02 ±0.08	-0.06 ±0.06	19	76	0	4
WB18	1.5			1.2							1.4							1.2							1.2							1.3						
WB2				37.1	0.09 ±0.08	-0.12 ±0.06	11	64	23	3	26.4	0.13 ±0.07	-0.05 ±0.24	25	71	0	4	34	0.08 ±0.08	-0.06 ±0.06	4	75	14	6	24.2	0.10 ±0.11	-0.13 ±0.05	15	78	0	7	32.2	0.16 ±0.08	-0.06 ±0.06	20	58	0	22
WB19	217.1	-0.07 ±0.08	-0.07 ±0.05	110.5	-0.09 ±0.08	-0.07 ±0.06																																
WB9	105	0.04 ±0.08	-0.13 ±0.05	62.5	0.13 ±0.08	-0.08 ±0.06					55.5	0.07 ±0.07	-0.04 ±0.24	4	92	0	4	55.4	0.16 ±0.08	-0.1 ±0.06	4	92	0	4	56.2	0.22 ±0.11	-0.09 ±0.05	23	74	0	3	51.6	0.15 ±0.08	-0.09 ±0.06	20	71	4	5
WB20	103.6	0.31 ±0.08	-0.12 ±0.05	74.8	0.25 ±0.08	-0.09 ±0.06	4	87	6	2	64.6	0.25 ±0.07	-0.05 ±0.24	0	80	18	1	69.5	0.27 ±0.08	-0.08 ±0.06	0	80	18	1	60.9	0.24 ±0.11	-0.08 ±0.05	4	94	0	2	68.3	0.23 ±0.08	-0.12 ±0.06	19	73	0	8
WB25	44.1	0.42 ±0.08	-0.11 ±0.05	27	0.48 ±0.08	-0.13 ±0.06					24.2	0.3 ±0.07	-0.11 ±0.24	17	78	4	2	24.3	0.35 ±0.08	-0.11 ±0.06	8	73	14	5	29.2	0.32 ±0.11	-0.07 ±0.05	23	67	6	4	26	0.27 ±0.08	-0.13 ±0.06	27	42	10	21
WB13	30.9	0.35 ±0.08	-0.15 ±0.05	27	0.46 ±0.08	-0.08 ±0.06	7	25	34	35	21.1	0.36 ±0.07	-0.12 ±0.24	20	79	0	1	22.7	0.34 ±0.08	-0.08 ±0.06					22.7	0.37 ±0.11	-0.09 ±0.05	7	82	6	5	25.4	0.36 ±0.08	-0.15 ±0.06	15	53	22	11
WB14	6.4	0.62 ±0.08	-0.11 ±0.05	6.5	0.64 ±0.08	-0.1 ±0.06					2.2																					1.5	0.64 ±0.08	-0.13 ±0.06				
WB24	0.4			0.5							0.4							0.5							0.5							0.3	0.23 ±0.08	-0.03 ±0.06				
WB17	0.3			0.3							0.2							0.3							0.3							0.2	-0.95 ±0.08	0.07 ±0.06				
WB23	0.2			0.2							0.2							0.2							0.2							0.1	-0.94 ±0.08	0.07 ±0.06				
WB29	0.1			0.2							0.2							0.2							0.2							0.1	-0.65 ±0.08	0.02 ±0.06				

*Table S3.4: Measured groundwater parameters for **site A** wells during the sampling campaign.*

Well	Temperature (°C)					Electro-conductivity (µS cm-1)					pH					Redox (mV)					Dissolved oxygen (saturation %)				
	Oct 18	Nov 18	Dec 18	Apr 19	Jul 19	Oct 18	Nov 18	Dec 18	Apr 19	Jul 19	Oct 18	Nov 18	Dec 18	Apr 19	Jul 19	Oct 18	Nov 18	Dec 18	Apr 19	Jul 19	Oct 18	Nov 18	Dec 18	Apr 19	Jul 19
WA1		9.3	9.4				183	182				6.7	6.1				197	216				14.4	0.53		
WA2a	10.7	11.7	11.3	10.4	10.4	592	446	473	448	460	6.0	6.6	5.8	5.8	6.1	267	198	215	213	238		28.8	12.4	5.4	9.7
WA2b		12.2	10.7	7.1	12.9		934	790	708	765		7.1	6.5	6.7	6.6		237	251	209	215		104	30.9	41.9	22.1
WA3a	13.4	12	10.8	7.4	11.7	414	360	355	303	322	6.8	7.1	6.4	6.7	6.8	264	190	199	218	172		34	0.4	0.8	0.1
WA3b	10.9	11.2		9.9	10.5	249	239		256	255	6.8	6.8		6.4	6.3	269	197		220	241		52.5		46.7	42.8
WA4	12.8	13.1	12.8		10.9		490	509		512	6.6	7.0	6.2		6.7	275	208	210		209		32.5	3.6		7.5
WA5	12.7	11.4	11.6		11.7		507	614		679	6.8	7.2	6.6		6.9	259	202	215		189		37.2	68.5		2.8
WA6a				8.8	14.4				492	594				6.6	6.8				197	220				67.8	41.5
WA6b					10.5					456					6.7					220					0.9
WA7	12	12		9.4	11	621	561		1983	767	6.8	7.0		6.7	6.6	275	207		204	237		36.3		49	44.4
WA8a	11.5	11.7		9.8	14.7	393	378		433	369	6.9			6.5	6.7	241	197		212	204		25.3		28.4	1.5
WA8b	10.1	10.2	10.8	10.4	10.2	495	456	444	484	484	6.2	6.5	5.8	5.9	6.1	265	196	209	205	238		36	3.2	10.5	7.2
WA9	10	9.7	9.1	8.1	7.5	358	330	329	344	94.6	6.4	6.5	5.7	5.9	5.8	253	198	213	205			61.7	37.4	39.8	41.6
WA10a	13.5	10.7	10.4	10	10.9	425	446	445	407	456	6.2	6.6	5.8	6.0	6.2	249	202	214	200	252		60.8	36.8	40.7	40.6
WA10b	11.5	11.4	10.4	10.3	10.7	423	414	406	371	397	6.2	6.6	5.9	6.0	6.3	227	198	206	202	251		77.5	30.1	22.3	27.6

*Table S3.5: Measured groundwater parameters for **site B** wells during the sampling campaign.*

Well	**Temperature (°C)**						**Electrical conductivity (µS cm^{-1})**						**pH**						**Redox (mV)**						**Dissolved oxygen (saturation %)**					
	May 18	Oct 18	Nov 18	Dec 18	Apr 19	Jul 19	May 18	Oct 18	Nov 18	Dec 18	Apr 19	Jul 19	May 18	Oct 18	Nov 18	Dec 18	Apr 19	Jul 19	May 18	Oct 18	Nov 18	Dec 18	Apr 19	Jul 19	May 18	Oct 18	Nov 18	Dec 18	Apr 19	Jul 19
WB3	13.1	12.5	12.2	12.3	12.8	13.1	428		356	341	339	340	6.5	6.7	7.3	6.6	6.5	6.6	134	258	217	177	221	208	87.9		90.5	68.7	69.6	72.5
WB7	13.0	12.6	12.2	12.2	12.7	14.2	443		368	352	350	353	6.2	6.7	6.9	6.6	6.5	6.5	173	248	213	188	216	211	75.6		80.1	66.9	68.0	72.2
WB26	13.0	12.7	12.2	12.4	12.9	15.3	441		362	346	341	344	6.2	6.7	6.8	6.6	6.5	6.6	143	280	218	188	218	229	78.5		83.0	69.0	69.6	74.9
WB10	13.2	12.8	12.4	12.4	13.0	13.6	461		366	349	340	343	6.2	6.7	6.8	6.6	6.5	6.6	183	288	222	199	213	244	86.0		100	67.9	70.0	71.1
WB8	13.7	12.6	12.3	11.8	13.4	13.3	453		357	140	351	337	6.3	6.7	7.0	6.6	6.6	7.1	180	263	247	265	226	206	81.7		76.4	74.1	79.3	88.1
WB18	13.5	12.9	12.7	12.6	13.4	13.9	443		373	357	355	365	6.4	6.7	6.8	6.5	6.4	6.8	184	262	222	205	218	207	81.2		100	65.7	65.0	63.1
WB2		12.8	12.3	11.5	12.9	13.8			356	380	334	345		6.7	6.9	6.6	6.5	7.0		274	259	276	232	154			83.0	72.6	80.0	86.2
WB19	13.2	12.8					459						6.2	6.6					212	288										
WB9	13.2	12.9	12.9	12.8	13.3	13.7	439		373	356	345	338	6.3	6.7	6.8	6.5	6.5	6.6	173	292	229	198	216	234	76.8		100	53.4	56.4	60.4
WB25	13.4	12.9	13.0	12.8	13.8	13.5	422		412	395	387	455	6.7	6.6	6.8	6.5	6.3	6.5	265	258	221	202	215	227	49.7		98.4	59.7	59.6	61.7
WB20	13.2	12.9	13.0	13.0	13.7	13.5	456		411	393	378	290	6.2	6.6	6.8	6.6	6.4	6.6	143	231	230	201	217	230	79.9		88.3	53.2	55.2	57.6
WB13	13.4	13.2	13.2	13.2	13.7	13.5	414		396	378	366	278	6.7	6.6	6.8	6.5	6.4	6.5	257	250	225	205	218	228	53.8		90.8	53.7	55.0	56.7
WB14	13.2	13.5	13.0	12.7	12.9	13.6	436		425	406	394	301	6.7	6.6	6.7	6.6	6.4	6.5	193	253	230	208	222	230	51.0		94.0	57.9	59.9	59.8
WB23	13.1	13.0	12.7	12.6	13.3	13.5	442		441	423	428	328	6.7	6.6	6.8	6.5	6.5	6.6	183	265	232	216	221	224	73.0		93.7	69.2	71.9	72.6
WB24	12.9	13.2	12.7	12.5	13.4	13.4	431		431	413	405	303	6.7	6.6	6.8	6.6	6.5	6.5	180	266	227	212	220	226	73.0		100	72.1	78.1	75.3
WB17	13.0	12.9	12.5	12.5	13.3	13.4	430		433	413	404	395	6.7	6.6	6.9	6.6	6.5	6.5	203	286	233	210	218	218	61.4		96.5	69.2	74.0	74.8
WB29	13.1	12.8	12.5	12.4	13.2	13.1	428		432	413	395	292	6.7	6.6	6.9	6.6	6.5	6.4	268	263	227	208	219	230	59.2		99.2	70.3	73.1	74.5

S4. Meteorology: historical and study periods averages

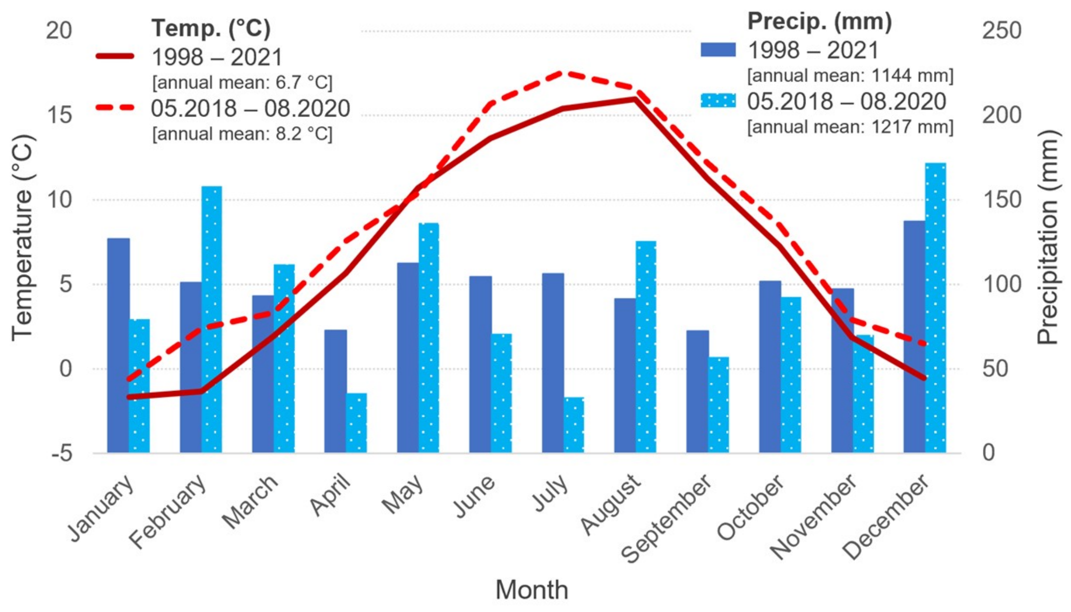

*Figure S4.1: Mean monthly temperature and precipitation for Lenzkirch weather station (47.8597 °N, 8.2308 °E). This station is ≈6 km from **site A**. Data taken from available temperature and precipitation data in Deutscher Wetterdienst (2021). Data during the sampling period are indicative to hotter, drier conditions compared to the mean conditions from 1998 – 2021.*

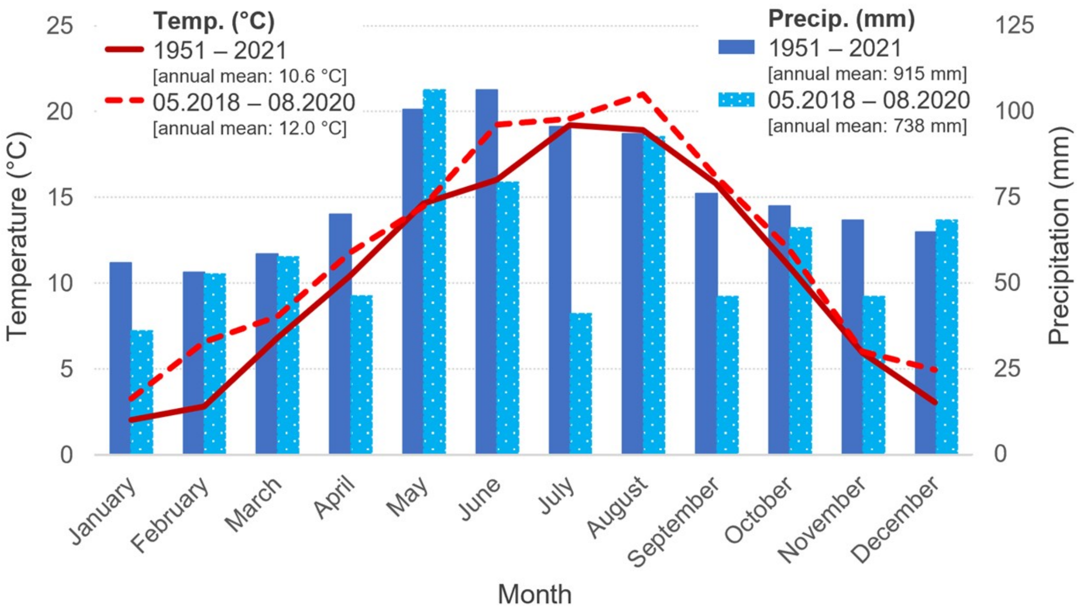

*Figure S4.2: Mean monthly temperature and precipitation for Freiburg im Breisgau weather station (48.0033 °N, 7.8558 °E). This station is ≈14 km from **site B**. Data taken from available temperature and precipitation data in Deutscher Wetterdienst (2021). Data during the sampling period are indicative to hotter, drier conditions compared to the mean conditions from 1951 – 2021. These conditions were linked to the low aquifer depth at the site.*

S5. Pyrolytic thermal desorption (PTD) peak fitting analysis

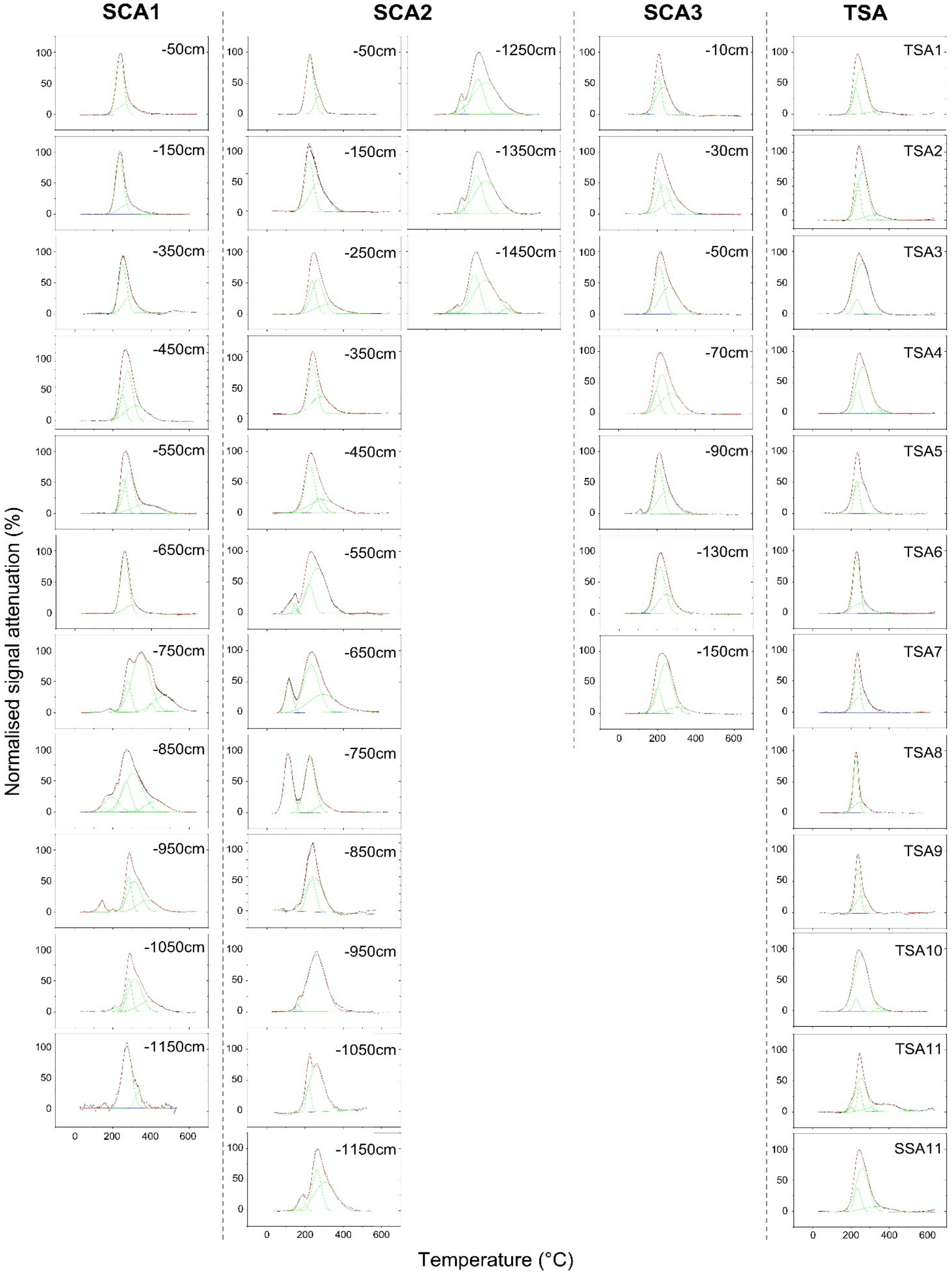

*Figure S5.1: PTD peak fitting analyses from **site A solid phase materials**. Peaks are fitted with Gaussian peak shapes shown by green lines. This adds uncertainty to quantification of Hg^{2+} species that we deem qualitative analyses. Integrated peak area data are presented in Tables S5.1 – S5.4. TSA refers to site A topsoils.*

Table S5.1: Area integration data from PTD peak fitting analyses for ***SCA1*** *from Figure S5.1.* Hg^{2+} *peaks have a maximum peak temperature of >175 °C,* Hg^0 *peaks <175 °C (red highlight). Quantitative data for* Hg^0 *fractions and concentrations are also included and calculated from sum of the integrated areas of all peaks.*

Sample		Max peak temp. (°C)	Max peak height	Peak Type	Integrated area	Proportion Hg^0	THg conc (mg kg^{-1})	Hg^0 conc (mg kg^{-1})
SCA1	-50cm	238.2	86.5	Gaussian	4354.1			
		276.7	18.1	Gaussian	2103.8			
	-150cm	235.7	91.1	Gaussian	4803.3			
		283.9	16.3	Gaussian	1884.5			
	-350cm	250.5	77.9	Gaussian	4141.6			
		287.8	27.4	Gaussian	2284.3			
	-450cm	249.1	32.3	Gaussian	1307.0			
		277.2	59.1	Gaussian	3888.4			
		320.9	18.2	Gaussian	2521.4			
	-550cm	255.7	54.8	Gaussian	2301.2			
		284.2	65.8	Gaussian	4418.0			
		372.6	15.1	Gaussian	2530.2			
	-650cm	259.1	94.1	Gaussian	5037.4			
		300.3	13.6	Gaussian	1320.3			
	-750cm	177.6	5.3	Gaussian	275.3			
		277.7	49.8	Gaussian	2310.8			
		345.7	93.1	Gaussian	10708.5			
		400.4	14.8	Gaussian	434.2			
		465.0	24.9	Gaussian	3431.7			
	-850cm	160.1	6.1	Gaussian	187.6	1.2%	12.6	0.1
		191.5	23.9	Gaussian	2379.0			
		266.6	49.7	Gaussian	3279.1			
		305.8	61.4	Gaussian	7685.2			
		425.1	17.5	Gaussian	2353.4			
	-950cm	141.3	16.5	Gaussian	674.9	7.1%	1.6	0.1
		197.4	4.1	Gaussian	99.6			
		282.4	56.2	Gaussian	2160.3			
		312.6	49.0	Gaussian	4277.4			
		379.3	18.8	Gaussian	2339.0			
	-1050cm	211.4	7.2	Gaussian	379.6			
		284.8	53.0	Gaussian	2022.3			
		315.5	52.4	Gaussian	4684.3			
		388.7	16.6	Gaussian	2246.9			
	-1150cm	272.6	82.4	Gaussian	5427.5			
		332.3	20.9	Gaussian	978.4			

Table S5.2: Area integration data from PTD peak fitting analyses for ***SCA2*** *from Figure S5.1. Hg^{2+} peaks have a maximum peak temperature of >175 °C, Hg^0 peaks <175 °C (red highlight). Quantitative data for Hg^0 fractions and concentrations are also included and calculated from sum of the integrated areas of all peaks.*

Sample		Max peak temp. (°C)	Max peak height	Peak Type	Integrated area	Proportion Hg^0	THg conc. (mg kg^{-1})	Hg^0 conc. (mg kg^{-1})
SCA2	-50cm	223.3	91.7	Gaussian	4636.4			
		269.6	28.1	Gaussian	1394.2			
	-150cm	220.0	66.6	Gaussian	3927.1			
		266.9	37.1	Gaussian	4072.3			
	-250cm	235.2	52.9	Gaussian	2675.5			
		264.3	54.6	Gaussian	4388.4			
		317.2	14.9	Gaussian	2279.7			
	-350cm	237.2	80.9	Gaussian	4782.2			
		280.5	27.8	Gaussian	3437.4			
	-450cm	224.1	76.6	Gaussian	5139.9			
		270.8	24.0	Gaussian	1645.4			
		293.3	21.2	Gaussian	3759.9			
	-550cm	122.7	18.7	Gaussian	1076.5	11.2%	31.9	3.6
		147.2	17.2	Gaussian	412.5			
		219.4	46.9	Gaussian	2856.5			
		263.8	72.8	Gaussian	8945.9			
	-650cm	114.6	50.0	Gaussian	2298.4	15.0%	50.1	7.5
		230.9	77.2	Gaussian	7728.9			
		293.1	28.6	Gaussian	5312.7			
	-750cm	111.3	95.5	Gaussian	5257.9	41.7%	23.3	9.7
		224.1	87.8	Gaussian	6204.2			
		295.3	12.8	Gaussian	1135.0			
	-850cm	231.7	41.1	Gaussian	2370.8			
		248.3	40.1	Gaussian	4418.6			
	-950cm	164.3	10.4	Gaussian	235.4	2.2%	1.4	0.0
		259.2	91.4	Gaussian	10818.6			
	-1050cm	211.4	7.2	Gaussian	379.6			
		284.8	53.0	Gaussian	2022.3			
		315.5	52.4	Gaussian	4684.3			
		388.7	16.6	Gaussian	2246.9			
	-1150cm	181.0	20.8	Gaussian	921.8			
		261.8	65.9	Gaussian	3905.9			
		304.4	45.9	Gaussian	6315.4			
	-1250cm	177.5	22.5	Gaussian	708.6			
		265.9	56.9	Gaussian	4414.9			
		308.3	51.0	Gaussian	8577.0			
	-1350cm	178.7	17.5	Gaussian	493.8			
		260.0	60.9	Gaussian	4796.8			
		308.3	52.6	Gaussian	7904.3			
	-1450cm	116.5	3.1	Gaussian	78.0	4.2%	0.7	0.0
		154.0	10.3	Gaussian	486.1			
		248.8	62.5	Gaussian	4573.4			
		303.7	53.7	Gaussian	7889.3			
		420.3	8.9	Gaussian	468.1			

Table S5.3: Area integration data from PTD peak fitting analyses for ***SCA3*** *from Figure S5.1.* *Hg^{2+} peaks have a maximum peak temperature of >175 °C, Hg^0 peaks <175 °C (red highlight). Quantitative data for Hg^0 fractions and concentrations are also included and calculated from sum of the integrated areas of all peaks.*

Sample		Max peak temp. (°C)	Max peak height	Peak Type	Integrated area	Proportion Hg^0	THg conc (mg kg^{-1})	Hg^0 conc (mg kg^{-1})
SCA3	-10cm	206.6	60.5	Gaussian	2503.6			
		233.0	44.0	Gaussian	4238.7			
	-30cm	204.3	56.8	Gaussian	2983.9			
		235.1	47.6	Gaussian	3674.1			
		285.2	23.5	Gaussian	3035.0			
	-50cm	213.1	75.8	Gaussian	4724.9			
		264.9	43.9	Gaussian	5283.2			
	-70cm	196.3	36.3	Gaussian	1720.8			
		226.9	63.9	Gaussian	4822.7			
		276.0	33.8	Gaussian	4370.9			
	-90cm	110.1	7.6	Gaussian	181.5	2.1%	78.2	1.7
		208.9	74.1	Gaussian	4283.8			
		249.0	37.4	Gaussian	4103.5			
	-110cm	213.3	67.7	Gaussian	3629.8			
		243.7	35.1	Gaussian	3866.5			
	-130cm	213.3	74.9	Gaussian	5287.2			
		248.0	30.8	Gaussian	3344.1			
	-150cm	199.1	41.8	Gaussian	2103.3			
		240.0	80.7	Gaussian	7230.7			
		300.6	9.7	Gaussian	1459.6			

Table S5.4: Area integration data from PTD peak fitting for ***site A topsoils*** *from Figure 5.1. Hg^{2+} have a maximum peak temperature of >175 °C. No Hg^0 peaks were detected likely due to surface evaporative losses.*

Sample		Max peak temp. (°C)	Max peak height	Peak Type	Integrated area	Proportion Hg^0	THg conc (mg kg^{-1})	Hg^0 conc (mg kg^{-1})
Site A topsoils (TSA)	TSA1	226.8	41.2	Gaussian	1576.3			
		252.5	73.0	Gaussian	5362.1			
		338.3	4.8	Gaussian	664.5			
	TSA2	235.6	43.7	Gaussian	1692.2			
		260.5	57.0	Gaussian	4072.8			
		323.7	6.3	Gaussian	794.5			
	TSA3	231.5	23.2	Gaussian	1077.0			
		256.5	81.1	Gaussian	8752.0			
	TSA4	232.8	36.2	Gaussian	1684.2			
		261.8	74.4	Gaussian	7042.4			
		357.1	5.1	Gaussian	354.9			
	TSA5	229.4	52.0	Gaussian	1878.8			
		249.4	50.8	Gaussian	4950.7			
	TSA6	228.7	85.9	Gaussian	3681.6			
		251.3	15.8	Gaussian	1793.4			
	TSA7	231.4	70.2	Gaussian	2775.3			
		257.1	29.9	Gaussian	2918.6			
	TSA8	235.2	71.8	Gaussian	2540.1			
		258.4	30.1	Gaussian	2341.7			
	TSA9	225.4	83.8	Gaussian	2740.6			
		247.2	16.1	Gaussian	1383.2			
	TSA10	227.6	19.9	Gaussian	769.8			
		253.6	89.2	Gaussian	8523.6			
		354.7	4.8	Gaussian	263.6			
	TSA11	200.3	7.2	Gaussian	215.5			
		244.0	39.5	Gaussian	1165.8			
		252.3	53.2	Gaussian	3850.6			
		368.4	12.1	Gaussian	1914.8			
	SSA11	234.0	37.5	Gaussian	1677.9			
		258.6	70.5	Gaussian	5980.1			
		332.6	8.0	Gaussian	1333.2			

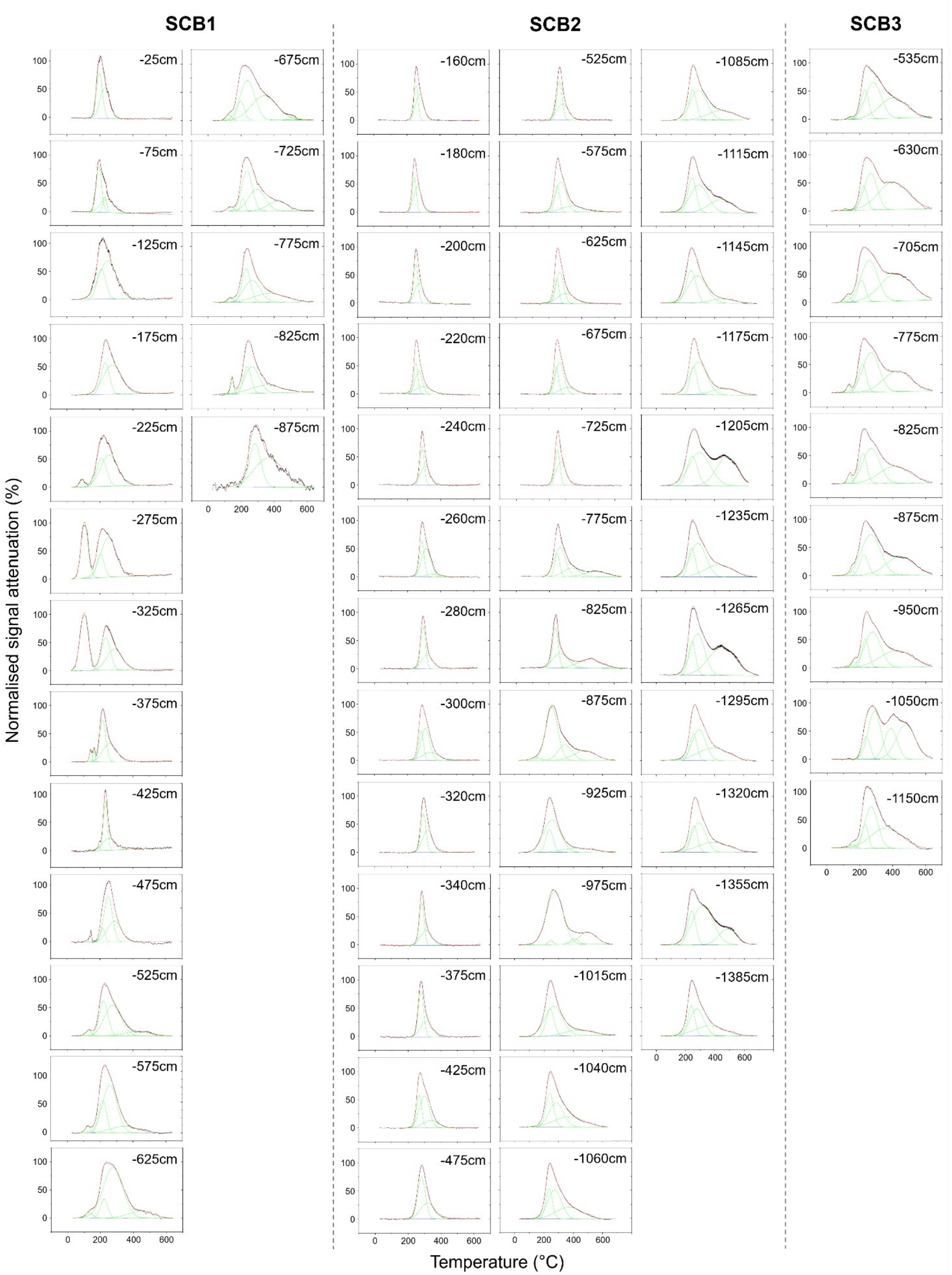

*Figure S5.2: PTD peak fitting analyses from **site B solid phase materials**. Peaks are fitted with Gaussian peak shapes shown by green lines. As discussed in the paper and evidenced by the peak shape of the in-house standard materials this adds uncertainty to quantification of Hg^{2+} species that we deem qualitative analyses. Integrated peak area data are presented in Tables S5.5 – S5.7.*

Table S5.5: Area integration data from PTD peak fitting analyses for ***SCB1*** *from Figure S5.2.* Hg^{2+} *peaks have a maximum peak temperature of >175 °C,* Hg^0 *peaks <175 °C (red highlight). Quantitative data for* Hg^0 *fractions and concentrations are also included and calculated from sum of the integrated areas of all peaks.*

Sample		Max peak temp. (°C)	Max peak height	Peak Type	Integrated area	Proportion Hg^0	THg conc. (mg kg^{-1})	Hg^0 conc. (mg kg^{-1})
SCB1	-25cm	197.2	67.2	Gaussian	3026.0			
		230.4	45.9	Gaussian	3246.4			
	-75cm	193.6	80.5	Gaussian	3348.5			
		229.1	27.7	Gaussian	817.5			
		243.4	23.3	Gaussian	2457.7			
	-125cm	208.4	44.5	Gaussian	2936.6			
		246.5	56.8	Gaussian	7630.2			
	-175cm	231.7	56.5	Gaussian	3579.4			
		275.3	51.7	Gaussian	7270.0			
	-225cm	90.3	13.2	Gaussian	524.5	4.6%	267	12.2
		209.9	50.1	Gaussian	3697.5			
		256.7	55.9	Gaussian	7286.2			
	-275cm	105.9	99.8	Gaussian	5304.3	33.3%	562	187
		202.5	41.5	Gaussian	2356.9			
		251.9	70.4	Gaussian	8279.7			
	-325cm	103.6	102.8	Gaussian	6510.3	43.7%	416	182
		235.7	57.9	Gaussian	3940.2			
		288.1	37.8	Gaussian	4443.0			
	-375cm	143.1	20.2	Gaussian	398.5	9.4%	26.4	2.5
		162.9	20.9	Gaussian	309.4			
		216.1	74.4	Gaussian	3428.3			
		258.0	32.4	Gaussian	3427.9			
	-425cm	195.9	6.4	Gaussian	69.2			
		233.3	72.7	Gaussian	2278.4			
		254.9	17.6	Gaussian	1843.6			
	-475cm	142.9	13.3	Gaussian	220.8	2.6%	0.4	0.0
		215.8	20.8	Gaussian	737.3			
		250.3	68.2	Gaussian	4352.2			
		287.9	30.8	Gaussian	3243.9			
	-525cm	129.8	9.8	Gaussian	423.8	3.3%	3.6	0.1
		221.9	61.6	Gaussian	4072.7			
		276.9	54.9	Gaussian	6904.6			
		427.3	7.9	Gaussian	1505.4			
	-575cm	124.5	8.1	Gaussian	345.2	3.0%	6.3	0.2
		215.2	42.2	Gaussian	2436.9			
		257.7	61.8	Gaussian	6930.7			
		346.3	8.5	Gaussian	1761.2			
	-625cm	133.0	8.4	Gaussian	445.3	2.5%	10.1	0.3
		220.9	33.7	Gaussian	1865.7			
		274.0	90.2	Gaussian	13321.2			
		441.8	12.3	Gaussian	2323.9			
	-675cm	133.0	8.4	Gaussian	445.3	2.5%	12.5	0.3
		220.9	33.7	Gaussian	1865.7			
		274.0	90.2	Gaussian	13321.2			
		441.8	12.3	Gaussian	2323.9			
	-725cm	133.4	7.2	Gaussian	373.2	2.6%	5.6	0.1
		209.2	22.4	Gaussian	774.3			
		294.4	38.7	Gaussian	4937.5			
		419.7	17.4	Gaussian	2673.9			
		235.1	71.1	Gaussian	5661.8			
	-775cm	136.3	6.4	Gaussian	327.3	2.6%	2.8	0.1
		230.9	59.8	Gaussian	3849.4			
		266.1	39.3	Gaussian	4781.7			
		372.1	15.4	Gaussian	3716.4			
	-825cm	144.4	26.7	Gaussian	615.1	5.2%	1.6	0.1
		265.0	47.5	Gaussian	5184.5			
		351.2	14.1	Gaussian	3257.8			
		237.7	47.7	Gaussian	2689.7			
	-875cm	283.0	57.1	Gaussian	5007.6			
		365.0	38.5	Gaussian	8414.4			

Table S5.6: Area integration data from PTD peak fitting analyses for ***SCB2*** *from Figure S5.2.* Hg^{2+} *peaks have a maximum peak temperature of >175 °C,* Hg^0 *peaks <175 °C (red highlight). Quantitative data for* Hg^0 *fractions and concentrations are also included and calculated from sum of the integrated areas of all peaks.*

Sample		Max peak temp. (°C)	Max peak height	Peak Type	Integrated area	Proportion Hg^0	THg conc. (mg kg^{-1})	Hg^0 conc. (mg kg^{-1})
SCB2	-160cm	255.5	62.1	Gaussian	1828.4			
		276.2	43.4	Gaussian	2862.5			
	-180cm	243.9	62.4	Gaussian	1897.9			
		263.8	45.0	Gaussian	2724.9			
	-200cm	251.0	69.1	Gaussian	2293.6			
		271.2	35.3	Gaussian	2278.9			
	-220cm	250.7	54.9	Gaussian	1702.7			
		267.3	40.8	Gaussian	2238.3			
		294.8	12.9	Gaussian	1619.6			
	-240cm	287.8	68.0	Gaussian	2196.0			
		306.6	33.5	Gaussian	2326.2			
	-260cm	286.1	62.9	Gaussian	2222.7			
		312.4	49.9	Gaussian	3519.2			
		358.6	4.5	Gaussian	490.5			
	-280cm	294.6	74.8	Gaussian	2710.9			
		319.5	24.2	Gaussian	1993.5			
	-300cm	282.8	52.9	Gaussian	1938.3			
		309.0	57.3	Gaussian	3740.5			
		342.5	14.3	Gaussian	1803.4			
	-320cm	295.0	69.1	Gaussian	2747.3			
		321.2	40.5	Gaussian	3031.6			
	-340cm	283.4	75.5	Gaussian	2678.0			
		310.1	26.6	Gaussian	2300.9			
	-375cm	277.4	80.9	Gaussian	3270.0			
		310.9	29.2	Gaussian	2487.8			
	-425cm	267.8	57.8	Gaussian	2230.9			
		298.7	57.1	Gaussian	4310.0			
		337.8	13.4	Gaussian	1542.6			
	-475cm	281.8	74.7	Gaussian	4333.9			
		318.1	28.8	Gaussian	3011.1			
	-525cm	263.5	68.0	Gaussian	2747.2			
		282.8	28.9	Gaussian	2581.2			
	-575cm	249.2	48.0	Gaussian	1812.9			
		272.8	53.7	Gaussian	4337.6			
		343.7	10.7	Gaussian	1787.6			
	-625cm	265.3	56.1	Gaussian	3227.8			
		247.1	44.3	Gaussian	1399.8			
		303.8	17.9	Gaussian	2293.2			
	-675cm	245.3	47.8	Gaussian	1636.3			
		265.1	56.1	Gaussian	3651.0			
		317.0	12.3	Gaussian	1451.9			
	-725cm	250.5	63.1	Gaussian	2249.7			
		271.5	41.0	Gaussian	3192.9			
	-775cm	248.3	40.5	Gaussian	1500.1			
		263.7	52.0	Gaussian	4394.8			
		335.3	15.4	Gaussian	1897.5			
		484.7	9.8	Gaussian	1632.0			
	-825cm	238.9	71.9	Gaussian	3011.1			
		270.3	25.9	Gaussian	3032.6			
		448.8	15.9	Gaussian	2711.1			
	-875cm	145.1	4.9	Gaussian	378.1	2.3%	5.4	0.1
		478.2	16.9	Gaussian	3231.3			
		253.0	94.7	Gaussian	9647.0			
		342.9	27.6	Gaussian	3274.1			
	-925cm	161.5	5.6	Gaussian	251.0	2.1%	12.1	0.2
		235.9	51.0	Gaussian	3652.7			
		263.4	49.2	Gaussian	6810.8			
		430.5	6.5	Gaussian	1441.3			

Continued on next page.

Table S5.6 continued.

Sample		Max peak temp. (°C)	Max peak height	Peak Type	Integrated area	Proportion Hg^0	THg conc. (mg kg^{-1})	Hg^0 conc. (mg kg^{-1})
SCB2	-975cm	248.2	7.5	Gaussian	316.6			
		499.7	21.6	Gaussian	3193.1			
		409.7	10.5	Gaussian	753.6			
		275.5	94.3	Gaussian	13269.3			
	-1015cm	264.8	52.8	Gaussian	7917.6			
		241.4	48.3	Gaussian	3191.9			
		433.4	10.2	Gaussian	2333.8			
	-1040cm	240.0	60.0	Gaussian	3956.0			
		284.4	43.7	Gaussian	5329.9			
		369.8	18.3	Gaussian	4551.8			
	-1060cm	235.8	52.6	Gaussian	2929.3			
		371.3	21.1	Gaussian	4682.9			
		273.0	51.4	Gaussian	6018.2			
	-1085cm	245.7	53.9	Gaussian	3353.6			
		438.0	16.8	Gaussian	3696.4			
		285.1	52.4	Gaussian	7438.0			
	-1115cm	245.5	61.3	Gaussian	4307.2			
		293.0	49.2	Gaussian	7504.7			
		443.1	23.6	Gaussian	4756.3			
	-1145cm	238.0	57.7	Gaussian	4818.8			
		280.9	48.4	Gaussian	7563.3			
		449.5	8.0	Gaussian	1610.0			
	-1175cm	253.9	50.5	Gaussian	2875.7			
		288.6	57.9	Gaussian	8306.5			
		468.6	11.3	Gaussian	2377.7			
	-1205cm	247.6	52.2	Gaussian	4666.4			
		297.2	58.9	Gaussian	10780.8			
		484.0	50.5	Gaussian	9140.2			
	-1235cm	241.9	51.9	Gaussian	3148.8			
		284.2	60.2	Gaussian	8188.5			
		426.3	21.6	Gaussian	5127.8			
	-1265cm	244.0	44.4	Gaussian	2669.5			
		282.6	54.0	Gaussian	6546.0			
		450.9	37.1	Gaussian	8581.6			
	-1295cm	254.0	47.8	Gaussian	2846.4			
		292.8	54.8	Gaussian	6382.4			
		391.0	22.7	Gaussian	5902.5			
	-1325cm	256.3	46.4	Gaussian	2790.4			
		388.6	17.5	Gaussian	4662.8			
		289.5	52.7	Gaussian	6204.3			
	-1355cm	241.2	60.1	Gaussian	4035.4			
		316.5	70.1	Gaussian	12144.6			
		488.0	28.2	Gaussian	4162.2			
	-1385cm	238.8	53.6	Gaussian	3131.2			
		276.9	48.1	Gaussian	5762.8			
		361.3	18.1	Gaussian	4622.8			

*Table S5.7: Area integration data from PTD peak fitting analyses for **SCB3** from Figure S5.2. Hg^{2+} peaks have a maximum peak temperature of >175 °C, Hg^0 peaks <175 °C (red highlight). Quantitative data for Hg^0 fractions and concentrations are also included and calculated from sum of the integrated areas of all peaks.*

Sample		Max peak temp. (°C)	Max peak height	Peak Type	Integrated area	Proportion Hg^0	THg conc. (mg kg^{-1})	Hg^0 conc. (mg kg^{-1})
SCB3	-535cm	142.2	3.7	Gaussian	120.7	0.7%	2.1	0.0
		232.5	51.7	Gaussian	2965.8			
		284.2	64.9	Gaussian	7698.0			
		403.9	35.9	Gaussian	7431.7			
	-630cm	112.2	3.3	Gaussian	62.4	0.3%	10.1	0.0
		225.4	42.7	Gaussian	2260.6			
		267.6	65.2	Gaussian	6318.9			
		404.1	49.0	Gaussian	11228.8			
	-705cm	124.4	10.7	Gaussian	531.7	2.2%	14.2	0.3
		210.9	38.5	Gaussian	2263.2			
		257.6	73.0	Gaussian	9516.7			
		421.3	48.6	Gaussian	11514.8			
	-775cm	130.9	12.7	Gaussian	395.2	2.0%	19.9	0.4
		214.3	50.0	Gaussian	2950.7			
		266.6	70.5	Gaussian	8731.3			
		426.4	36.6	Gaussian	7627.0			
	-825cm	136.8	16.2	Gaussian	523.0	2.8%	0.2	0.0
		216.5	53.6	Gaussian	3208.6			
		267.1	62.5	Gaussian	8006.2			
		412.3	29.9	Gaussian	7026.7			
	-875cm	152.0	7.6	Gaussian	232.0	1.2%	0.2	0.0
		223.5	36.4	Gaussian	2299.1			
		265.6	72.1	Gaussian	10504.8			
		443.3	32.5	Gaussian	6865.2			
	-950cm	237.7	40.6	Gaussian	2595.8			
		254.4	57.5	Gaussian	8236.5			
		430.7	7.3	Gaussian	1782.5			
	-1050cm	238.2	39.6	Gaussian	2329.4			
		287.7	85.9	Gaussian	8174.1			
		387.2	55.2	Gaussian	5290.0			
		478.2	61.6	Gaussian	9003.0			
	-1150cm	181.0	20.8	Gaussian	921.8			
		261.8	65.9	Gaussian	3905.9			
		304.4	45.9	Gaussian	6315.4			

S6. Solid-phase THg, pH, moisture content and Hg stable isotopes

Soil pH was measured with a calibrated glass pH electrode after 1 h equilibration of ≈5 – 10 g of wet soil mixed and shaken in 50 mL of 0.01 M $CaCl_2$ solution (Section S6).

Note: In all tables, letters next to sample names denote replicates.

Table S6.1: THg concentrations, pH, and Hg stable isotope (‰) data from ***SCA1****. Samples marked with '*' were pre-concentrated for isotope analysis. Standard deviation of triplicate digests is reported in brackets.*

Sample		Hg Conc. dw (mg kg^{-1})	pH	δ^{202}Hg Value	δ^{202}Hg 2SD	Δ^{199}Hg Value	Δ^{199}Hg 2SD	Δ^{200}Hg Value	Δ^{200}Hg 2SD	Δ^{201}Hg Value	Δ^{201}Hg 2SD	Δ^{204}Hg Value	Δ^{204}Hg 2SD
SCA1	-0.5m	18.34	9.89	-0.18	0.04	-0.04	0.04	0.09	0.13	-0.04	0.02	-0.07	0.06
	-1.5m	20.30	10.60	-0.23	0.04	-0.05	0.04	0.09	0.13	-0.06	0.02	-0.02	0.06
	-2.5m*	0.12	7.42	-0.81	0.11	-0.02	0.07	0.03	0.06	-0.06	0.16	-0.05	0.16
	-3.5m	1.91	6.63	-0.09	0.10	-0.03	0.05	-0.05	0.16	-0.06	0.04	0.02	0.05
	-4.5m	8.67	6.35	-0.50	0.10	-0.03	0.05	-0.17	0.16	-0.05	0.04	0.04	0.05
	-5.5m	10.75	6.31	-0.63	0.04	0.01	0.04	-0.07	0.13	-0.05	0.02	0.00	0.06
	-6.5m	10.69	6.60	-0.56	0.04	-0.01	0.04	0.09	0.13	0.00	0.02	-0.01	0.06
	-7.5m	12.65	5.82	-0.52	0.04	0.01	0.04	0.09	0.13	-0.02	0.02	0.00	0.06
	-8.5m	12.57	6.06	-0.39	0.04	-0.02	0.04	0.04	0.13	-0.03	0.02	0.04	0.06
	-9.5m*	1.65 (± 0.09)	6.01	-0.49	0.11	-0.07	0.07	-0.04	0.06	-0.09	0.16	-0.01	0.16
	-10.5m*	0.96	6.02	-0.46	0.11	-0.03	0.07	0.00	0.06	-0.07	0.16	-0.13	0.16
	-11.5m*	0.15	5.94	-0.89	0.11	0.04	0.07	0.01	0.06	-0.02	0.16	-0.14	0.16
	-12.5m*	0.10	5.76	-0.80	0.11	0.09	0.07	0.05	0.06	0.00	0.16	-0.23	0.16
	-13.5m*	0.22	6.08	-0.52	0.11	0.04	0.07	-0.01	0.06	0.03	0.16	-0.02	0.16

Table S6.2: THg concentrations, pH, and Hg stable isotope (‰) data from ***SCA2****. Samples marked with '*' were pre-concentrated for isotope analysis. Standard deviation of triplicate digests is reported in brackets.*

Sample		Hg Conc. dw (mg kg^{-1})	pH	δ^{202}Hg Value	δ^{202}Hg 2SD	Δ^{199}Hg Value	Δ^{199}Hg 2SD	Δ^{200}Hg Value	Δ^{200}Hg 2SD	Δ^{201}Hg Value	Δ^{201}Hg 2SD	Δ^{204}Hg Value	Δ^{204}Hg 2SD
SCA2	-0.5m	9.10	8.77	-0.33	0.10	-0.05	0.05	0.09	0.16	-0.02	0.04	0.01	0.05
	-1.5m	24.98	8.30	-0.35	0.10	0.00	0.05	-0.05	0.16	-0.04	0.04	0.01	0.05
	-2.5m	30.26	7.80	-0.06	0.04	-0.02	0.04	-0.03	0.13	0.00	0.02	-0.01	0.06
	-3.5m	27.78 (± 1.40)	7.78	-0.37	0.10	-0.07	0.05	0.03	0.16	-0.06	0.04	0.02	0.05
	-4.5m	24.24	7.74	-0.45	0.10	-0.04	0.05	-0.03	0.16	-0.04	0.04	-0.03	0.05
	-5.5m	31.94	7.39	-0.44	0.10	-0.05	0.05	0.03	0.16	-0.04	0.04	0.00	0.05
	-6.5m	50.10	7.42	-0.32	0.10	-0.04	0.05	-0.07	0.16	-0.05	0.04	-0.02	0.05
	-7.5m	23.31	7.40	0.04	0.10	-0.06	0.05	-0.03	0.16	-0.10	0.04	-0.02	0.05
	-8.5m*	0.52	5.93	-2.03	0.13	0.08	0.07	0.02	0.04	-0.06	0.04	-0.04	0.13
	-9.5m*	1.37	5.95	-0.70	0.13	-0.01	0.07	0.03	0.04	0.00	0.04	-0.01	0.13
	-10.5m*	0.45	5.77	-0.83	0.12	0.02	0.07	-0.01	0.05	-0.10	0.05	0.03	0.10
	-11.5m	2.53	5.80	0.03	0.10	-0.11	0.05	0.10	0.16	-0.06	0.04	-0.04	0.05
	-12.5m	2.14	5.93	0.10	0.10	-0.07	0.05	0.05	0.16	-0.05	0.04	0.04	0.05
	-13.5m	2.92	6.02	-0.10	0.10	0.00	0.05	-0.03	0.16	-0.01	0.04	-0.06	0.05
	-14.5m*	0.74	5.95	0.45	0.11	-0.09	0.07	-0.01	0.06	-0.11	0.16	-0.01	0.16
	-15.5m*	0.04	5.99	-1.35	0.11	-0.02	0.07	0.00	0.06	-0.02	0.16	0.02	0.16
	-16.5m*	0.10	5.97	-0.92	0.11	0.04	0.07	0.00	0.06	0.00	0.16	0.07	0.16
	-17.5m*	0.07	6.14	-1.19	0.11	0.00	0.07	0.03	0.06	-0.01	0.16	0.09	0.16
	-18.5m*	0.09 (± 0.01)	6.78	-1.01	0.11	0.00	0.07	0.05	0.06	-0.05	0.16	-0.03	0.16
	-19.5m*	0.12	6.81	-0.81	0.11	0.05	0.07	0.02	0.06	0.00	0.16	-0.01	0.16

*Table S6.3: THg concentrations, pH, and Hg stable isotope (‰) data from **SCA3**. Samples marked with '*' were pre-concentrated for isotope analysis. Standard deviation of triplicate digests is reported in brackets.*

Sample		Hg Conc. dw (mg kg^{-1})	pH	δ^{202}Hg Value	2SD	Δ^{199}Hg Value	2SD	Δ^{200}Hg Value	2SD	Δ^{201}Hg Value	2SD	Δ^{204}Hg Value	2SD
SCA3	-0.1m	23.35	7.71	-0.19	0.06	-0.03	0.03	-0.02	0.04	-0.05	0.02	-0.05	0.07
	-0.3m	56.93 (± 1.27)	7.43	0.12	0.06	-0.03	0.03	-0.02	0.04	-0.02	0.02	0.05	0.07
	-0.5m	73.80	6.96	0.41	0.06	-0.04	0.03	0.00	0.04	0.01	0.02	0.01	0.07
	-0.7m	76.30	6.76	0.20	0.06	-0.06	0.03	-0.02	0.04	-0.03	0.02	0.05	0.07
	-0.9m	78.24	6.58	0.30	0.06	-0.06	0.03	0.03	0.04	-0.02	0.02	-0.04	0.07
	-1.1m	40.46 (± 2.84)	7.54	0.32	0.06	-0.05	0.03	-0.01	0.04	-0.02	0.02	0.00	0.07
	-1.3m	103.90	7.25	0.16	0.06	-0.04	0.03	0.06	0.04	-0.05	0.02	0.04	0.07
	-1.5m	133.11	7.51	-0.03	0.06	-0.04	0.03	0.05	0.04	-0.06	0.02	-0.05	0.07

*Table S6.4: THg concentrations, pH, and Hg stable isotope (‰) data from site A topsoil samples (**TSA**). Samples marked with '*' were pre-concentrated for isotope analysis. Standard deviation of triplicate digests is reported in brackets.*

Sample		Hg Conc. dw (mg kg^{-1})	pH	δ^{202}Hg Value	2SD	Δ^{199}Hg Value	2SD	Δ^{200}Hg Value	2SD	Δ^{201}Hg Value	2SD	Δ^{204}Hg Value	2SD
TSA	TSA1	17.41	7.91	-0.81	0.04	0.01	0.04	0.06	0.13	-0.01	0.02	0.03	0.06
	TSA2	2.28	7.56	-0.80	0.04	-0.01	0.04	0.20	0.13	-0.04	0.02	0.05	0.06
	TSA3	318.65	8.12	-0.13	0.10	-0.03	0.05	-0.17	0.16	-0.05	0.04	0.04	0.05
	TSA4	142.56	8.07	-0.30	0.10	-0.05	0.05	-0.05	0.16	-0.04	0.04	0.03	0.05
	TSA5	98.42 (± 2.59)	8.09	0.06	0.04	-0.07	0.04	0.01	0.13	-0.03	0.02	0.00	0.06
	TSA6	10.61	8.19	-2.93	0.04	0.11	0.04	0.04	0.13	0.05	0.02	0.02	0.06
	TSA7	7.54	8.08	-1.84	0.04	0.10	0.04	0.08	0.13	0.04	0.02	0.03	0.06
	TSA8*	0.45	8.15	-1.46	0.06	-0.03	0.02	-0.22	0.13	-0.02	0.04	-0.07	0.08
	TSA9	1.61	8.13	-1.97	0.04	0.13	0.04	0.01	0.13	0.10	0.02	0.04	0.06
	TSA10	83.78	7.76	-0.23	0.04	-0.07	0.04	-0.03	0.13	-0.07	0.02	0.01	0.06
	TSA11	2.81	7.33	-0.23	0.04	-0.06	0.04	-0.03	0.13	-0.05	0.02	-0.02	0.06
	SSA11	41.50	6.98	-0.29	0.04	-0.05	0.04	0.11	0.13	-0.01	0.02	0.02	0.06

*Table S6.5: THg concentrations, pH, and Hg stable isotope (‰) data from **SCB1**. Samples marked with '*' were pre-concentrated for isotope analysis. Standard deviation of triplicate digests is reported in brackets.*

Sample		Hg Conc. dw (mg kg^{-1})	pH	δ^{202}Hg Value	2SD	Δ^{199}Hg Value	2SD	Δ^{200}Hg Value	2SD	Δ^{201}Hg Value	2SD	Δ^{204}Hg Value	2SD
SCB1	-0.25m	66.29	7.40	0.49	0.05	-0.09	0.04	0.00	0.03	-0.05	0.05	0.02	0.05
	-0.75m	18.45	7.47	-0.23	0.03	0.00	0.03	-0.01	0.03	-0.03	0.03	0.03	0.02
	-1.25m	515.67	8.67	-0.32	0.05	-0.05	0.04	-0.02	0.03	-0.06	0.05	0.01	0.05
	-1.75m	251.83	11.37	-0.08	0.05	-0.06	0.04	0.01	0.03	-0.03	0.05	0.01	0.05
	-2.25m	266.99 (± 42.84)	11.34	-0.10	0.04	-0.08	0.02	-0.01	0.01	-0.10	0.03	0.02	0.08
	-2.75m	562.33	11.14 (± 0.04)	-0.16	0.05	-0.02	0.04	0.01	0.03	-0.05	0.05	0.00	0.05
	-3.25m	415.54	11.24	-0.17	0.05	-0.07	0.04	-0.01	0.03	-0.06	0.05	0.01	0.05
	-3.75m	26.45	8.24 (± 0.11)	0.25	0.06	-0.10	0.03	0.02	0.02	-0.09	0.03	0.01	0.04
	-4.25m	2.06	7.60	0.44	0.07	-0.04	0.05	-0.01	0.05	-0.03	0.08	-0.01	0.08
	-4.75m*	0.37	7.04	-1.01	0.07	-0.02	0.06	-0.04	0.08	0.01	0.13	0.02	0.14
	-5.25m	3.61	6.99	0.09	0.06	-0.11	0.03	-0.02	0.02	-0.09	0.03	0.00	0.04
	-5.75m	6.31	7.06	0.28	0.06	-0.13	0.03	-0.04	0.02	-0.15	0.03	-0.02	0.04
	-6.25m	10.09	6.75	0.09	0.06	-0.10	0.03	0.01	0.02	-0.09	0.03	0.01	0.04
	-6.75m	12.51	6.80	0.35	0.06	-0.11	0.03	0.00	0.02	-0.11	0.03	-0.05	0.04
	-7.25m	5.63	6.79	0.29	0.06	-0.13	0.03	-0.01	0.02	-0.10	0.03	0.02	0.04
	-7.75m	2.81	6.79	0.27	0.07	-0.12	0.05	0.00	0.05	-0.15	0.08	-0.01	0.08
	-8.25m	1.59	6.72	0.25	0.07	-0.09	0.05	0.00	0.05	-0.09	0.08	-0.03	0.08
	-8.75m*	0.16	6.39	-1.17	0.07	-0.07	0.06	0.00	0.08	-0.05	0.13	-0.04	0.14
	-9.25m*	0.05	6.36	-1.09	0.07	-0.05	0.06	0.02	0.08	-0.06	0.13	-0.01	0.14
	-9.75m*	0.04	6.37	-2.48	0.13	-0.05	0.07	-0.03	0.04	-0.05	0.04	0.03	0.13
	-10.25m*	0.04	6.33	-2.70	0.13	-0.04	0.07	0.01	0.04	-0.01	0.04	0.11	0.13
	-10.75m*	0.04	6.35	-1.93	0.13	-0.06	0.07	-0.03	0.04	-0.05	0.04	0.22	0.13
	-11.25m*	0.04 (± 0.01)	6.32	-2.70	0.13	-0.07	0.07	-0.08	0.04	-0.12	0.04	0.01	0.13
	-11.75m*	0.03	6.27	-2.46	0.13	-0.03	0.07	0.05	0.04	0.02	0.04	0.01	0.13
	-12.25m*	0.04	6.25	-2.62	0.13	-0.03	0.07	0.01	0.04	-0.02	0.04	0.16	0.13
	-12.75m*	0.04	6.27	-2.37	0.13	0.00	0.07	0.02	0.04	-0.13	0.04	-0.04	0.13
	-13.25m*	0.06	6.23	-2.48	0.13	0.01	0.07	0.00	0.04	-0.07	0.04	-0.17	0.13
	-13.75m*	0.04	6.24	-2.25	0.13	0.02	0.07	-0.01	0.04	-0.05	0.04	-0.14	0.13
	-14.25m*	0.03	6.22	-2.22	0.13	0.03	0.07	0.02	0.04	-0.03	0.04	-0.05	0.13
	-14.75m*	0.03	6.21	-2.23	0.13	0.00	0.07	0.03	0.04	-0.03	0.04	0.09	0.13
	-15.25m*	0.03	6.21 (± 0.03)	-2.48	0.13	-0.03	0.07	0.01	0.04	-0.06	0.04	-0.04	0.13
	-15.75m*	0.04	6.13	-2.63	0.13	0.02	0.07	0.04	0.04	-0.05	0.04	-0.05	0.13
	-16.25m*	0.17	6.08	-1.43	0.13	-0.06	0.07	0.00	0.04	-0.04	0.04	0.01	0.13
	-16.75m*	0.05	6.09	-2.52	0.13	0.02	0.07	0.03	0.04	0.02	0.04	0.05	0.13
	-17.25m*	0.04	6.05	-2.45	0.13	0.01	0.07	-0.03	0.04	-0.05	0.04	-0.06	0.13

*Table S6.6: THg concentrations, pH, and Hg stable isotope (‰) data from **SCB2**. Samples marked with '*' were pre-concentrated for isotope analysis. Standard deviation of triplicate digests is reported in brackets.*

Sample		Hg Conc. dw (mg kg^{-1})	pH	δ^{202}Hg		Δ^{199}Hg		Δ^{200}Hg		Δ^{201}Hg		Δ^{204}Hg	
				Value	2SD	Value	2SD	Value	2SD	Value	2SD	Value	2SD
SCB2	-1.6m*	1.75	7.31 (± 0.01)	-0.39	0.11	-0.02	0.07	0.01	0.06	-0.01	0.16	-0.01	0.16
	-1.8m*	2.16	7.28	-0.36	0.11	-0.01	0.07	0.03	0.06	0.04	0.16	-0.01	0.16
	-2m*	0.79	7.47	-0.58	0.11	0.06	0.07	0.01	0.06	0.00	0.16	0.00	0.16
	-2.2m*	0.46	7.56	-0.42	0.11	0.06	0.07	-0.02	0.06	-0.03	0.16	0.02	0.16
	-2.4m*	0.12	7.46	-0.74	0.11	-0.02	0.08	-0.01	0.06	0.02	0.09	0.00	0.17
	-2.6m*	2.01	7.27	-0.10	0.12	0.01	0.07	0.02	0.08	0.02	0.03	-0.01	0.05
	-2.8m*	0.31	7.25	-0.36	0.11	-0.05	0.08	0.01	0.06	-0.08	0.09	-0.05	0.17
	-3m*	2.35	7.27	-0.43	0.11	0.02	0.07	-0.01	0.06	0.02	0.16	-0.02	0.16
	-3.2m*	1.62	7.28	0.12	0.11	0.04	0.07	0.01	0.06	-0.02	0.16	-0.13	0.16
	-3.4m*	0.18	7.59	-0.98	0.11	-0.06	0.08	-0.01	0.06	-0.07	0.09	-0.01	0.17
	-3.75m*	0.25	7.64	-0.25	0.11	-0.03	0.08	-0.01	0.06	-0.06	0.09	0.08	0.17
	-4.25m	9.25 (± 1.18)	7.25	0.17	0.11	-0.03	0.08	0.03	0.06	-0.02	0.09	-0.04	0.17
	-4.75m*	0.75	7.59	-0.20	0.11	0.01	0.07	0.04	0.06	0.01	0.16	0.01	0.16
	-5.25m*	0.07	7.78	-0.11	0.11	-0.02	0.08	-0.01	0.06	-0.05	0.09	-0.75	0.17
	-5.75m*	0.35	7.79	-1.05	0.11	0.00	0.08	-0.03	0.06	0.01	0.09	0.06	0.17
	-6.25m*	0.23	7.78	-0.56	0.11	-0.02	0.08	0.02	0.06	0.00	0.09	0.11	0.17
	-6.75m*	0.86	7.69	-0.14	0.07	-0.06	0.06	0.01	0.08	-0.04	0.13	0.00	0.14
	-7.25m*	0.16	7.59	-0.34	0.07	-0.05	0.06	-0.03	0.08	-0.05	0.13	0.05	0.14
	-7.75m*	0.88	7.72	-0.52	0.12	0.01	0.07	0.03	0.08	-0.05	0.03	0.01	0.05
	-8.25m*	0.14	7.74	-1.17	0.07	0.03	0.06	0.00	0.08	0.03	0.13	0.03	0.14
	-8.75m	5.45	7.65	0.13	0.07	-0.03	0.02	0.03	0.06	-0.06	0.05	0.04	0.04
	-9.25m	12.11	7.68	0.13	0.05	-0.06	0.01	0.11	0.09	-0.04	0.03	-0.01	0.01
	-9.75m	20.68	7.65	-0.06	0.07	-0.05	0.02	0.05	0.06	-0.07	0.05	0.03	0.04
	-10.15m	17.36	7.69	0.00	0.05	-0.04	0.03	0.02	0.18	-0.05	0.03	0.01	0.04
	-10.4m	19.23	7.63	-0.14	0.07	-0.05	0.02	-0.01	0.06	-0.05	0.05	-0.01	0.04
	-10.6m	19.53	7.68	-0.08	0.05	-0.04	0.03	0.02	0.18	-0.06	0.03	-0.02	0.04
	-10.85m	20.51	7.75	-0.12	0.07	-0.06	0.02	-0.02	0.06	-0.05	0.05	-0.01	0.04
	-11.15m	16.47	7.49	-0.19	0.05	-0.05	0.03	0.16	0.18	-0.06	0.03	-0.04	0.04
	-11.45m	20.32	7.30	-0.12	0.08	0.01	0.03	0.06	0.08	-0.06	0.07	-0.01	0.04
	-11.75m	34.08	6.59	-0.19	0.05	-0.02	0.03	0.04	0.18	-0.01	0.03	-0.05	0.04
	-12.05m	30.79	6.68	-0.16	0.08	-0.03	0.03	0.03	0.08	-0.01	0.07	-0.02	0.04
	-12.35m	26.42 (± 5.13)	6.89	-0.28	0.05	-0.01	0.03	0.15	0.18	-0.03	0.03	-0.02	0.04
	-12.65m	39.59	7.16	-0.18	0.08	-0.03	0.03	0.07	0.08	-0.08	0.07	0.04	0.04
	-12.95m	14.77	6.86	-0.17	0.05	-0.08	0.03	-0.04	0.18	-0.06	0.03	0.01	0.04
	-13.25m	26.67 (± 7.62)	6.69	-0.17	0.08	-0.02	0.03	0.08	0.08	-0.07	0.07	0.00	0.04
	-13.55m	22.40	6.74	-0.12	0.05	-0.02	0.03	0.13	0.18	-0.06	0.03	0.01	0.04
	-13.85m	19.03	7.02	-0.07	0.08	-0.01	0.03	0.01	0.08	-0.03	0.07	-0.07	0.15
	-14.15m*	1.12	6.52	-0.01	0.11	0.01	0.07	0.03	0.06	-0.06	0.16	-0.09	0.16
	-14.45m*	0.88	6.31	-0.18	0.11	-0.03	0.07	0.02	0.06	-0.05	0.16	0.04	0.16
	-14.45m*	0.88	6.31	-0.19	0.12	-0.07	0.07	-0.01	0.05	-0.09	0.05	-0.04	0.10
	-14.75m*	0.70	6.42	-0.30	0.11	-0.02	0.07	0.01	0.06	-0.06	0.16	0.08	0.16
	-15.05m*	0.10	6.54	-0.45	0.07	-0.04	0.06	0.06	0.08	-0.06	0.13	0.03	0.14

*Table S6.7: THg concentrations, pH, and Hg stable isotope (‰) data from **SCB3**. Samples marked with '*' were pre-concentrated for isotope analysis. Standard deviation of triplicate digests is reported in brackets.*

Sample		Hg Conc. dw (mg kg^{-1})	pH	δ^{202}Hg Value	δ^{202}Hg 2SD	Δ^{199}Hg Value	Δ^{199}Hg 2SD	Δ^{200}Hg Value	Δ^{200}Hg 2SD	Δ^{201}Hg Value	Δ^{201}Hg 2SD	Δ^{204}Hg Value	Δ^{204}Hg 2SD
SCB3	-0.5m*	0.17	7.68	-1.93	0.07	0.00	0.06	0.04	0.08	-0.05	0.13	0.05	0.14
SCB3	-1.5m*	0.06	7.72	-3.09	0.07	0.09	0.06	0.03	0.08	0.06	0.13	-0.07	0.14
SCB3	-2.4m*	0.02	7.84	-3.68	0.07	0.11	0.06	-0.02	0.08	0.04	0.13	-0.04	0.14
SCB3	-3.05m*	0.02	7.39	-3.05	0.07	0.03	0.06	0.05	0.08	0.02	0.13	-0.02	0.14
SCB3	-3.65m*	0.02	7.77	-3.31	0.07	0.07	0.06	0.00	0.08	-0.03	0.13	-0.01	0.14
SCB3	-4.35m*	0.23	7.61	-1.55	0.12	-0.03	0.07	-0.04	0.05	0.01	0.05	0.05	0.10
SCB3	-5.35m	2.13	7.18	0.42	0.07	-0.05	0.05	0.02	0.05	-0.06	0.08	-0.07	0.08
SCB3	-6.3m	10.13	7.02	0.34	0.06	-0.08	0.03	0.02	0.02	-0.05	0.03	0.09	0.04
SCB3	-7.05m	14.21	7.03	0.06	0.06	-0.06	0.03	0.00	0.02	-0.08	0.03	0.03	0.04
SCB3	-7.75m	19.93	6.76	0.00	0.06	-0.05	0.03	0.02	0.02	-0.05	0.03	0.03	0.04
SCB3	-8.25m	10.95	6.63	0.16	0.06	-0.05	0.03	0.01	0.02	-0.06	0.03	0.00	0.04
SCB3	-8.75m	9.85	6.59	0.23	0.06	-0.10	0.03	-0.01	0.02	-0.05	0.03	-0.01	0.04
SCB3	-9.5m	10.14	6.49	0.09	0.06	-0.06	0.03	-0.01	0.02	-0.06	0.03	0.03	0.04
SCB3	-10.5m	12.04	6.48	0.15	0.06	-0.10	0.03	-0.02	0.02	-0.08	0.03	0.00	0.04
SCB3	-11.5m	2.99 (± 0.11)	6.45	0.29	0.03	-0.11	0.01	-0.03	0.01	-0.09	0.03	0.03	0.01
SCB3	-12.5m*	0.05	6.31 (± 0.01)	-1.32	0.07	-0.07	0.06	0.00	0.08	-0.05	0.13	0.01	0.14
SCB3	-13.5m*	0.05	6.31	-0.68	0.07	0.00	0.06	0.03	0.08	0.04	0.13	0.00	0.14
SCB3	-14.5m*	0.02	6.28	-1.19	0.07	-0.02	0.06	0.01	0.08	-0.07	0.13	-0.07	0.14

Table S6.8: Mean absolute difference (‰) for repeated Hg stable isotope analyses of

A) Same digests analysed twice

	δ^{202}Hg	Δ^{199}Hg	Δ^{200}Hg	Δ^{201}Hg	Δ^{204}Hg
Mean	0.03	0.03	0.04	0.05	0.04
1SD	0.02	0.02	0.05	0.02	0.02
n	5	5	5	5	5

B) Same pre-concentrated sample analysed twice

	δ^{202}Hg	Δ^{199}Hg	Δ^{200}Hg	Δ^{201}Hg	Δ^{204}Hg
Mean	0.03	0.03	0.02	0.04	0.04
1SD	0.02	0.02	0.01	0.01	0.03
n	3	3	3	3	3

C) Same sample analysed as digest and pre-concentrated

	δ^{202}Hg	Δ^{199}Hg	Δ^{200}Hg	Δ^{201}Hg	Δ^{204}Hg
Mean	0.05	0.04	0.06	0.04	0.09
1SD	0.01	0.03	0.04	0.02	0.06
n	4	4	4	4	4

Table S6.9: Averages of samples with Hg stable isotopes analysis (‰) of replicate digests (n=3)

Sample		δ^{202}Hg	Δ^{199}Hg	Δ^{200}Hg	Δ^{201}Hg	Δ^{204}Hg
SCB1 -2.25m	Mean	0.10	-0.08	-0.01	-0.10	0.02
SCB1 -2.25m	1SD	0.04	0.02	0.01	0.03	0.03
SCB3 -11.5m	Mean	0.28	-0.11	-0.03	-0.09	0.03
SCB3 -11.5m	1SD	0.03	0.01	0.01	0.03	0.01

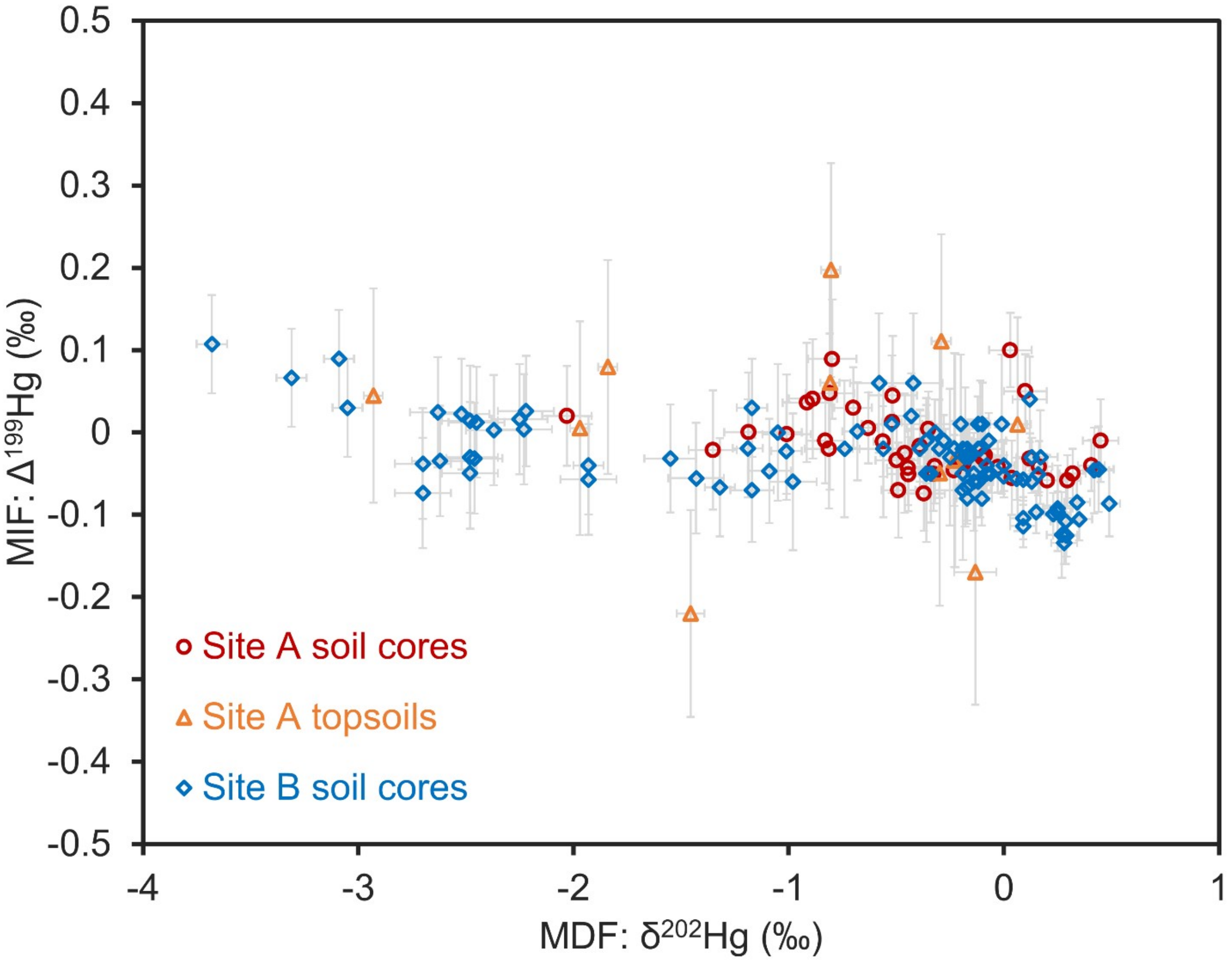

Figure 6.1: Plot of Δ^{199}Hg versus δ^{202}Hg for all solid phase materials analysed across both site A and site B. While the variability in the measured δ^{202}Hg values is large, the range of Δ^{199}Hg values is relatively small and the uncertainty (based on 2SD precision data) is large relative to the range of measured values.

S7. Sequential extraction procedure (SEP) data & SEP isotope data

Tables S7.1 – S7.5 show wet weight (ww) Hg concentrations, fractions (of sum of extract Hg concentrations), and δ^{202}Hg isotope signatures of individual SEP extracts. The ± values of the δ^{202}Hg values are the 2 SD values from the MC-ICP-MS analyses. When discussing the recoveries on THg concentration of ΣSEP extracts against measured THg concentration of bulk sample we must consider these are different samples and there is considerable heterogeneity of Hg within the contaminated solid phase material (Schroeder and Munthe, 1998; Miller et al., 2013). Thus, we cannot assume differences in these THg concentrations are attributable solely to SEP artefacts. The δ^{202}Hg value from the combined SEP extracts (δ^{202}Hg ΣSEP extracts) was calculated from equation S7.1 below:

$$\delta^{202}Hg\ \Sigma SEP = \left(\delta^{202}Hg_{F1} * [Hg]_{F1} + \delta^{202}Hg_{F2} * [Hg]_{F2} + \delta^{202}Hg_{F3} * [Hg]_{F3} + \delta^{202}Hg_{F4} * [Hg]_{F4}\right) \Big/ \Sigma[Hg]_{F1-F4}$$

Where *$[Hg]_{Fi}$* is the Hg concentration of fraction *i*. The uncertainty term of δ^{202}Hg ΣSEP extracts is fully propagated to include the 2 SD of the analyses, and the mean replicated variability (1 SD) from table S6.8. The uncertainty term for the "δ^{202}Hg difference: ΣSEP extracts to measured bulk" is propagated to include the uncertainty of the δ^{202}Hg ΣSEP extracts and the 2 SD uncertainty of the bulk measured δ^{202}Hg values.

Table S7.1: SEP Hg concentration, fraction, and stable isotope data from ***SCA1****.*

	Sample	F1 extract Hg conc. (mg kg^{-1})	F1 Fraction (%)	F1 δ^{202}Hg (‰)	F2 extract Hg conc. (mg kg^{-1})	F2 Fraction (%)	F2 δ^{202}Hg (‰)	F3 extract Hg conc. (mg kg^{-1})	F3 Fraction (%)	F3 δ^{202}Hg (‰)	F4 extract Hg conc. (mg kg^{-1})	F4 Fraction (%)	F4 δ^{202}Hg (‰)	ΣSEP Extract THg conc. (mg kg^{-1})	Measured bulk THg Conc. ww (mg kg^{-1})	Recovery ΣSEP THg to ww THg (%)	δ^{202}Hg ΣSEP extracts (‰)	δ^{202}Hg difference: ΣSEP extracts to measured bulk (‰)
**SCA1**	-0.5m	0.094	0.5%		0.046	0.2%		4.278	22.2%		14.84	77.1%		19.26	18.39	105%		
	-1.5m	0.017	0.1%		0.286	1.7%		3.386	20.4%		12.91	77.8%		16.60	16.83	99%		
	-2.5m	0.000	0.0%		0.000	0.1%		0.050	41.3%		0.07	58.6%		0.12	0.11	108%		
	-3.5m	0.010	0.6%		0.010	0.6%		0.410	24.1%		1.27	74.8%		1.70	1.73	99%		
	-4.5m	0.164	1.6%		3.285	32.4%		0.764	7.5%		5.92	58.4%		10.13	7.90	128%		
	-5.5m	0.034	0.4%		0.679	7.3%		1.796	19.4%		6.75	72.9%		9.25	9.31	99%		
	-6.5m	0.368	2.9%		5.843	45.5%		1.544	12.0%		5.08	39.6%		12.84	8.80	146%		
	-7.5m	0.109	1.0%		5.070	48.6%		2.201	21.1%		3.05	29.2%		10.43	10.84	96%		
	-8.5m	0.307	5.2%		0.000	0.0%		2.427	40.8%		3.22	54.1%		5.95	10.54	57%		
	-9.5m	0.010	0.9%		0.095	8.3%		0.488	42.7%		0.55	48.1%		1.14	1.38	83%		
	-10.5m	0.007	0.7%		0.017	1.8%		0.390	41.5%		0.53	55.9%		0.94	1.02	92%		
	-11.5m	0.002	1.2%		0.001	0.5%		0.085	48.0%		0.09	50.4%		0.18	0.14	125%		
	-12.5m	0.000	0.3%		0.000	0.6%		0.060	71.8%		0.02	27.3%		0.08	0.09	96%		
	-13.5m	0.001	0.2%		0.001	0.6%		0.130	56.3%		0.10	42.8%		0.23	0.19	118%		

Table S7.2: SEP Hg concentration, fraction, and stable isotope data from ***<u>SCA2</u>****.*

Sample		F1 extract Hg conc. (mg kg^{-1})	F1 extract Fraction (%)	F1 extract δ^{202}Hg (‰)	F2 extract Hg conc. (mg kg^{-1})	F2 extract Fraction (%)	F2 extract δ^{202}Hg (‰)	F3 extract Hg conc. (mg kg^{-1})	F3 extract Fraction (%)	F3 extract δ^{202}Hg (‰)	F4 extract Hg conc. (mg kg^{-1})	F4 extract Fraction (%)	F4 extract δ^{202}Hg (‰)	ΣSEP Extract THg conc. (mg kg^{-1})	Measured bulk THg Conc. ww (mg kg^{-1})	Recovery ΣSEP THg to ww THg (%)	δ^{202}Hg ΣSEP extracts (‰)	δ^{202}Hg difference: ΣSEP extracts to measured bulk (‰)
**SCA2**	-0.5m	0.011	0.1%		0.012	0.1%		5.385	47.8%		5.86	52.0%		11.27	8.55	132%		
	-1.5m	0.022	0.1%		0.070	0.4%		6.757	35.7%		12.10	63.8%		18.94	23.79	80%		
	-2.5m	0.085	0.4%	0.21±0.06	0.112	0.5%	0.10±0.06	8.325	37.1%	0.10±0.07	13.90	62.0%	-0.21±0.07	22.42	28.46	79%	-0.09±0.16	+0.04±0.18
	-3.5m	0.100	0.5%	-0.18±0.06	0.208	0.9%	-0.14±0.09	7.810	35.3%	-0.16±0.07	13.99	63.3%	-0.46±0.07	22.11	24.65	90%	-0.35±0.17	+0.02±0.20
	-4.5m	0.041	0.3%		0.113	0.7%	-0.31±0.06	5.712	37.9%	-0.27±0.07	9.21	61.1%	-0.47±0.09	15.08	21.99	69%	-0.40±0.16	+0.05±0.19
	-5.5m	0.055	0.2%		0.293	1.2%	-0.23±0.09	5.048	20.1%	-0.15±0.07	19.74	78.5%	-0.42±0.09	25.14	28.98	87%	-0.37±0.16	+0.08±0.19
	-6.5m	0.785	2.3%	0.09±0.07	5.845	17.2%	0.46±0.07	9.464	27.8%	0.24±0.07	17.90	52.7%	-0.05±0.07	33.99	46.22	74%	0.12±0.16	+0.44±0.19
	-7.5m	0.240	2.4%	3.08±0.09	0.455	4.6%	2.72±0.07	4.553	45.8%	2.18±0.07	4.68	47.2%	0.67±0.07	9.93	21.73	46%	1.51±0.18	+1.48±0.20
	-8.5m	0.002	0.4%		0.001	0.2%		0.461	82.2%		0.10	17.2%		0.56	0.46	122%		
	-9.5m	0.025	2.0%		0.002	0.2%		0.822	66.9%		0.38	30.9%		1.23	1.24	99%		
	-10.5m	0.006	1.9%		0.001	0.3%		0.240	75.8%		0.07	22.0%		0.32	0.41	77%		
	-11.5m	0.069	4.2%		0.042	2.5%		1.213	72.8%		0.34	20.6%		1.67	2.22	75%		
	-12.5m	0.020	1.4%		0.057	3.9%		0.979	67.4%		0.40	27.3%		1.45	1.95	75%		
	-13.5m	0.051	2.3%		0.198	9.1%		1.207	55.4%		0.72	33.2%		2.18	2.66	82%		
	-14.5m	0.008	1.5%		0.011	2.3%		0.362	73.3%		0.11	22.9%		0.49	0.66	74%		
	-16.5m	0.000	0.1%		0.000	0.0%		0.094	81.2%		0.02	18.7%		0.12	0.09	131%		
	-18.5m	0.000	0.0%		0.000	0.3%		0.052	85.6%		0.01	14.1%		0.06	0.07	83%		

*Table S7.3: SEP Hg concentration, fraction, and stable isotope data from **SCB1**. Values A, B, C are replicate extractions. THg of low concentration samples marked with '*' were analysed with a DMA-80 (Milestone) Hg analyser in triplicates (SD reported in brackets).*

Core	Sample	F1 extract Hg conc. (mg kg^{-1})	F1 Fraction (%)	F1 δ^{202}Hg (‰)	F2 extract Hg conc. (mg kg^{-1})	F2 Fraction (%)	F2 δ^{202}Hg (‰)	F3 extract Hg conc. (mg kg^{-1})	F3 Fraction (%)	F3 δ^{202}Hg (‰)	F4 extract Hg conc. (mg kg^{-1})	F4 Fraction (%)	F4 δ^{202}Hg (‰)	ΣSEP Extract THg conc. (mg kg^{-1})	Measured bulk THg Conc. ww (mg kg^{-1})	Recovery ΣSEP THg to ww THg (%)	δ^{202}Hg ΣSEP extracts (‰)	δ^{202}Hg difference: ΣSEP extracts to measured bulk (‰)
SCB1	-0.25m	0.667	1.2%	0.46±0.07	0.766	1.4%	0.39±0.07	6.69	11.9%	0.66±0.03	48.25	85.6%	0.50±0.06	56.37	58.98	96%	0.52±0.15	+0.03±0.16
	-0.75m	0.123	0.8%	1.29±0.06	0.130	0.8%	1.87±0.15	2.21	14.4%	0.05±0.05	12.90	84.0%	-0.31±0.06	15.36	16.23	95%	-0.22±0.2	+0.07±0.20
	-1.25m A	6.986	1.7%	-0.20±0.07	78.66	19.1%	-0.14±0.07	154.6	37.5%	-0.23±0.03	172.5	41.8%	-0.42±0.06	412.7	438.2	94%	-0.29±0.15	+0.03±0.16
	-1.25m B	7.835	1.6%		81.88	17.0%		192.4	40.0%		198.6	41.3%		480.7	438.2	110%		
	-1.75m	2.738	1.3%	0.15±0.07	49.22	23.1%	0.08±0.07	35.73	16.7%	0.05±0.06	125.7	58.9%	-0.14±0.06	213.4	228.2	93%	-0.06±0.16	+0.03±0.17
	-2.25m	3.109	1.4%	0.23±0.07	37.79	17.4%	0.25±0.07	31.60	14.6%	0.14±0.06	144.2	66.5%	-0.07±0.06	216.7	230.7	94%	0.02±0.16	+0.12±0.17
	-2.75m	11.98	2.7%	0.53±0.07	42.72	9.6%	0.10±0.07	109.3	24.4%	-0.02±0.06	283.1	63.3%	-0.21±0.06	447.1	478.8	93%	-0.11±0.16	+0.05±0.17
	-2.75m	13.59	1.9%		103.0	14.3%		208.5	29.0%		393.0	54.7%		718.1	478.8	150%		
	-3.25m	21.23	6.0%	0.33±0.06	19.81	5.6%	0.16±0.06	56.70	16.1%	0.00±0.06	254.0	72.2%	-0.14±0.06	351.7	369.5	95%	-0.07±0.15	+0.10±0.16
	-3.75m A	0.756	3.2%	0.48±0.07	0.202	0.9%	0.34±0.05	2.39	10.2%	0.48±0.06	20.14	85.7%	0.25±0.06	23.48	23.06	102%	0.28±0.15	+0.03±0.16
	-3.75m B	0.629	3.3%		0.172	0.9%		1.87	9.9%		16.22	85.8%		18.89	23.06	82%		
	-4.25m	0.133	7.9%	1.27±0.06	0.040	2.4%		0.39	23.4%	0.51±0.06	1.12	66.4%	0.20±0.09	1.69	1.71	99%	0.35±0.15	-0.09±0.17
	-4.75m	0.045	10.4%		0.004	1.0%		0.14	32.3%		0.24	56.3%		0.43	0.37* (± 0.07)	117%		
	-5.25m	0.135	5.1%		0.760	29.0%		0.45	17.1%		1.28	48.8%		2.62	3.51	75%		
	-5.75m	0.231	4.4%	0.49±0.05	2.499	47.4%	0.37±0.07	0.91	17.3%	0.18±0.05	1.63	31.0%	0.06±0.05	5.28	6.17	85%	0.25±0.14	-0.04±0.16
	-6.25m A	0.344	3.4%	0.27±0.07	4.490	44.3%	0.23±0.07	1.57	15.5%	0.05±0.06	3.72	36.7%	-0.08±0.06	10.13	9.86	103%	0.09±0.16	+0.01±0.17
	-6.25m B	0.332	3.2%		4.774	46.0%		1.70	16.4%		3.57	34.4%		10.38	9.86	105%		
	-6.25m C	0.335	3.3%		4.406	44.0%		1.67	16.7%		3.60	36.0%		10.01	9.86	102%		
	-6.75m	0.417	4.5%	0.48±0.07	4.001	43.0%	0.31±0.07	1.56	16.8%	0.17±0.06	3.32	35.7%	0.09±0.06	9.30	12.20	76%	0.22±0.16	-0.13±0.17
	-7.25m	0.337	8.0%	0.48±0.07	1.733	41.3%	0.40±0.06	0.63	15.0%	0.19±0.05	1.50	35.7%	0.06±0.05	4.20	5.48	77%	0.26±0.15	-0.04±0.16
	-7.75m	0.127	4.8%	1.58±0.06	0.800	29.9%	0.51±0.09	0.47	17.5%	0.36±0.06	1.28	47.8%	0.16±0.09	2.67	2.73	98%	0.36±0.18	+0.10±0.19
	-8.25m	0.057	3.5%		0.030	1.8%		0.26	16.1%	0.29±0.06	1.28	78.6%	0.05±0.09	1.63	1.54	106%	0.09±0.14	-0.16±0.16
	-8.75m	0.006	3.0%		0.001	0.5%		0.06	26.5%		0.15	69.9%		0.21	0.22* (± 0.04)	95%		
	-9.25m	0.000	0.8%		0.000	0.0%		0.04	58.3%		0.03	40.9%		0.06	0.06* (± 0.00)	107%		
	-9.75m	0.001	0.8%		0.000	0.2%		0.04	57.4%		0.03	41.6%		0.07	0.08* (± 0.02)	84%		
	-10.25m	0.000	0.7%		0.000	0.0%		0.04	66.6%		0.02	32.7%		0.05	0.06* (± 0.01)	91%		
	-10.75m	0.000	0.2%		0.000	0.2%		0.03	74.9%		0.01	24.7%		0.04	0.05* (± 0.01)	83%		
	-11.25m	0.001	1.4%		0.000	0.0%		0.02	42.7%		0.03	55.9%		0.05	0.06* (± 0.01)	84%		
	-11.75m	0.000	0.4%		0.000	0.0%		0.01	20.4%		0.06	79.2%		0.07	0.05* (± 0.00)	149%		
	-12.75m	0.001	1.6%		0.000	0.2%		0.01	22.1%		0.04	76.1%		0.06	0.07* (± 0.00)	82%		
	-13.25m	0.006	7.1%		0.000	0.0%		0.02	26.0%		0.06	66.9%		0.09	0.12* (± 0.00)	77%		
	-13.75m	0.005	4.7%		0.000	0.0%		0.02	17.3%		0.08	78.0%		0.11	0.06* (± 0.00)	170%		
	-14.25m	0.003	6.1%		0.000	0.0%		0.01	29.8%		0.03	64.0%		0.05	0.05* (± 0.00)	97%		
	-14.75m	0.000	1.1%		0.000	0.0%		0.01	45.0%		0.02	53.9%		0.03	0.04* (± 0,00)	81%		
	-15.25m	0.000	0.3%		0.000	0.1%		0.02	38.3%		0.03	61.3%		0.04	0.04	109%		
	-15.75m	0.005	6.7%		0.000	0.0%		0.02	22.0%		0.05	71.3%		0.08	0.11	72%		

*Table S7.4: SEP Hg concentration, fraction, and stable isotope data from **SCB2**. Values A, B, C are replicate extractions. THg of low concentration samples marked with '*' were analysed with a DMA-80 (Milestone) Hg analyser in triplicates (SD reported in brackets).*

	Sample	F1 extract Hg conc. (mg kg^{-1})	F1 Fraction (%)	F1 δ^{202}Hg (‰)	F2 extract Hg conc. (mg kg^{-1})	F2 Fraction (%)	F2 δ^{202}Hg (‰)	F3 extract Hg conc. (mg kg^{-1})	F3 Fraction (%)	F3 δ^{202}Hg (‰)	F4 extract Hg conc. (mg kg^{-1})	F4 Fraction (%)	F4 δ^{202}Hg (‰)	ΣSEP Extract THg conc. (mg kg^{-1})	Measured bulk THg Conc. ww (mg kg^{-1})	Recovery ΣSEP THg to ww THg (%)	δ^{202}Hg ΣSEP extracts (‰)	δ^{202}Hg difference: ΣSEP extracts to measured bulk (‰)
SCB2	-1.6m	0.012	0.8%		0.004	0.3%		0.429	26.4%		1.18	72.6%		1.63	1.61* (± 0.27)	101%		
	-1.8m	0.006	0.5%		0.002	0.2%		0.343	28.1%		0.87	71.2%		1.22	1.98	61%		
	-2m	0.007	0.6%		0.001	0.1%		0.319	29.0%		0.77	70.2%		1.10	1.09	101%		
	-2.2m	0.001	0.2%		0.000	0.1%		0.149	38.4%		0.24	61.4%		0.39	0.48	81%		
	-2.6m	0.003	0.3%		0.013	1.4%		0.458	48.7%		0.47	49.6%		0.94	0.66* (± 0.32)	143%		
	-2.8m	0.001	0.3%		0.001	0.5%		0.093	38.5%		0.15	60.7%		0.24	0.28	86%		
	-3.2m	0.002	0.1%		0.004	0.3%		0.598	44.5%		0.74	55.1%		1.35	1.46	92%		
	-4.25m	0.017	0.2%		0.066	0.8%		2.263	26.7%		6.13	72.3%		8.48	8.36	101%		
	-6.75m	0.002	0.5%		0.062	13.8%		0.114	25.2%		0.27	60.5%		0.45	0.32* (± 0.02)	140%		
	-7.75m	0.000	0.3%		0.000	0.2%		0.057	48.1%		0.06	51.4%		0.12	0.08* (± 0.02)	142%		
	-8.75m	0.206	4.0%	0.22±0.09	0.273	5.3%	0.31±0.09	2.142	41.9%	0.28±0.06	2.49	48.8%	0.07±0.03	5.11	5.37	95%	0.18±0.18	+0.05±0.20
	-9.25m	0.482	3.4%	0.34±0.07	2.990	20.8%	0.48±0.07	3.369	23.4%	0.27±0.08	7.54	52.4%	0.07±0.05	14.38	11.93	121%	0.16±0.21	+0.08±0.17
	-9.75m	0.606	3.3%	0.26±0.07	7.683	42.0%	0.27±0.07	3.739	20.5%	0.00±0.08	6.25	34.2%	-0.18±0.05	18.28	20.28	90%	0.16±0.06	+0.12±0.18
	-10.15m	0.600	3.1%	0.34±0.07	3.987	20.5%	0.37±0.07	4.909	25.2%	0.20±0.08	9.99	51.3%	-0.07±0.05	19.49	16.80	116%	0.16±0.10	+0.11±0.17
	-10.4m	0.200	1.3%	1.66±0.07	3.010	19.5%	0.49±0.07	4.215	27.2%	0.30±0.05	8.05	52.0%	0.15±0.05	15.47	17.49	88%	0.15±0.28	+0.42±0.17
	-10.6m	0.160	1.1%	2.71±0.15	2.435	16.5%	0.47±0.07	3.942	26.7%	0.19±0.05	8.20	55.6%	0.07±0.03	14.74	17.79	83%	0.19±0.19	+0.27±0.20
	-10.85m	0.175	1.3%	1.87±0.15	3.917	29.9%	0.50±0.07	3.484	26.6%	0.07±0.05	5.53	42.2%	0.00±0.03	13.11	18.51	71%	0.19±0.19	+0.32±0.21
	-11.15m	0.200	1.2%	1.30±0.15	4.904	28.5%	0.32±0.08	4.141	24.1%	0.04±0.05	7.96	46.3%	-0.08±0.03	17.21	15.23	113%	0.20±0.08	+0.27±0.20
	-11.45m	0.239	1.5%	0.81±0.07	4.910	31.4%	0.33±0.08	3.845	24.6%	0.02±0.05	6.63	42.4%	-0.16±0.03	15.62	18.94	83%	0.15±0.06	+0.18±0.17
	-11.75m	0.419	1.5%	0.26±0.07	14.778	53.0%	0.13±0.08	5.982	21.5%	-0.17±0.05	6.68	24.0%	-0.32±0.03	27.86	29.74	94%	0.15±-0.04	+0.15±0.16
	-12.05m A	0.337	1.4%	0.42±0.07	11.075	45.2%	0.16±0.08	5.870	24.0%	-0.20±0.05	7.22	29.5%	-0.31±0.03	24.50	26.54	92%	0.15±-0.06	+0.10±0.17
	-12.05m B	0.378	1.6%		10.925	45.9%		5.418	22.8%		7.07	29.7%		23.79	26.54	90%		
	-12.05m C	0.361	1.4%		10.562	41.5%		5.456	21.4%		9.08	35.7%		25.46	26.54	96%		
	-12.35m A	0.217	0.9%	0.86±0.07	7.640	31.9%	0.47±0.08	6.333	26.4%	0.11±0.05	9.79	40.8%	-0.04±0.03	23.98	23.16	104%	0.15±0.17	+0.45±0.16
	-12.35m B	0.201	1.1%		5.560	29.3%		4.392	23.2%		8.81	46.4%		18.96	23.16	82%		
	-12.35m C	0.219	1.2%		4.554	25.5%		4.001	22.4%		9.08	50.9%		17.85	23.16	77%		
	-12.65m A	0.229	1.4%	0.51±0.07	1.229	7.5%	0.13±0.08	2.836	17.3%	0.09±0.05	12.12	73.8%	-0.08±0.03	16.41	34.33	48%	0.15±-0.03	+0.15±0.17
	-12.65m B	0.190	1.2%	0.41±0.09	1.477	8.9%	0.27±0.06	5.304	32.1%	0.17±0.06	9.57	57.9%	-0.05±0.06	16.54	34.33	48%	0.17±0.05	+0.23±0.19
	-12.95m	0.186	1.2%	1.10±0.15	7.322	45.5%	0.18±0.08	3.949	24.6%	-0.15±0.05	4.63	28.8%	-0.18±0.03	16.08	13.15	122%	0.2±0	+0.17±0.20
	-13.25m A	0.353	1.3%	0.38±0.07	13.00	47.5%	0.06±0.08	5.904	21.6%	-0.20±0.05	8.14	29.7%	-0.27±0.03	27.40	15.81	173%	0.15±-0.09	+0.08±0.17
	-13.25m B	0.230	1.3%		9.145	51.0%		4.426	24.7%		4.14	23.1%		17.94	15.81	113%		
	-13.25m C	0.197	1.2%		8.097	48.1%		4.320	25.7%		4.22	25.1%		16.83	15.81	106%		
	-13.55m	0.199	1.2%	-0.02±0.09	4.351	26.9%	0.15±0.06	5.469	33.8%	-0.10±0.06	6.14	38.0%	-0.25±0.06	16.16	19.49	83%	0.17±-0.09	+0.03±0.18
	-13.85m	0.274	1.0%	0.35±0.09	8.051	30.8%	0.61±0.06	8.161	31.3%	0.33±0.06	9.62	36.8%	0.05±0.06	26.10	16.50	158%	0.17±0.32	+0.38±0.19
	-14.15m	0.010	0.8%		0.037	2.9%		0.608	48.7%		0.59	47.6%		1.25	0.87	143%		
	-14.75m	0.003	0.7%		0.002	0.6%		0.153	40.0%		0.22	58.7%		0.38	0.28	136%		

*Table S7.5: SEP Hg concentration, fraction, and stable isotope data from **SCB3**. THg of low concentration samples marked with '*' were analysed with a DMA-80 (Milestone) Hg analyser in triplicates (SD reported in brackets).*

Sample	F1 extract	F2 extract	F3 extract	F4 extract	ΣSEP Extract	Measured bulk	Recovery	δ^{202}Hg	δ^{202}Hg difference:

		Hg conc. (mg kg^{-1})	Fraction (%)	$\delta^{202}Hg$ (‰)	Hg conc. (mg kg^{-1})	Fraction (%)	$\delta^{202}Hg$ (‰)	Hg conc. (mg kg^{-1})	Fraction (%)	$\delta^{202}Hg$ (‰)	Hg conc. (mg kg^{-1})	Fraction (%)	$\delta^{202}Hg$ (‰)	THg conc. (mg kg^{-1})	THg Conc. ww (mg kg^{-1})	ΣSEP THg to ww THg (%)	ΣSEP extracts (‰)	ΣSEP extracts to measured bulk (‰)
SCB3	-0.5m	0.002	0.9%		0.000	0.0%		0.083	41.3%		0.12	57.8%		0.20	0.21* (± 0.02)	96%		
	-1.5m	0.002	1.3%		0.001	0.4%		0.060	36.4%		0.10	62.0%		0.16	0.15* (± 0.02)	107%		
	-2.4m	0.002	1.2%		0.001	1.1%		0.046	36.6%		0.08	61.1%		0.13	0.14* (± 0.02)	91%		
	-3.05m	0.000	0.5%		0.000	0.3%		0.039	67.9%		0.02	31.3%		0.06	0.06* (± 0.00)	97%		
	-3.65m	0.000	0.9%		0.020	46.6%		0.016	37.4%		0.01	15.0%		0.04	0.07* (± 0.02)	63%		
	-4.35m	0.000	0.0%		0.004	27.9%		0.008	54.6%		0.00	17.5%		0.01	0.02* (± 0.00)	92%		
	-5.65m	0.296	14.1%	1.08±0.07	0.132	6.3%	0.28±0.03	0.510	24.3%	0.35±0.07	1.16	55.3%	0.46±0.07	2.10	2.10	100%	0.51±0.15	+0.08±0.17
	-6.3m	2.142	23.3%	0.46±0.07	3.670	39.9%	0.13±0.07	1.415	15.4%	0.17±0.07	1.98	21.5%	0.18±0.06	9.21	9.91	93%	0.22±0.17	-0.12±0.18
	-7.05m	0.849	6.4%	0.40±0.15	7.412	56.2%	-0.03±0.07	1.742	13.2%	-0.03±0.07	3.19	24.2%	-0.05±0.06	13.19	13.88	95%	0.00±0.21	-0.07±0.22
	-7.75m A	0.499	2.9%	0.27±0.07	11.163	65.2%	-0.10±0.07	2.328	13.6%	-0.04±0.07	3.13	18.3%	-0.11±0.06	17.12	17.87	96%	-0.08±0.16	-0.08±0.17
	-7.75m B	0.503	2.7%		11.290	60.5%		2.329	12.5%		4.52	24.3%		18.65	17.87	104%		
	-7.75m C	0.481	2.7%		11.520	64.7%		2.388	13.4%		3.41	19.2%		17.80	17.87	100%		
	-8.25m	0.548	5.4%	0.39±0.03	5.285	51.9%	0.45±0.07	1.417	13.9%	0.04±0.07	2.94	28.8%	0.00±0.07	10.19	10.67	95%	0.31±0.15	+0.15±0.16
	-8.75m	0.288	3.2%	0.31±0.03	5.074	56.0%	-0.08±0.07	1.203	13.3%	0.32±0.07	2.49	27.5%	0.17±0.06	9.05	9.28	98%	0.06±0.15	-0.18±0.16
	-9.5m	0.320	3.1%	0.37±0.03	6.099	58.9%	-0.06±0.07	1.581	15.3%	0.03±0.07	2.35	22.7%	-0.03±0.06	10.35	9.42	110%	-0.02±0.15	-0.12±0.16
	-10.5m	0.256	2.2%	0.79±0.09	6.036	52.3%	-0.19±0.07	3.045	26.4%	-0.01±0.07	2.21	19.1%	-0.05±0.06	11.55	11.27	102%	-0.09±0.18	-0.24±0.19
	-11.5m A	0.075	2.7%		1.356	49.0%	0.47±0.07	0.290	10.5%	0.33±0.06	1.05	37.9%	0.17±0.06	2.77	2.78	100%	0.33±0.14	+0.04±0.15
	-11.5m B	0.074	2.6%		1.491	53.1%		0.312	11.1%		0.93	33.1%		2.81	2.78	101%		
	-11.5m C	0.079	2.9%		1.400	51.3%		0.315	11.6%		0.93	34.2%		2.73	2.78	98%		
	-12.5m	0.002	2.7%		0.000	0.3%		0.054	74.6%		0.02	22.4%		0.07	0.08* (±0.00)	86%		
	-13.5m	0.001	1.2%		0.000	0.6%		0.016	21.8%		0.06	76.4%		0.07	0.08* (±0.01)	95%		

Table S7.6: Relative standard deviation for replicate sequential extractions. In total 4 samples were extracted in duplicates and 6 samples in triplicates.

	F1	F2	F3	F4	Recovery
Average RSD	8.4 %	8.3 %	9.8%	19.7%	12.9 %
Min – Max RSD	2.3 – 24.4 %	2.4 – 28.4 %	1.8 – 42.4 %	7.1 – 53.2 %	0.3 – 40 %

Table S7.7: Analyses of PTD matrix Hg peak maximum temperature compared to SEP extraction fraction. The PTD temperature of the matrix Hg peak in these analyses is the maximum temperature measured for each sample from 190 – 300 °C and not data from the peak fitting analyses in Section S5.

Sample		PTD matrix Hg peak max temp (°C)	F1	F2	F3	F4
SCA2	-0.5m	226	0.1%	0.1%	47.8%	52.0%
	-1.5m	219	0.1%	0.4%	35.7%	63.8%
	-2.5m	245	0.4%	0.5%	37.1%	62.0%
	-3.5m	238	0.5%	0.9%	35.3%	63.3%
	-4.5m	232	0.3%	0.7%	37.9%	61.1%
	-5.5m	228	0.2%	1.2%	20.1%	78.5%
	-6.5m	236	2.3%	17.2%	27.8%	52.7%
	-7.5m	225	2.4%	4.6%	45.8%	47.2%
	-9.5m	264	2.0%	0.2%	66.9%	30.9%
	-11.5m	265	4.2%	2.5%	72.8%	20.6%
	-12.5m	270	1.4%	3.9%	67.4%	27.3%
	-13.5m	267	2.3%	9.1%	55.4%	33.2%
SCA1	-0.5m	241	0.5%	0.2%	22.2%	77.1%
	-1.5m	242	0.1%	1.7%	20.4%	77.8%
	-3.5m	251	0.6%	0.6%	24.1%	74.8%
	-4.5m	268	1.6%	32.4%	7.5%	58.4%
	-5.5m	267	0.4%	7.3%	19.4%	72.9%
	-6.5m	259	2.9%	45.5%	12.0%	39.6%
	-7.5m	287	1.0%	48.6%	21.1%	29.2%
	-8.5m	269	5.2%	0.0%	40.8%	54.1%
	-9.5m	285	0.9%	8.3%	42.7%	48.1%
SCB1	-0.25m	205	1.2%	1.4%	11.9%	85.6%
	-0.75m	197	0.8%	0.8%	14.4%	84.0%
	-1.25m	219	1.7%	18.0%	38.7%	41.6%
	-1.75m	237	1.7%	19.1%	37.5%	41.8%
	-2.25m	224	1.4%	17.4%	14.6%	66.5%
	-2.75m	217	2.3%	12.0%	26.7%	59.0%
	-3.25m	235	6.0%	5.6%	16.1%	72.2%
	-3.75m	216	3.3%	0.9%	10.0%	85.8%
	-4.25m	232	7.9%	2.4%	23.4%	66.4%
	-5.25m	225	5.1%	29.0%	17.1%	48.8%
	-5.75m	228	4.4%	47.4%	17.3%	31.0%
	-6.25m	254	3.3%	44.8%	16.2%	35.7%
	-6.75m	225	4.5%	43.0%	16.8%	35.7%
	-7.25m	237	8.0%	41.3%	15.0%	35.7%
	-7.75m	237	4.8%	29.9%	17.5%	47.8%
	-8.25m	245	3.5%	1.8%	16.1%	78.6%
SCB2	-5.65m	246	14.1%	6.3%	24.3%	55.3%
	-6.3m	244	23.3%	39.9%	15.4%	21.5%
	-7.05m	229	6.4%	56.2%	13.2%	24.2%
	-7.75m	223	2.8%	63.5%	13.2%	20.6%
	-8.25m	229	5.4%	51.9%	13.9%	28.8%
	-8.75m	237	3.2%	56.0%	13.3%	27.5%
	-9.5m	243	3.1%	58.9%	15.3%	22.7%
	-10.5m	275	2.2%	52.3%	26.4%	19.1%
	-11.5m	244	2.8%	51.1%	11.0%	35.1%
SCB3	-1.5m	252	0.6%	0.2%	27.8%	71.3%
	-3.5m	286	0.2%	0.7%	43.9%	55.1%
	-4.5m	274	0.2%	0.8%	26.7%	72.3%
	-8.5m	244	4.0%	5.3%	41.9%	48.8%
	-9.5m	255	3.3%	31.4%	21.9%	43.3%
	-10.5m	244	1.7%	21.6%	26.4%	50.3%
	-11.5m	251	1.4%	37.7%	23.4%	37.6%
	-12.5m	260	1.2%	31.7%	24.0%	43.1%

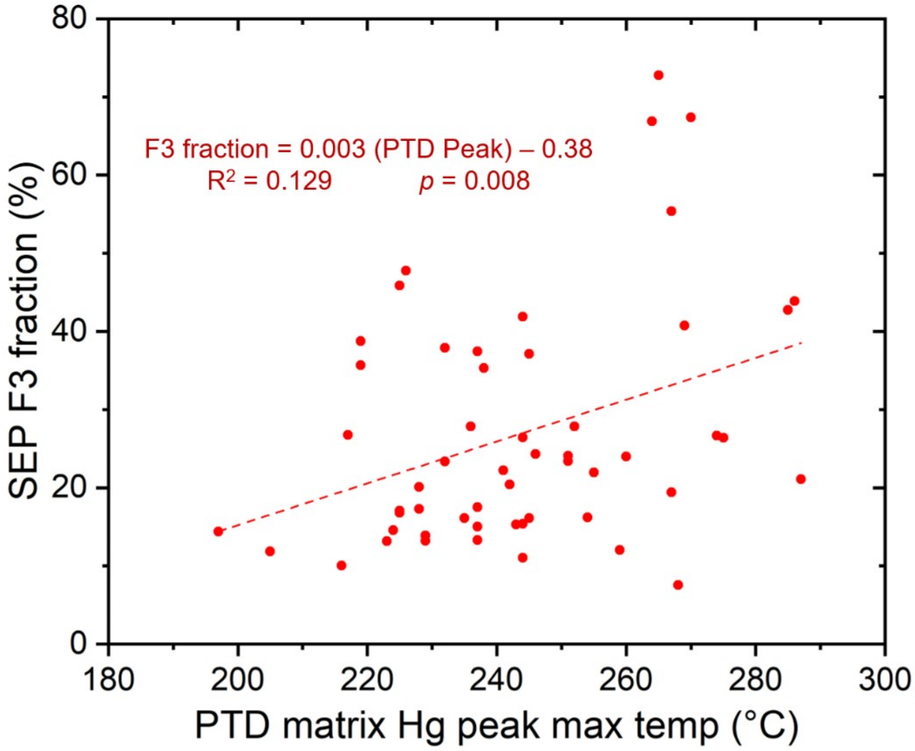

Figure S7.1: Relationship between SEP F3 fraction and PTD matrix Hg peak maximum release temperature for all solid phase soil core samples analysed by both SEP and PTD analyses. This shows a weak relationship between shifts in PTD matrix bound Hg peak towards the upper end of the range (≈190 – 300 °C) and an increasing proportion of SEP F3 in the solid phase samples.

S8. Total carbon, organic carbon, and inorganic carbon analyses of solid phase materials

Solid-phase organic carbon (OC) content was determined by first removing the inorganic carbon fraction with concentrated HCl, "carbonate-bomb" method (Müller and Gastner, 1971), combustion of the dried digestate, and analysis by infra-red detection of CO_2 released using a DIMA 1000NT (Dimatec, Germany). The total carbon (TC) content was determined by combusting and analysing the untreated dried material.

Table S8.1: Total carbon, organic carbon and inorganic carbon (determination by subtraction) analyses of solid phase materials. Detection limit for carbon measurements was ~500 mg kg^{-1}. nv denotes values below detection limits.

sample	depth (m)	TC mean (%)	TOC mean (%)	TIC (%)
SCB2	1.6	0.51	0.34	0.17
SCB2	2.6	0.76	0.28	0.48
SCB2	3.75	4.22	1.15	3.07
SCB2	4.75	4.28	1.26	3.01
SCB2	5.75	4.25	0.77	3.48
SCB2	6.75	3.76	0.81	2.95
SCB2	7.75	0.64	nv	
SCB2	8.75	0.11	nv	
SCB2	9.75	0.44	nv	
SCB2	10.6	0.04	nv	
SCB2	11.5	0.04	nv	
SCB2	12.7	0.05	nv	
SCB2	13.6	nv	nv	
SCB2	14.5	nv	nv	
SCA1	1.5	1.32	0.63	0.69
SCA1	5.5	nv		
SCA1	9.5	nv		
SCA1	13.5	nv		
SCA2	1.5	1.36	0.53	0.83
SCA2	5.5	1.15	0.49	0.66
SCA2	9.5	nv		
SCA2	13.5	nv		
SCA2	16.5	nv		
SCA2	20.5	nv		
SCA3	0.1	1.02		

S9. ICP-OES data from site B

Major metal cations were measured with inductively coupled plasma optical emission spectrometry (ICP-OES). Samples for ICP-OES were prepared by adding 0.5 g of sample to 50 mL vials capped loosely filled with 12 mL aqua regia. Samples were digested for 2 h at 85 °C on a heating plate. Aqua regia was then refilled to 12 mL and the samples further digested for 1 h. Then samples were filled to 50 mL with deionized water and filtered through 0.45 µm cellulose acetate filters and finally diluted with 2 % HNO_3 before analysis.

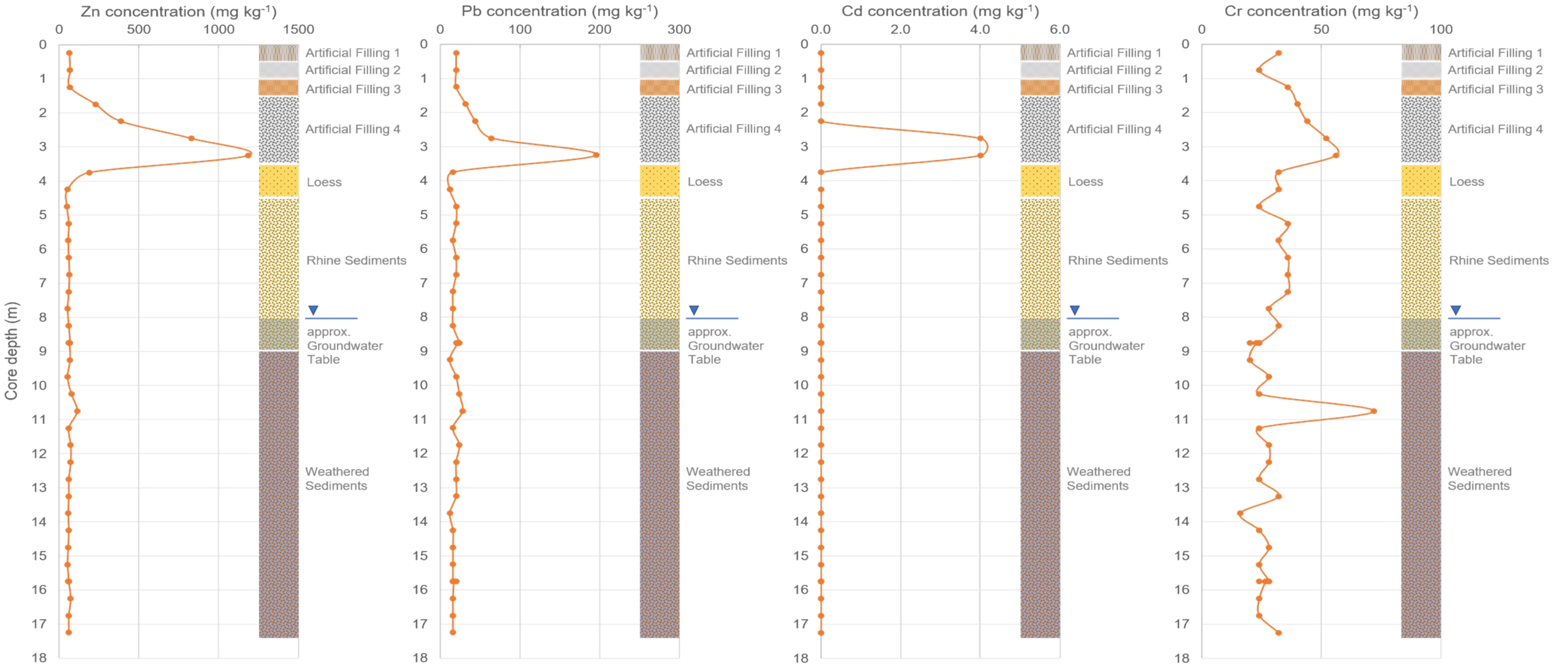

Figure S9.1: ICP-OES data of other trace metals (zinc, lead, cadmium, and chromium) in SCB1. Graphs show evidence of trace metal enrichment at the bottom of the lower artificial filling layer (Artificial Filling 4), above the loess layer. This provides supportive evidence of "ponding" that likely occurs during rainfall or snowmelt events creating a temporary aquifer where soluble trace metals would accumulate by leaching through the overlying artificial filling layers.

*Table S9.1: ICP-OES data from aqua regia extract of **SCB1**. "nv" denotes "no-value", which is below instrument detection limits. THg concentrations and pH data are from Section S6 (not ICP-OES analyses) and included for comparative purposes. For samples with replicate digests (n=3) the standard deviation is reported in brackets.*

	Sample	Ag (mg kg^{-1})	Al (mg kg^{-1})	As (mg kg^{-1})	Ca (mg kg^{-1})	Cd (mg kg^{-1})	Co (mg kg^{-1})	Cr (mg kg^{-1})	Cu (mg kg^{-1})	Fe (mg kg^{-1})	K (mg kg^{-1})	Mg (mg kg^{-1})	Mn (mg kg^{-1})	Na (mg kg^{-1})	Ni (mg kg^{-1})	P (mg kg^{-1})	Pb (mg kg^{-1})	Tl (mg kg^{-1})	Zn (mg kg^{-1})	THg (mg kg^{-1})	pH
SCB1	-0.25m	nv	11800	28	98400	nv	nv	32	24	17400	1328	10600	552	136	24	500	20	nv	64	66.29	7.4
	-0.75m	nv	11400	8	87800	nv	nv	24	28	17800	1444	12600	552	132	24	556	20	nv	68	18.45	7.5
	-1.25m	nv	11200	96	118000	nv	nv	36	16	16400	1956	13800	496	272	20	496	20	nv	68	515.7	8.7
	-1.75m	nv	20600	84	109200	nv	nv	40	16	22800	2528	8600	716	3252	16	464	32	nv	228	251.8	11.3
	-2.25m	nv	19400	88	114600	nv	nv	44	12	18800	2492	9600	640	2584	16	472	44	nv	388	267.0	11.3
	-2.75m	nv	12600	80	127400	4	nv	52	16	16600	2180	14400	516	848	20	472	64	nv	828	562.3	11.1
	-3.25m	nv	12200	88	130800	4	nv	56	16	18800	2212	14800	508	752	20	484	196	nv	1184	415.5	11.2
	-3.75m	nv	8800	152	140800	nv	nv	32	16	15400	1492	19200	520	232	20	512	16	nv	188	26.45	8.2
	-4.25m	nv	12200	216	90000	nv	nv	32	20	19400	1220	13400	400	392	20	560	12	nv	52	2.06	7.6
	-4.75m	nv	9800	116	1920	nv	nv	24	8	18400	1124	4800	440	348	12	456	20	nv	48	0.37	7.0
	-5.25m	nv	13600	140	2384	nv	nv	36	12	25200	2112	6800	448	180	16	548	20	nv	60	3.61	7.0
	-5.75m	nv	10600	136	2196	nv	nv	32	12	22600	1580	5200	388	112	12	540	16	nv	56	6.31	7.1
	-6.25m	nv	12800	144	2272	nv	nv	36	16	25600	2236	6400	452	124	16	552	20	nv	60	10.09	6.8
	-6.75m	nv	13600	156	3104	nv	nv	36	12	27200	2552	6400	484	132	16	824	20	nv	64	12.51	6.8
	-7.25m	nv	13400	72	2240	nv	nv	36	12	23600	2432	6200	396	128	16	572	16	nv	60	5.63	6.8
	-7.75m	nv	11800	56	2400	nv	nv	28	12	21600	1520	5400	376	100	12	596	16	nv	52	2.81	6.8
	-8.25m	nv	13600	40	2560	nv	nv	32	12	23600	1644	6400	392	108	16	608	16	nv	60	1.59	6.7
	-8.75m	nv	14067 (± 231)	29 (± 2)	2749 (± 44)	nv	nv	23 (± 2)	12 (± 0)	22200 (± 1744)	1460 (± 38)	5600 (± 346)	444 (± 444)	92 (± 4)	15 (± 2)	663 (± 44)	23 (± 2)	nv	64 (± 4)	0.16	6.4
	-9.25m	nv	18200	24	3200	nv	nv	20	12	23200	3348	7200	416	144	16	720	12	nv	68	0.05	6.4
	-9.75m	nv	16200	24	3176	nv	nv	28	12	23800	2388	6200	380	100	16	948	20	nv	52	0.04	6.4
	-10.25m	nv	17000	24	2036	nv	nv	24	16	24800	3348	6600	356	112	12	408	24	nv	76	0.04	6.3
	-10.75m	nv	14400	56	3200	nv	nv	72	20	20800	4760	7000	804	188	28	2000	28	nv	112	0.04	6.4
	-11.25m	nv	18400	32	2520	nv	nv	24	12	23600	1392	4800	236	92	12	568	16	nv	60	0.04	6.3
	-11.75m	nv	18000	52	2868	nv	nv	28	16	29000	1244	5000	292	104	16	720	24	nv	72	0.03	6.3
	-12.25m	nv	21400	32	4400	nv	nv	28	16	28600	2992	6400	440	132	16	1436	20	nv	72	0.04	6.3
	-12.75m	nv	16200	36	2476	nv	nv	24	12	24200	1604	4400	352	96	16	664	20	nv	60	0.04	6.3
	-13.25m	nv	19200	36	3052	nv	nv	32	16	25400	1884	5000	380	104	16	744	20	nv	60	0.06	6.2
	-13.75m	nv	16600	16	2644	nv	nv	16	12	21400	1528	4200	264	100	12	600	12	nv	56	0.04	6.2
	-14.25m	nv	17000	28	2512	nv	nv	24	16	20800	1792	4400	300	100	12	528	16	nv	60	0.03	6.2
	-14.75m	nv	16400	24	2372	nv	nv	28	16	21600	2192	4400	320	92	12	512	16	nv	56	0.03	6.2
	-15.25m	nv	16200	24	2428	nv	nv	24	12	20200	1828	4400	332	88	12	584	16	nv	52	0.03	6.2
	-15.75m	nv	17733 (± 643)	25 (± 2)	2717 (± 166)	nv	nv	27 (± 2)	13 (± 2)	22600 (± 1510)	1796 (± 167)	4667 (± 116)	353 (± 19)	91 (± 2)	15 (± 2)	648 (± 83)	17 (± 2)	nv	59 (± 2)	0.04	6.1
	-16.25m	nv	15600	28	3200	nv	nv	24	36	24600	1720	3868	1004	72	24	640	16	nv	72	0.17	6.1
	-16.75m	nv	16400	24	2740	nv	nv	24	12	21000	1660	4600	364	88	16	620	16	nv	60	0.05	6.1
	-17.25m	nv	17000	28	2844	nv	nv	32	12	22800	1632	5000	416	92	16	664	16	nv	60	0.04	6.1

*Table S9.2: ICP-OES data from **SCB2**. "nv" denotes "no-value", which is below instrument detection limits. THg concentrations and pH data are from Section S6 (not ICP-OES analyses) and included for comparative purposes. For samples with replicate digests (n=3) the standard deviation is reported in brackets.*

	Sample	Ag (mg kg^{-1})	Al (mg kg^{-1})	As (mg kg^{-1})	Ca (mg kg^{-1})	Cd (mg kg^{-1})	Co (mg kg^{-1})	Cr (mg kg^{-1})	Cu (mg kg^{-1})	Fe (mg kg^{-1})	K (mg kg^{-1})	Mg (mg kg^{-1})	Mn (mg kg^{-1})	Na (mg kg^{-1})	Ni (mg kg^{-1})	P (mg kg^{-1})	Pb (mg kg^{-1})	Tl (mg kg^{-1})	Zn (mg kg^{-1})	THg (mg kg^{-1})	pH
SCB2	-1.6m	nv	15200	12	6000	nv	nv	24	16	21200	1576	4800	860	140	24	548	56	nv	72	1.75	7.3
	-1.8m	nv	12800	8	6000	nv	nv	28	16	17800	1644	4200	820	144	28	560	56	nv	72	2.16	7.3
	-2m	nv	13600	8	26000	nv	nv	28	16	19800	1680	7000	792	164	28	596	52	nv	76	0.79	7.5
	-2.2m	nv	11000	12	39800	nv	nv	24	12	16600	1436	7800	632	176	24	544	40	nv	56	0.46	7.6
	-2.4m	nv	13400	12	20600	nv	nv	24	12	19400	1592	6200	676	204	24	588	32	nv	60	0.12	7.5
	-2.6m	nv	16000	12	7200	nv	nv	32	16	21400	1944	5000	816	128	28	640	20	nv	60	2.01	7.3
	-2.8m	nv	16600	12	10000	nv	nv	32	16	22400	1960	5400	820	120	28	656	20	nv	60	0.31	7.3
	-3m	nv	17000	12	20200	nv	nv	28	16	23600	1860	6800	708	116	28	612	20	nv	60	2.35	7.3
	-3.2m	nv	17800	12	17800	nv	nv	32	16	24200	1984	6800	768	120	32	664	20	nv	60	1.62	7.3
	-3.4m	nv	10600	8	131000	nv	nv	24	12	17200	1220	17000	536	100	24	548	8	nv	36	0.18	7.6
	-3.75m	nv	11400	8	173000	nv	nv	20	12	18800	1224	21600	532	104	20	536	8	nv	36	0.25	7.6
	-4.25m	nv	11600	8	95000	nv	nv	24	12	17600	1500	13000	624	116	24	592	12	nv	48	9.25	7.3
	-4.75m	nv	10200	8	153000	nv	nv	20	12	17400	1164	18800	496	112	20	548	8	nv	36	0.75	7.6
	-5.25m	nv	9067 (± 1026)	8 (± 0)	128333 (± 12722)	nv	nv	20 (± 0)	12 (± 0)	15533 (± 1665)	1167 (± 43)	16467 (± 1617)	491 (± 37)	128 (± 4)	21 (± 2)	493 (± 10)	8 (± 0)	nv	33 (± 2)	0.07	7.8
	-5.75m	nv	7800	8	120000	nv	nv	20	8	13600	1052	17000	448	100	20	488	8	nv	32	0.35	7.8
	-6.25m	nv	8000	8	127000	nv	nv	20	12	15400	1052	15600	488	88	20	520	8	nv	36	0.23	7.8
	-6.75m	nv	8200	8	113000	nv	nv	16	12	15600	956	13600	412	80	20	500	8	nv	36	0.86	7.7
	-7.25m	nv	13400	20	66000	nv	nv	24	12	24000	1432	11800	380	84	20	592	16	nv	52	0.16	7.6
	-7.75m	nv	9600	24	23600	nv	nv	20	12	17800	1280	6400	508	88	16	604	16	nv	52	0.88	7.7
	-8.25m	nv	11400	28	7400	nv	nv	28	12	22000	1416	6200	432	96	16	588	20	nv	60	0.14	7.7
	-8.75m	nv	11000	24	4400	nv	nv	24	12	21400	1728	5600	536	104	16	532	20	nv	56	5.45	7.7
	-9.25m	nv	12400	32	5800	nv	nv	28	16	24800	2120	6200	544	112	16	620	16	nv	60	12.11	7.7
	-9.75m	nv	12000	28	8000	nv	nv	28	12	24600	1808	5800	408	92	16	540	20	nv	60	20.68	7.7
	-10.15m	nv	14000	28	5600	nv	nv	28	12	26000	2164	7000	520	116	16	660	20	nv	60	17.36	7.7
	-10.4m	nv	8800	28	2350	nv	nv	20	8	16800	1284	4000	332	72	12	472	16	nv	48	19.23	7.6
	-10.6m	nv	10800	104	2710	nv	nv	24	12	23400	1468	4800	492	88	16	536	20	nv	52	19.53	7.7
	-10.85m	nv	9000	24	2230	nv	nv	20	8	18000	1456	4000	360	76	12	500	16	nv	44	20.51	7.8
	-11.15m	nv	9200	32	1820	nv	nv	20	8	16600	1236	4200	332	76	12	436	16	nv	44	16.47	7.5
	-11.45m	nv	11200	28	2260	nv	nv	24	12	21200	1596	5200	380	92	12	512	16	nv	52	20.32	7.3
	-11.75m	nv	18600	36	3400	nv	nv	28	20	24800	2480	5600	316	92	20	708	28	nv	80	34.08	6.6
	-12.05m	nv	13067 (± 1890)	28 (± 0)	2821 (± 292)	nv	nv	23 (± 2)	12 (± 0)	19467 (± 3139)	1635 (± 158)	4467 (± 833)	332 (± 49)	77 (± 2)	16 (± 0)	552 (±39)	20 (±0)	nv	55 (± 2)	30.79	6.7
	-12.35m	nv	10800	28	2650	nv	nv	20	12	17800	1388	4000	368	68	12	592	16	nv	52	26.42	6.9
	-12.65m	nv	10800	28	2360	nv	nv	20	12	20600	1408	4000	412	76	12	468	16	nv	44	39.59	7.2
	-12.95m	nv	9800	24	2320	nv	nv	20	8	16200	1152	3800	328	76	12	524	20	nv	48	14.77	6.9
	-13.25m	nv	14200	32	2800	nv	nv	28	12	21000	1448	4800	384	88	16	608	20	nv	56	26.67	6.7
	-13.55m	nv	10000	20	2390	nv	nv	16	8	15000	1248	3704	364	72	12	596	16	nv	44	22.40	6.7
	-13.85m	nv	9400	24	2140	nv	nv	20	8	14600	1164	3844	308	68	12	452	12	nv	40	19.03	7.0
	-14.15m	nv	10400	12	1720	nv	nv	16	8	11000	888	2600	148	64	8	316	12	nv	36	1.12	6.5
	-14.45m	nv	10200	8	1630	nv	nv	12	4	10200	800	2464	108	60	8	284	12	nv	36	0.88	6.3
	-14.75m	nv	14200	12	2120	nv	nv	20	8	16400	1112	4112	208	84	12	420	12	nv	44	0.70	6.4
	-15.05m	nv	17200	12	2610	nv	nv	28	8	20400	1260	5600	256	88	16	476	16	nv	60	0.10	6.5

Table S9.3: ICP-OES data from ***SCB3****. "nv" denotes "no-value", which is below instrument detection limits. THg concentrations and pH data are from Section S6 (not ICP-OES analyses) and included for comparative purposes. For samples with replicate digests (n=3) the standard deviation is reported in brackets.*

Sample		Ag (mg kg^{-1})	Al (mg kg^{-1})	As (mg kg^{-1})	Ca (mg kg^{-1})	Cd (mg kg^{-1})	Co (mg kg^{-1})	Cr (mg kg^{-1})	Cu (mg kg^{-1})	Fe (mg kg^{-1})	K (mg kg^{-1})	Mg (mg kg^{-1})	Mn (mg kg^{-1})	Na (mg kg^{-1})	Ni (mg kg^{-1})	P (mg kg^{-1})	Pb (mg kg^{-1})	Tl (mg kg^{-1})	Zn (mg kg^{-1})	THg (mg kg^{-1})	pH
SCB3	-0.5m	nv	13800	8	40200	nv	nv	36	108	18400	1968	8400	736	84	28	668	28	nv	100	0.17	7.7
	-1.5m	nv	8400	8	103000	nv	nv	20	12	13600	1100	5200	432	88	20	400	12	nv	36	0.06	7.7
	-2.4m	nv	6800	8	122000	nv	nv	16	8	12000	940	4600	344	100	16	372	12	nv	28	0.02	7.8
	-3.05m	nv	26600	12	8600	nv	nv	52	208	33600	2988	7800	1016	92	48	820	28	nv	160	0.02	7.4
	-3.65m	nv	11600	8	176000	nv	nv	24	20	19800	1224	21400	524	92	20	604	8	nv	48	0.02	7.8
	-4.35m	nv	17000	24	64000	nv	nv	32	24	28400	1716	13800	432	96	24	716	20	nv	68	0.23	7.6
	-5.35m	nv	16000	28	3210	nv	nv	32	16	28000	2348	7600	516	116	16	796	20	nv	64	2.13	7.2
	-6.3m	nv	12000	32	2430	nv	nv	20	12	21200	1580	5200	984	96	16	588	16	nv	56	10.13	7.0
	-7.05m	nv	16200	36	3470	nv	nv	32	16	29400	2332	7200	644	128	20	884	24	nv	68	14.21	7.0
	-7.75m	nv	19333 (± 462)	41 (± 2)	4133 (± 116)	nv	nv	33 (± 2)	32 (± 0)	29933 (± 808)	2984 (± 58)	6533 (± 116)	500 (± 14)	100 (± 0)	24 (± 0)	925 (± 29)	24 (± 0)	nv	96 (± 7)	19.93	6.8
	-8.25m	nv	15400	36	2640	nv	nv	32	16	25600	2592	6200	400	104	16	620	20	nv	64	10.95	6.6
	-8.75m	nv	14600	28	3600	nv	nv	28	12	24000	1872	5800	408	104	16	896	16	nv	60	9.85	6.6
	-9.5m	nv	17200	36	2940	nv	nv	32	16	26400	2172	6400	420	112	16	712	20	nv	64	10.14	6.5
	-10.5m	nv	14400	32	2480	nv	nv	28	16	22400	2020	5200	696	108	16	584	20	nv	56	12.04	6.5
	-11.5m	nv	19800	44	3070	nv	nv	32	16	25800	1660	5600	348	116	16	672	24	nv	64	2.99	6.5
	-12.5m	nv	19200	28	2580	nv	nv	28	24	23000	1640	4800	236	108	16	528	20	nv	68	0.05	6.3
	-13.5m	nv	20400	24	2610	nv	nv	28	16	25600	1996	5200	224	108	16	552	20	nv	64	0.05	6.3
	-14.5m	nv	22400	28	3430	nv	nv	32	16	29600	2008	5600	244	112	16	856	20	nv	68	0.02	6.3

References

Bollen, A., Wenke, A., & Biester, H. (2008). Mercury speciation analyses in $HgCl_2$-contaminated soils and groundwater—implications for risk assessment and remediation strategies. *Water Res.*, 42(1-2), 91-100.

Bloom, N. S., Preus, E., Katon, J., & Hiltner, M. (2003). Selective extractions to assess the biogeochemically relevant fractionation of inorganic mercury in sediments and soils. *Analyt. Chim. Acta*, *479*(2), 233-248.

Brocza, F. M., Biester, H., Richard, J. H., Kraemer, S. M., & Wiederhold, J. G. (2019). Mercury isotope fractionation in the subsurface of a Hg(II) chloride-contaminated industrial legacy site. *Environ. Sci. Technol.*, 53(13), 7296-7305.

Deutscher Wetterdienst (2021). Climate data Centre. Offenbach, Germany. Last visited: 17th May 2021. https://cdc.dwd.de/portal/

Hazen, R. M., Golden, J., Downs, R. T., Hystad, G., Grew, E. S., Azzolini, D., & Sverjensky, D. A. (2012). Mercury (Hg) mineral evolution: A mineralogical record of supercontinent assembly, changing ocean geochemistry, and the emerging terrestrial biosphere. *Amer. Mineralog.*, *97*(7), 1013-1042.

Landesanstalt für Umwelt Baden-Württemberg (2021). GuQ: Grundwasserstände und Quellschüttungen. Karlsruhe, Germany. Last visited: 17th May 2021. https://guq.lubw.baden-wuerttemberg.de/

Leopold, K., Foulkes, M., & Worsfold, P. (2010). Methods for the determination and speciation of mercury in natural waters—a review. *Analytica chimica acta*, *663*(2), 127-138.

Miller, C. L., Watson, D. B., Lester, B. P., Lowe, K. A., Pierce, E. M., & Liang, L. (2013). Characterization of soils from an industrial complex contaminated with elemental mercury. *Environ. Res.*, 125, 20-29.

Morel, F. M., Kraepiel, A. M., & Amyot, M. (1998). The chemical cycle and bioaccumulation of mercury. *Ann. Rev. Ecol. Systemat.*, *29*(1), 543-566.

Müller, G., & Gastner, M. (1971). The 'Karbonat-Bombe', a simple device for the determination of carbonate content in sediment, soils, and other materials. *Neues Jahrbuch für Mineralogie-Monatshefte*, *10*, 466-469.

Richard, J. H., Bischoff, C., Ahrens, C. G., & Biester, H. (2016a). Mercury (II) reduction and co-precipitation of metallic mercury on hydrous ferric oxide in contaminated groundwater. *Sci. Tot. Environ.*, *539*, 36-44.

Richard, J. H., Bischoff, C., & Biester, H. (2016b). Comparing modeled and measured mercury speciation in contaminated groundwater: Importance of dissolved organic matter composition. *Environ. Sci. Technol.*, *50*(14), 7508-7516.

Schöndorf, T., Egli, M., Biester, H., Mailahn, W., & Rotard, W. (1999). in *Mercury Contaminated Sites: characterization, risk assessment and remediation*, ed. R. Ebinghaus, R. R. Turner, L. D. Lacerda, O. Vasiliev and W. Salomons, Springer, Berlin, Heidelberg, 1st Edn., pp. 181–206.

Schöndorf, T. (2020). Personal communication. HBC AG, Freiburg, Germany.

Schroeder, W. H., & Munthe, J. (1998). Atmospheric mercury—an overview. *Atmos. Environ.*, *32*(5), 809-822.

Schuster, E. (1991). The behavior of mercury in the soil with special emphasis on complexation and adsorption processes-a review of the literature. *Water Air Soil Poll.*, *56*(1), 667-680.
